# Polydatin attenuates tubulointerstitial fibrosis in diabetic kidney disease by inhibiting YAP expression and nuclear translocation

**DOI:** 10.3389/fphys.2022.927794

**Published:** 2022-10-07

**Authors:** Manlin He, Lan Feng, Yang Chen, Bin Gao, Yiwei Du, Lu Zhou, Fei Li, Hongbao Liu

**Affiliations:** ^1^ Department of Nephrology, Tangdu Hospital, Air Force Military Medical University (Fourth Military Medical University), Xi’an, China; ^2^ Department of Endocrinology, Tangdu Hospital, Air Force Military Medical University (Fourth Military Medical University), Xi’an, China; ^3^ The Key Laboratory of Biomedical Information Engineering of Ministry of Education, School of Life Science and Technology, Xi’an Jiaotong University, Xi’an, China; ^4^ Bioinspired Engineering and Biomechanics Center (BEBC), Xi’an Jiaotong University, Xi’an, China

**Keywords:** polydatin, diabetic kidney disease, fibrosis, yes-associated protein, matrix stiffness

## Abstract

The activation of Yes-associated protein (YAP) pathway is mutually causal with the increase of extracellular matrix (ECM) stiffness. Polydatin (PD) has been proved to have anti-fibrosis effect in diabetic kidney disease (DKD), but it is still a mystery whether PD participates in YAP-related mechano-transduction. Therefore, this study intends to solve the following two problems: 1) To construct an *in vitro* system of polyacrylamide hydrogels (PA gels) based on the true stiffness of kidneys in healthy and DKD rats, and observe the effect of PD on pathological matrix stiffness-induced YAP expression in renal fibroblasts; 2) Compared with verteporfin (VP), a pharmacological inhibitor of YAP, to explore whether the therapeutic effect of PD on DKD *in vivo* model is related to the regulation of YAP. In this study, the *in vitro* system of PA gels with 3 kPa, 12 kPa and 30 kPa stiffness was constructed and determined for the first time to simulate the kidney stiffness of healthy rats, rats with DKD for 8 weeks and 16 weeks, respectively. Compared with the PA gels with 3 kPa stiffness, the PA gels with 12 kPa and 30 kPa stiffness significantly increased the expression of YAP, α-smooth muscle actin (α-SMA) and collagen I, and the production of reactive oxygen species (ROS) in renal fibroblasts, and the PA gels with 30 kPa stiffness were the highest. PD significantly inhibited the above-mentioned changes of fibroblasts induced by pathological matrix stiffness, suggesting that the inhibition of PD on fibroblast-to-myofibroblast transformation and ECM production was at least partially associated with regulating YAP-related mechano-transduction pathway. Importantly, the inhibitory effect of PD on YAP expression and nuclear translocation in kidneys of DKD rats is similar to that of VP, but PD is superior to VP in reducing urinary protein, blood glucose, blood urea nitrogen and serum creatinine, as well as decreasing the expression of α-SMA and collagen I, ROS overproduction and renal fibrosis. Our results prove for the first time from the biomechanical point of view that PD is a potential therapeutic strategy for delaying the progression of renal fibrosis by inhibiting YAP expression and nuclear translocation.

## Introduction

Fibrosis is a pathological process characterized by the excessive deposition of extracellular matrix (ECM), affecting almost every organ and eventually leading to organ dysfunction (Fu et al., 2020a). With the accumulation of ECM, the physical properties of tissues become more ‘stiff’, leading to the conversion of quiescent fibroblasts into active myofibroblasts, which in turn aggravates ECM deposition and induces fibrosis (Szeto et al., 2016). Fibroblasts can sense and adapt to changes in their mechanical environment, and transduce extracellular biomechanical signals into intracellular biochemical reactions and gene expression regulation (Emig et al., 2019; Wen et al., 2022) Several transcription factors regulated by mechanical stress have been proposed to be activators of myofibroblasts, including focal adhesion kinase (FAK), Wnt/β-catenin, and Hippo pathways, which have been reported to occur in mucosal and skin wounds or after injury to lung, heart, liver and kidney tissues (Wen et al., 2022).

Yes-associated protein (YAP), an effector of the canonical Hippo signaling pathway, can be activated by upstream mechanical signals (such as the increase in matrix stiffness), which promotes its nuclear translocation and targeted gene induction, and has emerged as a crucial factor in the pathogenesis of fibrosis in organs, including the kidney, liver, lung and cardiac tissue (Hu et al., 2021). A recent report confirmed that myofibroblast YAP was activated early and driven to fibrosis in the liver, lung and kidney injury in mice and humans (He et al., 2022). However, although we had profound insights into fibrosis at the cellular and molecular level, and had built different pathogenic models to mimic fibrogenesis, there were few satisfactory therapies and even fewer approaches focusing on mechano-regulation in clinical practice.

Polydatin (PD, C_20_H_22_O_8_) is a natural active ingredient extracted from the dried rhizomes and roots of *Polygonum cuspidatum* Sieb. et Zucc., a Polygonaceae plant that plays a protective role in animal models of pulmonary fibrosis (Cao et al., 2017; Tang et al., 2019; Zeng et al., 2019; Ali et al., 2022), hepatic fibrosis (Zhao et al., 2017; Li et al., 2018; Lan et al., 2020; Liu et al., 2020; Zhao et al., 2020; Tang et al., 2022) and renal fibrosis (Huang et al., 2015; Gong et al., 2017; Chen et al., 2020a). Its mechanism of anti-oxidative stress, anti-inflammation, anti-ferroptosis and regulation of autophagy has also been confirmed by us (Liu et al., 2015; Meng et al., 2015; Meng et al., 2016; Zhou et al., 2022) and other researchers (Gu et al., 2019; Xu et al., 2019; Abd El-Hameed, 2020; Chen et al., 2020a; Fu et al., 2020b; Gao et al., 2020; Han et al., 2020; Sun and Wang, 2020; Deng et al., 2021; Gu et al., 2021) in various kidney disorders. However, the role of PD in the fibroblast activation induced by pathological matrix stiffness has not been reported. Especially whether PD is involved in the regulation of YAP-related mechano-transduction is still a mystery.

Therefore, this study intends to construct polyacrylamide hydrogels (PA gels) with different matrix stiffness which can simulate the physical environment of renal fibrosis in diabetic kidney disease (DKD) rats, and then culture renal fibroblasts with or without PD to investigate the effects of PD on pathological matrix stiffness-induced fibroblast activation, YAP expression and reactive oxygen species (ROS) production. In addition, by comparing with verteporfin (VP), a pharmacological inhibitor of YAP, it is revealed whether the nephroprotective effect of PD on DKD rats is related to YAP regulation. This study is expected to provide a new idea for PD to treat renal fibrosis from the perspective of biomechanical regulation.

## Materials and methods

### Chemicals and reagents

Urine protein test kit (C035-2–1), urea nitrogen assay kit (C013-2–1) and creatinine assay kit (C001-2–1) were purchased from Nanjing Jiangcheng Bioengineering Institute (Nanjing, China). Acrylamide (AM, 92,560), N,N'-methylenebisacrylamide (MBA, M1533), Ammonium persulfate (APS, A3978), tetramethylethylenediamine (TEMED, T9281), Sulfosuccinimidyl 6-[(4'-azido-2'-nitrophenyl)amino]hexanoate (sulfo-SANPAH, 803332), 3-aminopropyltrimethosysilane (3-APTMS, 8.19172) and dimethyl sulfoxide (DMSO, D2650) were purchased from Sigma-Aldrich (St. Louis, MO, United States). Dulbecco’s modified Eagle’s medium/nutrient mixture F-12 (DMEM/F-12, 11330–032), fetal bovine serum (FBS, 10,099–141) and Triton X-100 (HFH-10) were purchased from Gibco (United States). Phosphate-buffered saline (PBS, P1020), hematoxylin-eosin (H&E) staining kit (G1120) and masson trichrome staining kit (G1340) were purchased from Solarbio (Beijing, China).

### Animals

All animal experiments were conducted in strict accordance with the principles of the “Guide for the Care and Use of Laboratory Animals” and were permitted by the Scientific Investigation Committee of the Air Force Military Medical University (Xi’an, China). Adult male Sprague-Dawley (SD) rats (8 weeks old) were purchased from the Experimental Animal Center of the Air Force Military Medical University and bred in an experimental animal room of SPF grade. After 1 week of adaptation, a high-fat diet (D12492, Ruidi, China) and low-dose intraperitoneal streptozocin (STZ, HY-13753, MedChemExpress, American) were used to establish the DKD model (Guo et al., 2021; Jiang et al., 2021; Tang et al., 2021). Briefly, rats were fed with a high-fat diet for 8 weeks and then received a single intraperitoneal injection of 40 mg/kg STZ. After 72 h of injection, the random blood glucose (RBG) > 16.7 mmol/L for more than 3 days indicated that the diabetic model was successfully induced. 24-h urine was collected from metabolic cages and urine protein (UP) was detected, when the 24-h UP was above 30 mg/24h, it indicated that the DKD model is successfully established. In addition, six rats were randomly selected to be fed with regular chow for 8 weeks and then given a single intraperitoneal injection of equivalent sodium citrate buffer (P4922, Merck, Germany) as control. Thereafter, after 8 or 16 weeks of continuous feeding with regular chow, the control and DKD animals were ethically sacrificed, and urine, whole blood and kidneys were collected for further analysis.

### Urine protein test, blood physiochemical assays and renal histology

Before sacrifice, 24-h urine was collected from the metabolic cage and then centrifuged at 4°C, 13,000 rpm for 10 min, and the UP level was measured according to the manufacturer’s instructions. The whole blood collected by cardiac puncture was centrifuged at 4°C, 14,000 rpm, for 15 min to acquire the serum samples, which were used for the blood urea nitrogen (BUN) and serum creatinine (Scr) according to the manufacturer’s instructions. Fresh renal tissues were washed with ice-cold stroke-physiological saline solution, fixed overnight with 4% paraformaldehyde, and then paraffin-embedded, followed cut into a thickness of 4 μm sections, which were prepared using H&E staining for observing kidney injury and Masson trichrome staining for evaluating renal fibrosis according to the manufacturer’s instructions. The severity of tubulointerstitial fibrosis was assessed by a renal pathologist who was unaware of the rat groups. According to the distribution of lesions, the findings were divided into 0–3 grades: 0, no lesion; 1, <20%; 2, 20–50%; 3. >50%. The scores were made by blinding in 10 consecutive fields with the magnification of 400× per slice. All tests were repeated three times.

### Renal tissue stiffness measurement

Kidney tissue was cut into 500 μm slices by vibration slice technology (LeicaVT1200S, Germany). The mechanical properties of the ECMs were measured using a micro-indentor designed to test the surface micro-stiffness of biological tissues (PIUMA, Optics11, Netherlands). Samples were indented in PBS with the PIUMA device, using a soft cantilever on the top of a ferruled optical fiber, with a radius of 30 μm and stiffness of 0.31 N/m. The indentations were depth controlled (15 μm) and the loading and unloading periods were set to 3 s. In each renal tissue slice, three matrices were randomly selected in the renal cortex area, and each matrix measured 9 points. Based on the load and displacement curves, the effective Young’s modulus was automatically calculated by the software from the PIUMA (Optics11, v1). A stiff surface was used before each series of tests for calibration.

### The isolation and identification of primary rat renal fibroblasts

The renal fibroblasts in SD rats were cultured by collagenase digestion and cell sieving, and improved according to the methods in the literature. The clipped kidney cortex was digested with type III collagenase (PM1083, Biotopped, Beijing, China) at 37°C for 5 min, then filtered through a 75 μm filter and the digest was collected. The above steps were repeated 3 times and the collected filtrate was centrifuged at 1000 rpm for 5 min. The cells were incubated in an enrichment medium (DMEM/F12 containing 15% FBS, glutamine and penicillin/streptomycin) for 2–4 days until the monolayer had covered 75% of the glass dish. Cells were characterized as fibroblasts by immunofluorescence staining for alpha-smooth muscle actin (α-SMA) and vimentin.

### Preparation of polyacrylamide hydrogels

The polyacrylamide hydrogels (PA gels) were prepared by measuring the stiffness of the renal cortex. The experimental procedure followed the detailed PA gel preparation method described previously (Tse and Engler, 2010). Laser confocal petri dishes were generated by incubating with NaOH followed by the addition of 3-APTMS, to serve as receptacles for the polymerized gels. AM (50%), MBA (1.25%), APS (10%) and TEMED were used for the polymerization of the hydrogel. Then, the polymerization solution was covered with a chlorosilane glass slide, which was removed after the polymerization for 30 min. Sulfo-SANPAH was added to PA gels and cross-linked at 365-nm UV light for 10 min. Fibronectin (354008, 4 μg/ml, Corning, United States) was added to cover the surface of PA gels and incubated at 4°C overnight. The Young’s moduli of these PA gels were measured with nanoindentation*.*

### *In vitro* treatment of polydatin

Centrifuge the PD (MB5448, Meilun Biotech, Dalian, China) powder (10,000 rpm, 2 min) to avoid the powder sticking to the tube wall. After centrifugation, DMSO is added to fully dissolve it, and it is stored in a refrigerator at -80°C in the dark. Renal fibroblasts cultured on the PA gels with different matrix stiffness were treated with 40 μM PD for 24 h.

### *In vivo* treatment of polydatin

To evaluate the role of PD in the activation of YAP in kidneys of DKD rats, rats were randomly divided into four groups:

- Control group (n = 6) and DKD group (n = 6): age-matched healthy SD rats and DKD rats were administered equivalent vehicle containing 0.5% sodium carboxymethylcellulose (CMC-Na, i. g. daily) and equivalent saline containing 0.5% dimethyl sulfoxide (DMSO, i. p. every other day) for 8 weeks.
- VP group (n = 6): the DKD rats were administered VP (a pharmacological inhibitor of YAP, 100 mg/kg dissolved in 0.5% DMSO, i. p. every other day) and equivalent vehicle containing 0.5% CMC-Na (i.g. daily) for 8 weeks.
- PD group (n = 6): the DKD rats were administered PD (100 mg/kg dissolved in 0.5% CMC-Na, i. g. daily) and equivalent saline containing 0.5% DMSO (i.p. every other day) for 8 weeks.

The dosages of PD and VP are consistent with a previous report (Chen et al., 2020a; Chen et al., 2020b). The rats were then sacrificed.

### Immunofluorescence and immunohistochemistry staining

Renal fibroblasts were seeded on laser confocal petri dishes lined with PA gels and cultured at 37 °C with 5% CO_2_ (with PD or without PD) for 24 h. The cells were fixed with 4% paraformaldehyde at room temperature for 15 min. The fixed cells were permeabilized for 20 min with 0.5% Triton X-100 before being gently washed and blocked in 4% bovine serum albumin at room temperature for 30 min. The following antibodies were used: α-SMA antibody (ab124964, Abcam, United States), vimentin antibody (BF8006, Affinity, United States), collagen Ⅰ antibody (AF7001, Affinity, United States) and YAP antibody (#14074, Cell Signaling Technology, United States). Alexa Fluor 594 goat anti-rabbit antibody (A10438, Thermo scientific, United States) was used as the secondary antibody. Amanita phalloidin (40762ES75, YEASEN, Shanghai China) was used to stain the actin cytoskeleton. Cell nuclei were visualized with 4',6-diamidino-2-phenylindole (DAPI, 10236276001, Roche, Switzerland). The fluorescence was immediately observed with a confocal laser-scanning microscope (CLSM, Nikon, Tokyo, Japan).

Kidney cryosections with a thickness of 4 μm were prepared and fixed in 4% paraformaldehyde for 15 min. After blocking, the cryosections were incubated with an anti-YAP (1:100, #14074, Cell Signaling Technology, United States) primary antibody and then with a fluorescein FITC-conjugated secondary antibody (GB22303, Servicebio Technology, China). For immunohistochemical analysis, after dewaxing, rehydration, antigen retrieval and blocking, the sections were incubated with an anti-α-SMA (1:1000, ab124964, Abcam, United States) or anti-collagen Ⅰ (1:1000, AF7001, Affinity, United States) primary antibody and then with a horseradish peroxidase-labelled secondary antibody (Beyotime, Shanghai, China).

### Western blot analysis

We carried out western blot analyses to detect target protein levels in kidney tissues and cells. The primary antibodies included polyclonal antibodies against phospho-YAP (Ser127) (p-YAP, 1:1000, bsm-52214R, Bioss, China), YAP (1:1000, #14074, Cell Signaling Technology, United States), α-SMA (1:10000, ab124964, Abcam, United States), collagen Ⅰ (1:750, AF7001, Affinity, United States), and GAPDH (1:1000, GB12002, Servicebio Technology, China). The primary proteins were detected by using horseradish peroxidase-conjugated secondary antibodies (Abcam, United States), and the immune complexes were finally developed by using the enhanced chemiluminescence Plus kit (Amersham, Freiburg, Germany).

### ROS detection

The intracellular ROS levels were detected using the DCFA-DA fluorescent probe (S0033S, Beyotime Biotechnology, Shanghai, China). Renal fibroblasts were seeded on laser confocal petri dishes lined with PA gels and cultured at 37°C for 24 h with 5% CO_2_ (with PD or without PD). Dilute DCFH-DA with the serum-free medium at the ratio of 1:1000 to 10 μ mol/L, remove the cell culture medium, and add the diluted DCFH-DA that can cover the cells. Incubate in the cell incubator for 20–30 min, and then wash with serum-free medium 3 times to remove DCFH-DA that has not entered the cells. All procedures are carried out in the dark, and samples are observed by CLSM.

Dihydroethidium (DHE) fluorescence was used to detect the level of ROS in the kidney tissues. The kidney tissue mounted on polylysine-coated glass slides was incubated in 10 *μ*M DHE for 30 min at 37°C in a humidified chamber protected from light, then incubated with DAPI solution at room temperature for 10 min, and kept in the dark. After washing with PBS, the sections were mounted and observed by CLSM. In the presence of superoxide anion, DHE is oxidized to ethidium, which produces red fluorescence.

### Statistical analysis

The data were presented as the mean ±standard deviation (SD). Using SPSS statistical software package (SPSS, Inc., Chicago, IL, United States), Students’ *t*-test and one-way analysis of variance (ANOVA) followed by Dunnett’s multiple comparison tests were used to compare the differences between the data means, and *p* < 0.05 was considered to be statistically significant.

## Results

### The kidney stiffness of DKD rats increased with the aggravation of renal fibrosis

**FIGURE 1:**
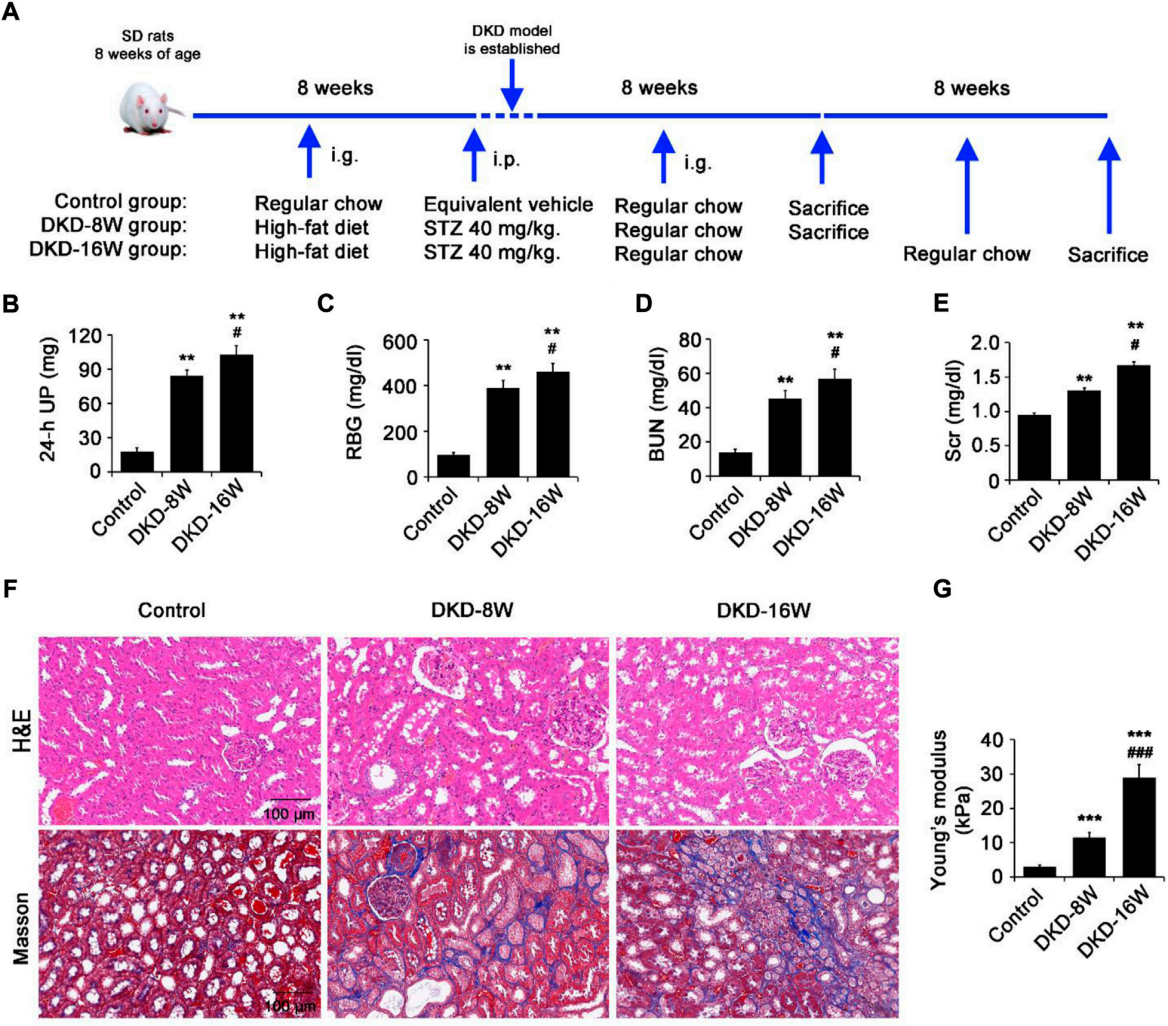
The kidney stiffness of DKD rats increased with the aggravation of renal fibrosis. **(A)** SD rats were fed with a high-fat diet for 8 weeks, and then injected with 40 mg/kg STZ intraperitoneally to establish the DKD rat model. **(B–E)** The 24 h-UP **(B)**, RBG **(C)**, BUN **(D)** and Scr **(E)** were measured at the 8th (DKD-8W) and 16th (DKD-16W) weeks after successful DKD modeling. **(F)** Histopathology analysis of the kidneys in DKD-8W and DKD-16W rats was performed by hematoxylin-eosin (H&E) staining and masson staining. **(G)** Using a cantilever-based nanointender, the Young’s Modulus of cryostat sections of the kidneys in normal, DKD-8W and DKD-16W rats were assessed. STZ, streptozocin; UP, urine protein; RBG, random blood glucose; BUN: blood urea nitrogen; Scr: serum creatinine; DKD, diabetic kidney disease; DKD-8W and DKD-16W, 8 and 16 weeks after the establishment of DKD. *p < 0.05, **p < 0.01, ***p < 0.001 vs control; ^#^ *p* < 0.05, ^##^ *p* < 0.01, ^###^ *p* < 0.001 vs DKD-8W (n = 6–8).

In the kidneys of DKD rats, the correlation between the degree of fibrosis and the true stiffness has not yet been fully determined. In this study, at the 8th (DKD-8W) and 16th (DKD-16W) weeks after successful DKD modeling, the 24-h UP, RBG, BUN, Scr and renal tissue histopathology were evaluated, and the stiffness of the renal cortex was measured by nanoindentation (Figure 1A).

The 24-h UP (Figure 1B), RBG (Figure 1C), BUN (Figure 1D) and Scr (Figure 1E) of DKD-8W and DKD-16W rats were significantly higher compared with that of control rats. Compared with DKD-8W rats, DKD-16W rats showed higher RBG, 24-h UP, BUN and Scr (Figures 1B–E). Masson staining showed that compared with that in control rats, a small amount and a large amount of blue-stained collagen fibers increased in the peritubular interstitial region in DKD-8W and DKD-16W rats, respectively (Figure 1F). The above indicators suggest that DKD-8W and DKD-16W represent different stages of renal fibrosis in DKD rats.

Then, we further evaluated whether there were differences in the stiffness of the renal cortex of DKD rats in different fibrosis stages. The results of nanoindentation showed that the elastic modulus of kidneys in DKD-8W (E = 11.47±1.45 kPa) and DKD-16W (E = 28.9±3.81 kPa) rats were significantly higher than that of control kidneys (E = 2.94±0.52 kPa; all *p* < 0.001), and that of DKD-16W kidneys was the highest (DKD-16W vs. DKD-8W, *p* < 0.001) (Figure 1G), suggesting that with the development of DKD, the increase of kidney stiffness was consistent with the aggravation of renal fibrosis.

The stiffness gradient of PA gels *in vitro*, which simulates the stiffness of fibrotic kidney *in vivo*, changes the intracellular tension of renal fibroblasts.

**FIGURE 2:**
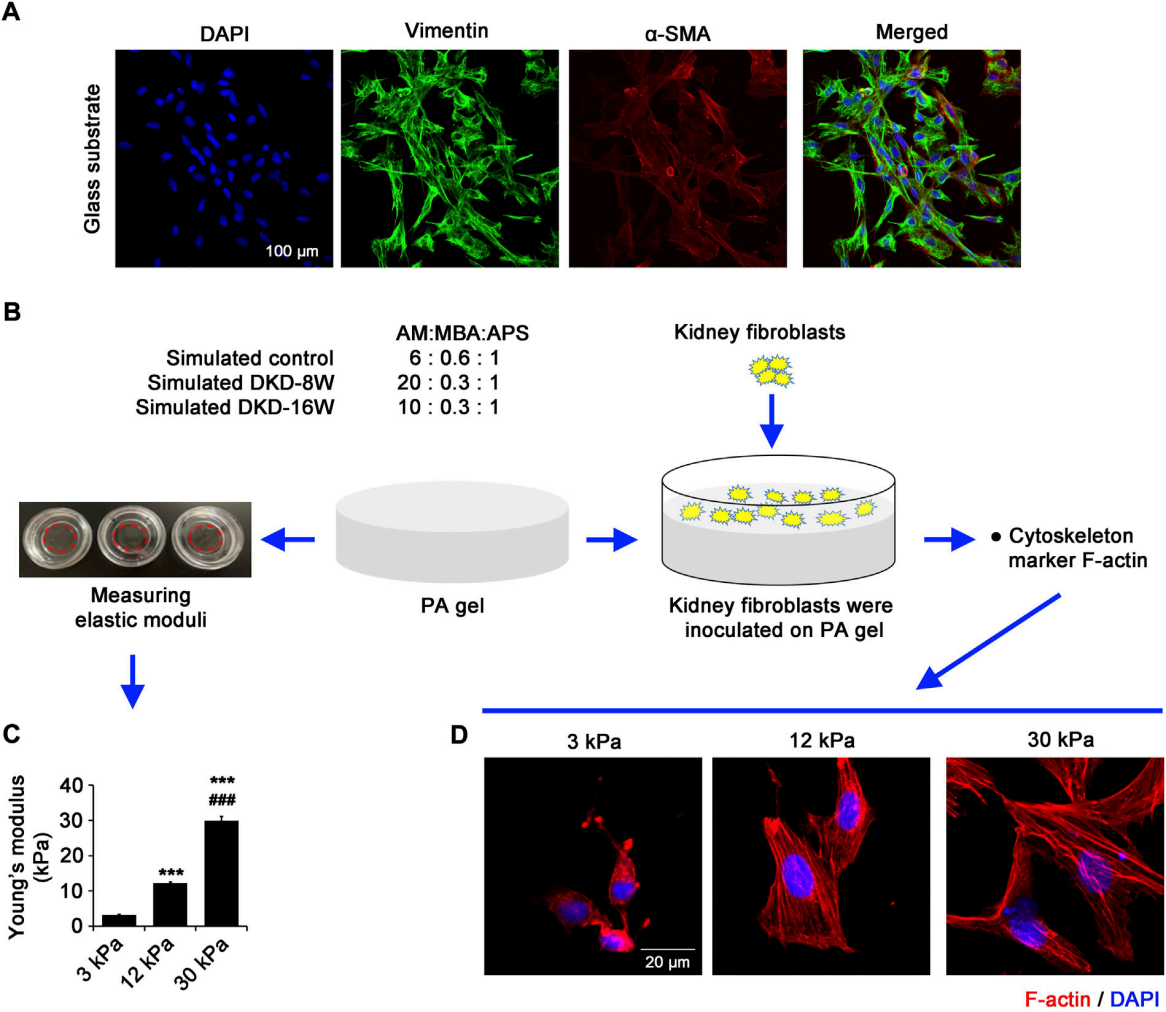
The stiffness gradient of PA gels *in vitro*, which simulates the stiffness of fibrotic kidney *in vivo*, changes the intracellular tension of renal fibroblasts. **(A)** Fibroblasts isolated from SD rat kidneys were seeded in the glass dish, and confocal laser scanning microscopy (CLSM) was performed with α-SMA and vimentin. **(B)** *In vitro* PA gel stiffness gradient system was prepared by adjusting the ratio of AM and MBA. **(C)** The Young’s moduli of 3 kPa, 12 kPa and 30 kPa were determined by the nanoindentation technique to simulate the extracellular matrix (ECM) environment in the kidneys of normal and DKD rats, respectively. **(D)** CLSM shows F-actin in the renal fibroblasts cultured on PA gels with different stiffness (3 kPa, 12 kPa and 30 kPa) for 24 h α-SMA, alpha-smooth muscle actin; DAPI, 4',6-diamidino-2-phenylindole; AM, Acrylamide; MBA, N,N'-methylenebisacrylamide; APS, ammonium persulfate; PA gel, polyacrylamide hydrogel. *p < 0.05, **p < 0.01, ***p < 0.001 vs 3 kPa; ^#^ *p* < 0.05, ^##^ *p* < 0.01, ^###^ *p* < 0.001 vs 12 kPa (n = 6).

The cells isolated from rat kidneys were cultured in glass dishes (hard matrix environment, ∼10^6^ kPa). Immunofluorescence showed that the fibroblast marker vimentin and myofibroblast marker α-SMA were highly expressed and co-localized, suggesting that rat fibroblasts were successfully obtained in this study (Figure 2A). In order to simulate the kidney stiffness of DKD rats, PA gels with different stiffness were constructed by adjusting the ratio of AM and MBA (Figure 2B). The elastic moduli of PA gels prepared by polymerization of AM and MBA in the proportions of 6:0.6, 20:0.3 and 10:0.3 are 3.19±0.18 kPa (regarded as ∼3 kPa), 12.26±0.35 kPa (∼12 kPa) and 29.95±1.21 kPa (∼30 kPa), which could *in vitro* mimic that of kidneys in normal, DKD-8W and DKD-16W rats, respectively (Figure 2C).

To evaluate the intracellular tension on substrates with different rigidity, the rat kidney fibroblasts were inoculated on PA gels with different stiffness and cultured for 24 h using immunofluorescence to observe the changes of cytoskeleton marker F-actin. On the soft substrate of 3 kPa, the fibroblasts are small, and have aggregates of F-actin at the cell periphery without defined structures (Figure 2D). On 12 kPa gels, the spreading area of fibroblasts increased, and stress fibers gradually formed, but the fibers were short and slender, and mainly distributed at the edge of cells (Figure 2D). Cells on 30 kPa gels spread much more obviously than cells on 12 kPa gels, forming a cross-linked reticular fiber bundle (Figure 2D). These data show that we have successfully constructed PA gel systems with different stiffness gradients, which have definite effects on the cell behavior of fibroblasts.

### Polydatin inhibits pathological matrix stiffness-induced YAP expression in renal fibroblasts

**FIGURE 3:**
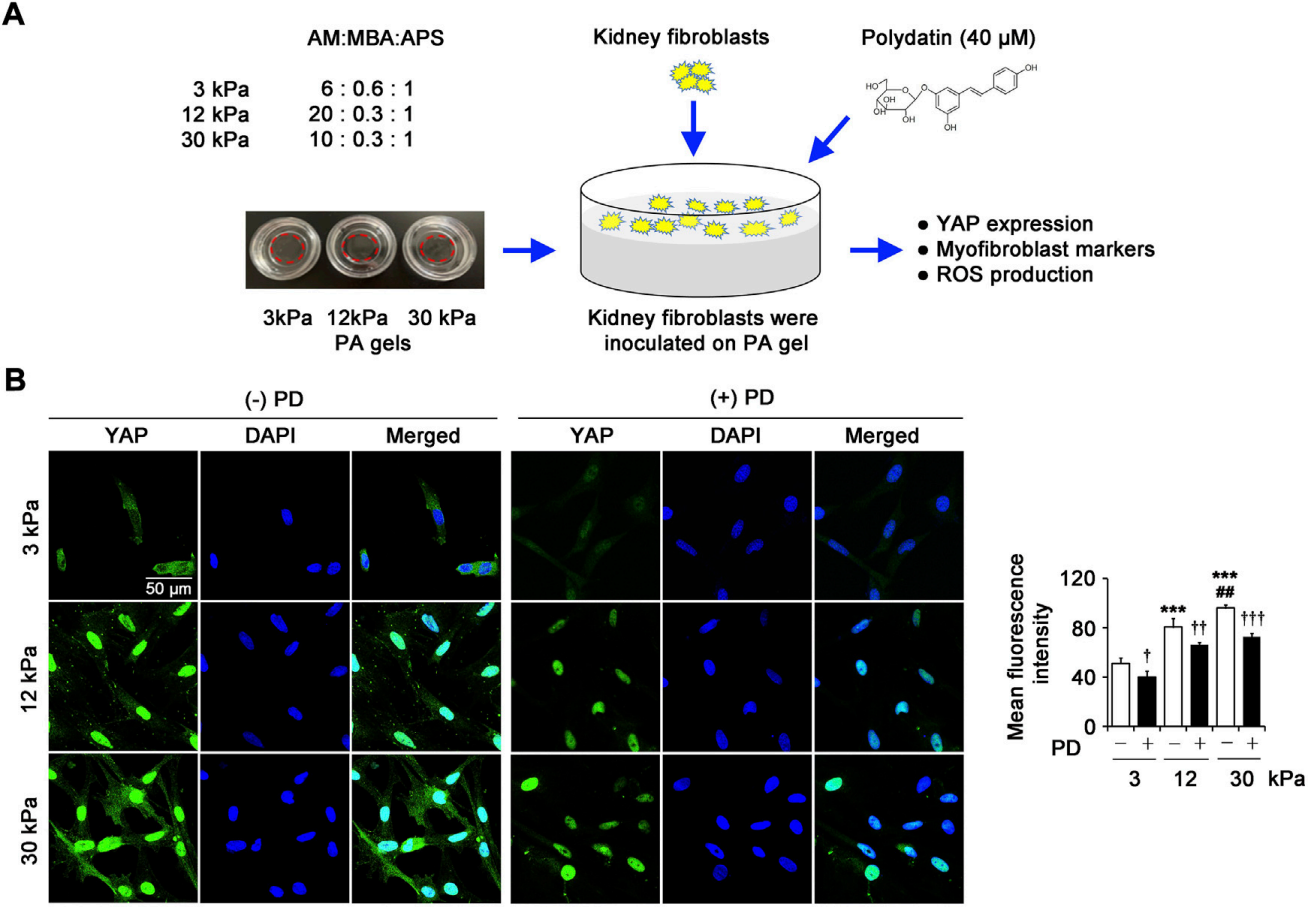
PD inhibits pathological matrix stiffness-induced YAP expression in renal fibroblasts. **(A)** The schedule of exploring the effect of PD on renal fibroblasts *in vitro* on PA gels with different matrix stiffness. **(B)** Confocal laser scanning microscopy (CLSM) shows YAP in the renal fibroblasts cultured on PA gels with different stiffness (3 kPa, 12 kPa and 30 kPa) for 24 h. Treatment with PD (40 μM) results in a dramatically reduced levels of YAP. AM, Acrylamide; MBA, N,N'-methylenebisacrylamide; APS, ammonium persulfate; PA gel, polyacrylamide hydrogel; PD, polydatin; YAP, Yes-associated protein; DAPI, 4',6-diamidino-2-phenylindole; ROS, reactive oxygen species. *p < 0.05, **p < 0.01, ***p < 0.001 vs 3 kPa; ^#^ *p* < 0.05, ^##^ *p* < 0.01, ^###^ *p* < 0.001 vs 12 kPa; ^†^ *p* < 0.05, ^††^ *p* < 0.01, ^†††^ *p* < 0.001 vs. (-) PD (n = 6).

The schedule of exploring the effect of PD on renal fibroblasts *in vitro* on PA gels with different matrix stiffness was outlined (Figure 3A). YAP is the downstream effector of the Hippo pathway, which has been proved to be a critical driver of organ fibrosis. In this study, the immunostaining of YAP in renal fibroblasts showed low and diffuse staining in cells grown on 3 kPa PA gels and predominantly nuclear staining in cells grown on 12 kPa and 30 kPa PA gels, and the YAP protein expression in cells on 30 kPa PA gels was higher than that on 12 kPa PA gels (Figure 3B). However, PD (40 μM) significantly inhibited the increased YAP expression induced by pathological matrix stiffness (Figure 3B), suggesting the role of PD in YAP-related mechano-transduction in renal fibroblasts.

### Polydatin attenuates pathological matrix stiffness-induced renal fibroblast activation

**FIGURE 4:**
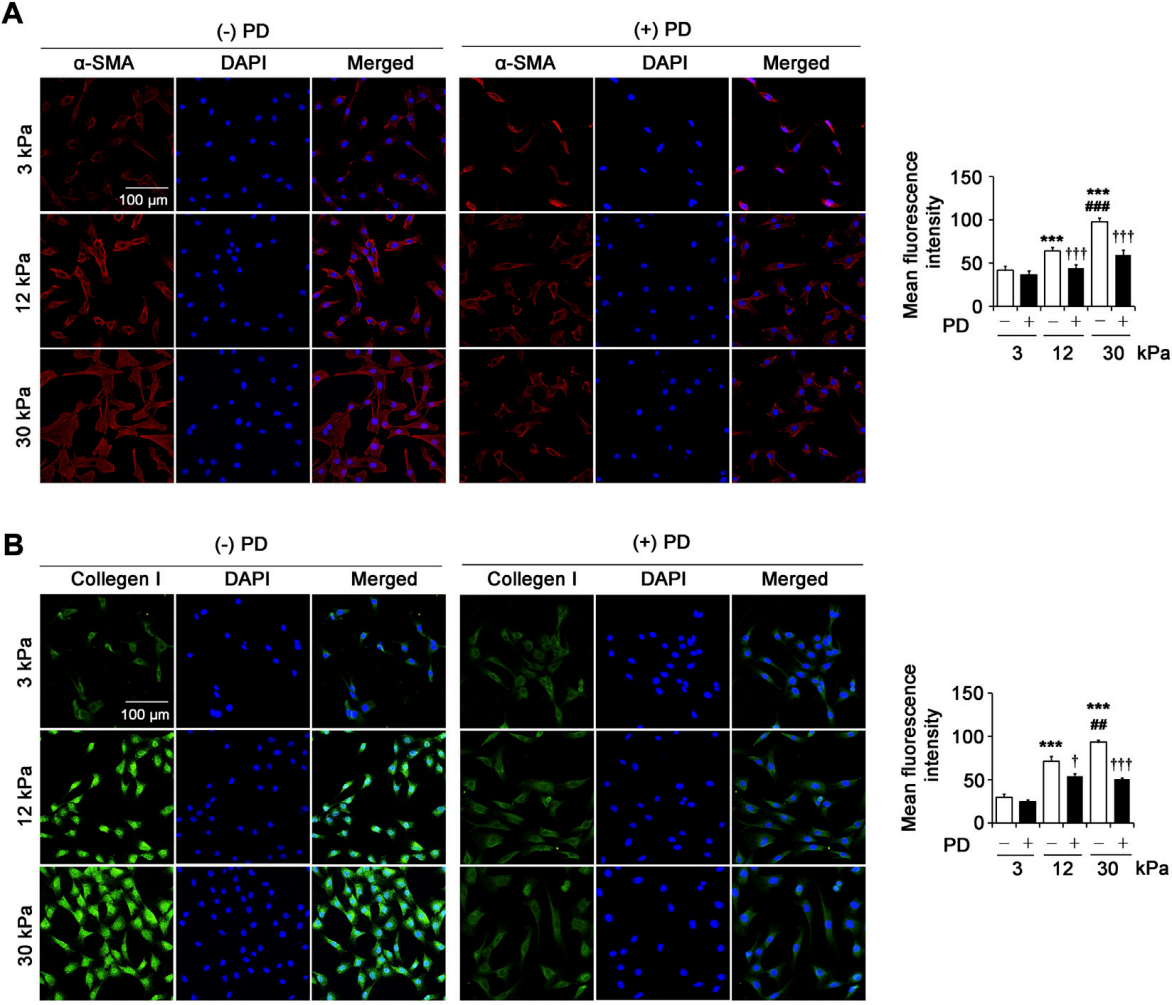
PD attenuates pathological matrix stiffness-induced renal fibroblast activation. **(A)** Confocal laser scanning microscopy (CLSM) shows α-SMA in the renal fibroblasts cultured on PA gels with different stiffness (3 kPa, 12 kPa and 30 kPa) for 24 h. PD (40 μM) treatment results in the decrease of α-SMA expression. **(B)** CLSM shows collagen I in the renal fibroblasts cultured on PA gels with different stiffness (3 kPa, 12 kPa and 30 kPa) for 24 h. Treatment with PD (40 μM) results in a decrease in the expression of collagen I. PD, polydatin; α-SMA, alpha-smooth muscle actin; DAPI, 4',6-diamidino-2-phenylindole. *p < 0.05, **p < 0.01, ***p < 0.001 vs 3 kPa; ^#^ *p* < 0.05, ^##^ *p* < 0.01, ^###^ *p* < 0.001 vs 12 kPa; ^†^ *p* < 0.05, ^††^ *p* < 0.01, ^†††^ *p* < 0.001 vs. (-) PD (n = 6).

Increased activation of renal fibroblasts is the key to the initiation and maintenance of unremitting deposition of ECM in renal fibrosis. In the pathological environment of fibrosis, renal fibroblasts can differentiate into myofibroblasts, which express α-SMA, synthesize and produce ECM such as collagen I. The immunofluorescence results showed that the expression of α-SMA (Figure 4A) and collagen I (Figure 4B) in fibroblasts on 12 kPa and 30 kPa PA gels increased significantly compared with that on 3 kPa PA gels, and the highest expression of α-SMA and collagen I was found in cells on 30 kPa PA gels. Importantly, PD (40 μM) significantly reversed the expression of α-SMA and collagen I induced by 12 kPa and 30 kPa stiffness (Figures 4A,B), suggesting that PD inhibited the activation of fibroblasts and the synthesis of ECM induced by pathological matrix stiffness.

### Polydatin is similar to VP in inhibiting the expression and nuclear translocation of YAP in kidneys of DKD rats

**FIGURE 5:**
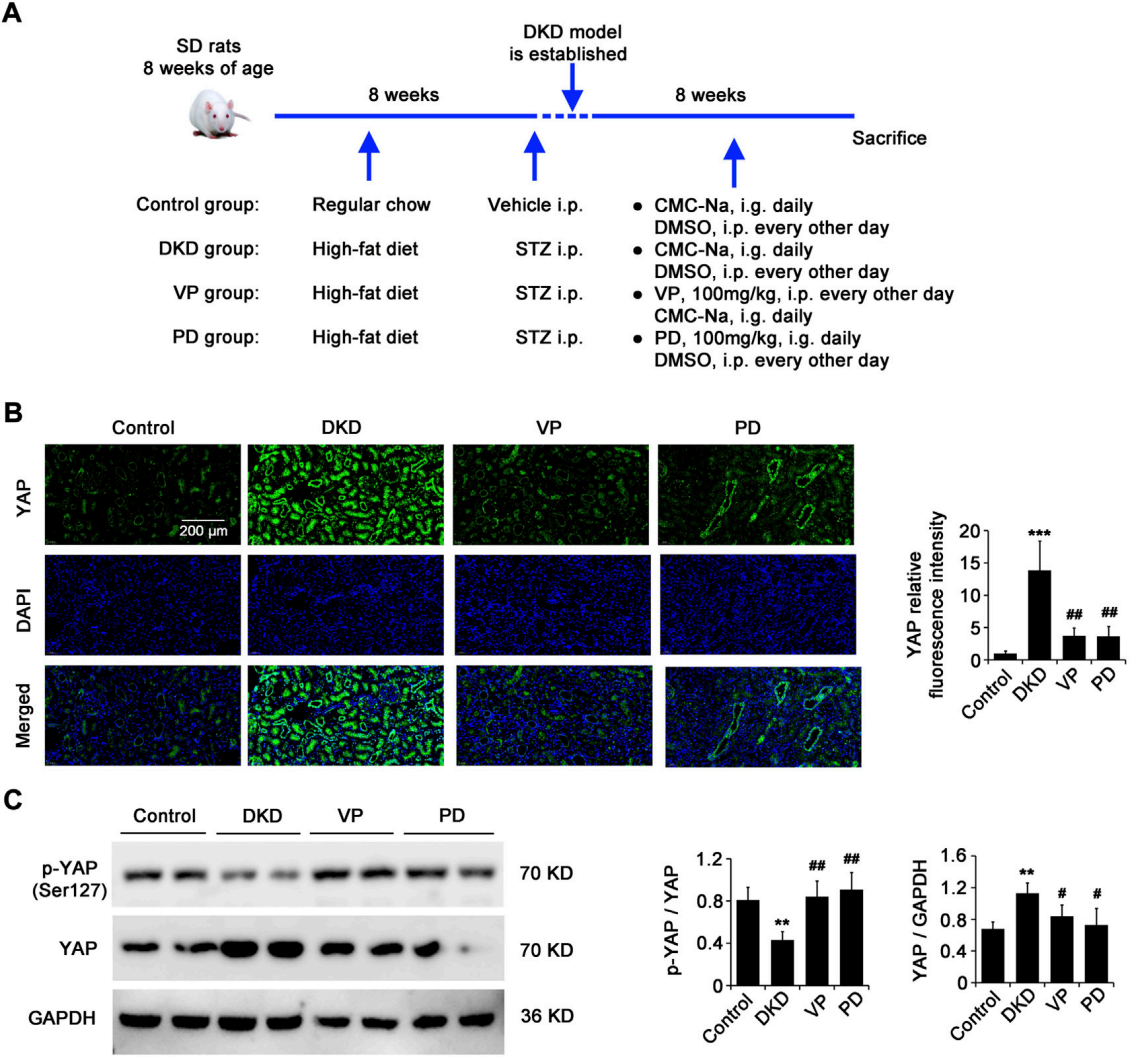
PD is similar to VP in inhibiting the expression and nuclear translocation of YAP in kidneys of DN rats. **(A)** Immediately after modeling, DKD rats were treated with PD (100 mg/kg, i.e. daily) or VP (a pharmacological inhibitor of YAP, 100 mg/kg, I. p. every other day) for 8 weeks. Age-matched healthy rats and DKD rats treated with equivalent vehicle were used as negative control (control group) and model control (DKD group) respectively. **(B,C)** The renal expression and nuclear translocation of YAP in DKD rats were captured by CLSM **(B)** and western blot **(C)**. DKD, diabetic kidney disease; VP, verteporfin; PD, polydatin; STZ, streptozocin; CMC-Na, sodium carboxymethylcellulose; DMSO, dimethyl sulfoxide; YAP, Yes-associated protein; p-YAP, phospho-YAP; DAPI, 4',6-diamidino-2-phenylindole; α-SMA, alpha-smooth muscle actin; DAPI, 4',6-diamidino-2-phenylindole. *p < 0.05, **p < 0.01, ***p < 0.001 vs control; ^#^ *p* < 0.05, ^##^ *p* < 0.01, ^###^ *p* < 0.001 vs DKD; ^†^ *p* < 0.05, ^††^ *p* < 0.01, ^†††^ *p* < 0.001 vs VP (n = 6–8).

The inhibitory effect of PD on YAP expression in renal fibroblasts induced by pathological matrix stiffness *in vitro* inspires us to further explore whether the therapeutic effect of PD in DKD *in vivo* model is related to the regulation of YAP. For this purpose, the DKD rats were treated with PD (100 mg/kg, i. g. daily) or VP (a pharmacological inhibitor of YAP, 100 mg/kg, i. p. every other day) for 8 weeks immediately after modeling, with age-matched healthy and DKD rats treated with the equivalent vehicle as negative control (Control group) and model control (DKD group), respectively (Figure 5A). CLSM results showed that the expression of YAP in kidneys of DKD rats was significantly higher than that of the control group, but it was significantly decreased by PD or VP treatment (Figure 5B). It is well known that in normal cells, YAP is located in the cytoplasm in its inactivated phosphorylated form. When YAP is activated, it transfers to the nucleus and upregulates its target genes, which are responsible for ECM remodeling. The WB results showed that the expression of YAP in kidneys of DKD rats was significantly higher than that of the control group, and the ratio of p-YAP/YAP was significantly lower (Figure 5C), suggesting that there was an increase of YAP activation in kidneys of DKD rats. Importantly, PD or VP reversed the activation of YAP in the kidneys of DKD rats, characterized by a decrease in YAP expression and an increase in p-YAP/YAP (Figure 5C), indicating that PD had a similar effect to VP in reducing YAP expression and nuclear translocation.

### Polydatin is superior to VP in alleviating renal injury and fibrosis in DKD rats

**FIGURE 6:**
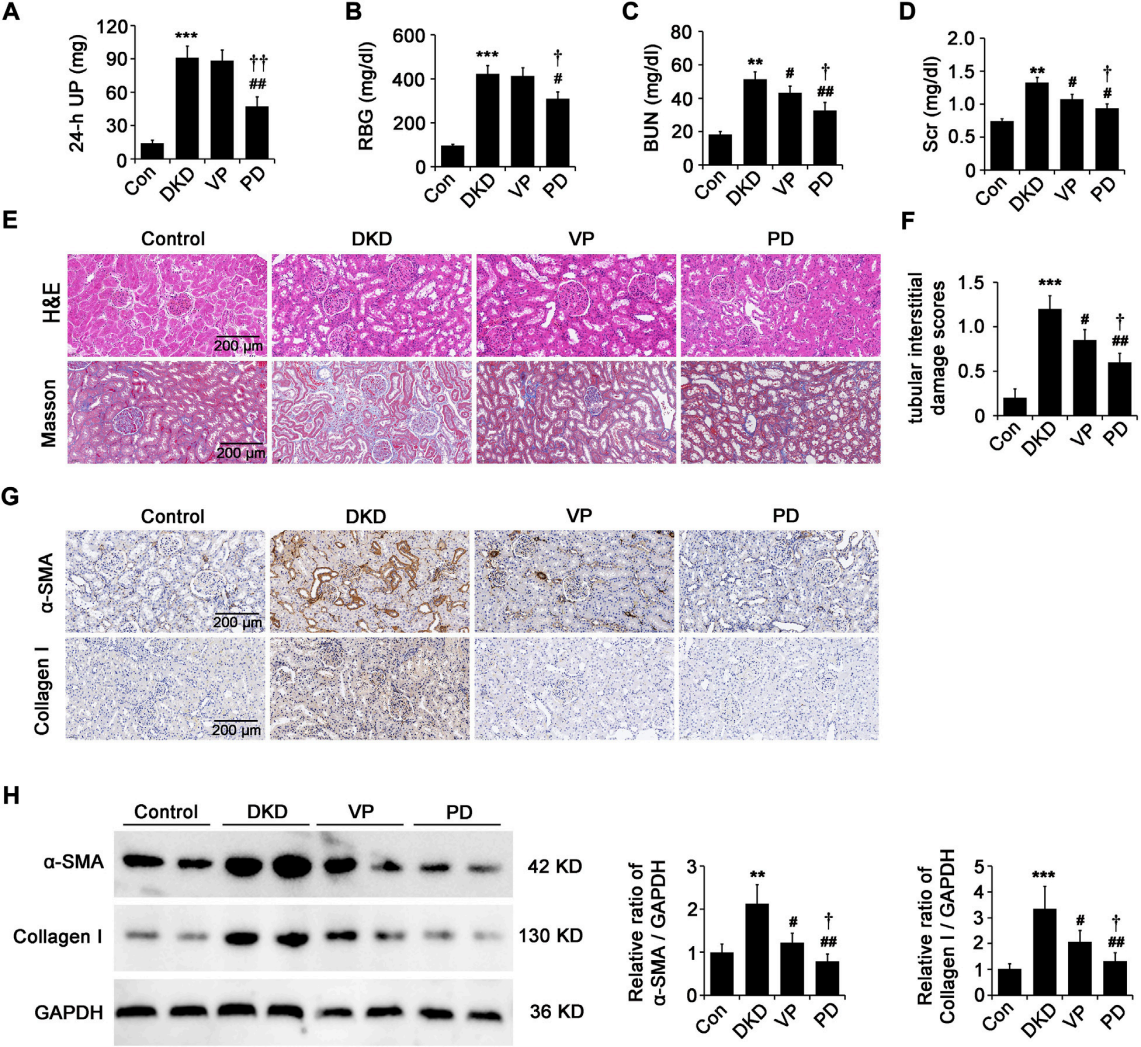
PD is superior to VP in alleviating renal injury and fibrosis in DKD rats. **(A–D)** The 24 h-UP **(A)**, RBG **(B)**, BUN **(C)** and Scr **(D)** were measured at 8 weeks after successful DKD modeling. **(E)** Histopathology analysis of the kidneys was performed by hematoxylin-eosin (H&E) staining and masson staining. **(F)** Tubular injury as assessed by tubular interstitial damage scores. **(G)** Immunohistochemical staining of α-SMA and collagen I in renal paraffin tissue of different groups. **(H)** Western blot analysis of α-SMA and collagen I in different groups. UP, urine protein; RBG, random blood glucose; BUN: blood urea nitrogen; Scr: serum creatinine; DKD, diabetic kidney disease; VP, verteporfin; PD, polydatin; α-SMA, alpha-smooth muscle actin. *p < 0.05, **p < 0.01, ***p < 0.001 vs control (Con); ^#^ *p* < 0.05, ^##^ *p* < 0.01, ^###^ *p* < 0.001 vs DKD; ^†^ *p* < 0.05, ^††^ *p* < 0.01, ^†††^ *p* < 0.001 vs VP (n = 6–8).

We further evaluated the effects of PD (100 mg/kg, i. g. daily) or VP (100 mg/kg, i. p. every other day) on renal injury and fibrosis in DKD rats. Compared with that of DKD rats treated with the equivalent vehicle, the levels of 24 h-UP (Figure 6A), RBG (Figure 6B), BUN (Figure 6C) and Scr (Figure 6D) in DKD rats treated with PD were significantly lower, although they did not reach the levels of healthy control rats. Compared with DKD rats treated with the equivalent vehicle, the levels of BUN and Scr in DKD rats treated with VP decreased, but the levels of 24 h-UP and RBG did not change (Figures 6A–D). Importantly, the effect of VP on improving renal function of DKD rats is weaker than that of PD (Figures 6A–D). An increase of glomerular matrix, tubular dilatation and nuclear shedding shown by HE staining, and the deposition of blue-stained collagen fibers in the peritubular interstitial region shown by masson staining were observed in the kidneys of DKD rats compared to the healthy control (Figure 6E), and this pathological kidney injury was obviously relieved by PD or VP, and PD had the best effect, as indicated by tubular interstitial damage scores (Figure 6F). We further explored the effects of PD or VP on the expression of ECM proteins, such as α-SMA and collagen I. As shown in Figures 6G,H, control group kidneys only expressed a little amount of these ECM proteins. However, these proteins were dramatically increased in the kidneys of DKD-8W rats (Figures 6G,H). Furthermore, treatment with PD or VP inhibited the expression of ECM proteins in the kidneys of DKD-8W rats, and the effect of PD was better (Figures 6G,H). These results suggest that PD has a stronger effect than VP in alleviating kidney injury and fibrosis in DKD rats.

### Polydatin limits the increase of intracellular ROS *in vitro* and *in vivo*

**FIGURE 7:**
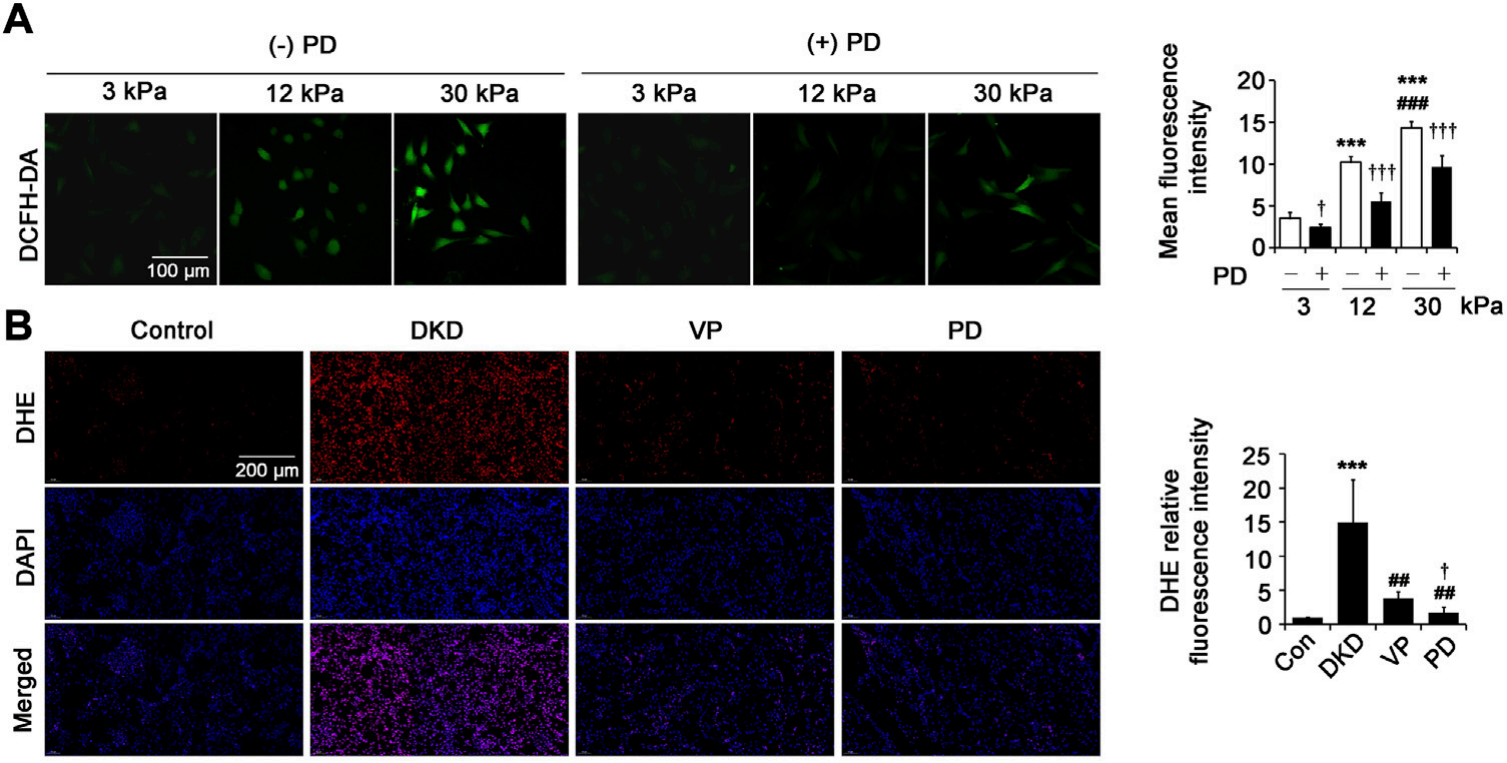
PD limits the increase of intracellular ROS *in vitro* and *in vivo*. **(A)** Immunofluorescence staining shows ROS level (DCFH-DA) in the renal fibroblasts cultured on PA gels with different stiffness (3 kPa, 12 kPa and 30 kPa) for 24 h *p < 0.05, **p < 0.01, ***p < 0.001 vs 3 kPa; ^#^ *p* < 0.05, ^##^ *p* < 0.01, ^###^ *p* < 0.001 vs 12 kPa; ^†^ *p* < 0.05, ^††^ *p* < 0.01, ^†††^ *p* < 0.001 vs. (-) PD (n = 6). **(B)** The ROS level of kidney tissues was captured by CLSM at 8 weeks after successful DKD modeling (DHE fluorescent staining). *p < 0.05, **p < 0.01, ***p < 0.001 vs control (Con); ^#^ *p* < 0.05, ^##^ *p* < 0.01, ^###^ *p* < 0.001 vs DKD; ^†^ *p* < 0.05, ^††^ *p* < 0.01, ^†††^ *p* < 0.001 vs VP (n = 6–8). PD: polydatin; DCFH-DA: 2′,7′-dichlorofluorescin diacetate; DKD, diabetic kidney disease; VP, verteporfin; DHE: dihydroethidium; DAPI, 4',6-diamidino-2-phenylindole.

Reactive oxygen species (ROS) overproduction is one of the important mechanisms of the development of tubulointerstitial injury and fibrosis in DKD. In this study, intracellular ROS were measured by using DCFH-DA fluorescent probes for the fibroblasts cultured with different matrix stiffness (3 kPa, 12 kPa and 30 kPa) and DHE fluorescent probes for the kidneys of DKD rats. First, we evaluated the effect of PD on pathological matrix stiffness-induced ROS levels in renal fibroblasts. Compared with the cells on 3 kPa PA gels, the ROS levels increased significantly in fibroblasts on 12 kPa and 30 kPa PA gels, with the highest level in the cells on 30 kPa PA gels (Figure 7A). This effect was reduced by supplementing with a 40 μM dose of PD (Figure 7A), indicating that PD may play a role in inhibiting ROS overproduction in fibroblasts induced by pathological matrix stiffness. Likewise, the level of intracellular ROS in kidneys measured by the fluorescent probe DHE was increased in DKD rats compared with that of the control and was ameliorated in the DKD rats treated with PD, and the effect was better than that of VP (Figure 7B).

## Discussion

Regardless of the etiology of chronic kidney disease (CKD), renal fibrosis characterized by excessive deposition of ECM in the kidney leads to increased stiffness of renal tissue with the aggravation of CKD (Cruz et al., 2021; Fu et al., 2021; Rayego-Mateos et al., 2021; Zhao et al., 2022). Fibroblasts maintain the metabolic balance of ECM under normal physiological conditions, and can be activated in renal fibrosis to produce large amounts of ECM (Huang et al., 2020). This study evaluated the role of PD in regulating the mechano-transduction pathway of renal fibroblasts, with the following highlights: 1) For the first time, based on real kidney stiffness in different stages of renal fibrosis in DKD rats, we prepared a PA gel system which can simulate the real ECM stiffness environment of kidney tissue *in vivo* and confirmed that pathological matrix stiffness promoted the expression of α-SMA and collagen I, YAP nuclear translocation and ROS production in fibroblasts; 2) For the first time, using the *in vitro* model of renal fibroblasts cultured in PA hydrogels with different stiffness and the *in vivo* model of DKD rats intervened by PD or VP (a pharmacological inhibitor of YAP), we proved that PD can delay fibrosis progression via a mechanism that is dependent, at least in part, on inhibiting YAP expression and nuclear translocation.

Magnetic resonance elastography (MRE) and ultrasound shear wave elastography (SWE) have emerged as preferred non-invasive techniques for the clinical assessment of the fibrotic tissue stiffness, but the confounding factors owing to the complex architecture and mechanical properties of the kidney, such as renal anisotropy, tissue viscoelasticity and renal hemodynamics, influence the reliability and accuracy of the measurement results obtained (Lim et al., 2021; Seyedpour et al., 2021). Nanoindentation technology and atomic force microscopy (AFM) have high spatial resolution and mechanical sensitivity, which have been widely used to measure the mechanical properties of biomaterials and tissues (Wu et al., 2020). The stiffness of normal tissues ranges from hundreds of Pa (very soft, such as fat and brain) to greater than 1000 kPa (very stiff, such as cartilage and bone), and the variance in stiffness for individual tissue types is usually small, within 10–15% of the average (Miller and Janmey, 2015). It has been reported that the elastic modulus of normal rat glomerulus measured by AFM is 2.2–2.5 kPa (Wyss et al., 2011; Embry et al., 2016; Embry et al., 2018), but the stiffness of renal tissue with abnormal fibrosis has not been reported so far.

Diabetic kidney disease (DKD) is the main cause of end-stage renal disease globally (Nomura et al., 2021). It is a complex disease process and involves many pathophysiologic mechanisms such as oxidative stress, inflammation and renal tubular injury caused by hyperglycemia, which eventually leads to renal fibrosis (Chen et al., 2022; Jung and Yoo, 2022). In this study, we first measured the stiffness of kidney tissues of DKD rats at different fibrosis stages (normal, 8 and 16 weeks after DKD), then successfully made an *in vitro* PA gel system consistent with the stiffness of kidneys in DKD rats *in vivo* by adjusting the ratio of AM and MBA, and finally determined the stiffness of 3 kPa, 12 kPa and 30 kPa to simulate the kidney stiffness in normal, DKD-8W and DKD-16W rats respectively.

The actin cytoskeleton, an important element for cells to feel basal stiffness, plays a major role in determining the tension state of cells, and the high intracellular tensional environment characteristic of myofibroblasts is the appearance of actin stress fibers (Rayego-Mateos et al., 2021). In this study, the stiffness gradient of PA gels changed the intracellular tension of fibroblasts, which showed that 12 kPa PA gels led to F-actin stress fibers in fibroblasts, while 30 kPa PA gels led to more obvious reticular fiber bundles in fibroblasts, suggesting that the PA gel gradient system prepared by us can simulate the real ECM stiffness environment of the DKD rat kidney.

Excessive accumulation of ECM leads to the increase of matrix stiffness, which is mutually causal with fibrosis. Mechanical force can send signals to cells through mechano-transduction to control cell proliferation, differentiation and death, including the activation of fibroblasts, which is manifested by increased secretion of α-SMA and Collagen I (Romani et al., 2021). In the Hippo signaling pathway, the transcription factor YAP is essential for mediating the biological response to mechanical signals at cell-ECM adhesions (Panciera et al., 2017). Increased ECM stiffness stimulates YAP nuclear localization, which leads to ECM deposition and, in turn, contributes to ECM stiffness increase (Cai et al., 2021; Fu et al., 2021). In this study, compared with 3 kPa PA gel (representing normal tissue stiffness), the stiffness of 12 kPa PA gel induced the increased expression of α-SMA, collagen I and YAP in fibroblasts, while the stiffness of 30 kPa PA gel further aggravated the expression of the above-mentioned proteins, suggesting the correlation between fibroblast activation and the YAP pathway mediated by different degrees of pathological matrix stiffness. Our results are consistent with the recently reported studies (Szeto et al., 2016; Liang et al., 2017; Rinschen et al., 2017; Fu et al., 2021) on the role of matrix stiffness-mediated activation of YAP-related pathways in promoting renal fibrosis. The difference is that the stiffness of PA gels prepared by us is based on the stiffness of normal kidney and fibrotic kidney to simulate the *in vivo* growth microenvironment of cells, while most other studies use 50 kPa (Liang et al., 2017; Fu et al., 2021) and 100 kPa (Szeto et al., 2016) as stiff matrix standards, which far exceeds the measured real stiffness (12 and 30 kPa) of the fibrotic kidney in DKD rats.

In recent years, the role of PD in protecting acute renal injury (AKI) has attracted much attention from scholars, including our previous studies which confirmed its protective effects on anti-apoptosis, anti-inflammation, anti-oxidative stress and anti-ferroptosis in ischemia/reperfusion-induced AKI and cisplatin-induced AKI mouse models, respectively (Liu et al., 2015; Meng et al., 2015; Meng et al., 2016; Zhou et al., 2022). Several studies have confirmed its anti-fibrosis effect by regulating the Cx32-Nox4 signaling pathway (Chen et al., 2020a) and Nrf2-ARE anti-oxidative pathway (Huang et al., 2015; Gong et al., 2017) in DKD models. PD has also been proved to have protective effects on hepatic fibrosis (Zhao et al., 2017; Li et al., 2018; Lan et al., 2020; Liu et al., 2020; Zhao et al., 2020; Tang et al., 2022), pulmonary fibrosis (Cao et al., 2017; Tang et al., 2019; Zeng et al., 2019; Ali et al., 2022) and myocardial fibrosis (Tan et al., 2020). Resveratrol, an aglycone of PD, has recently been proved to inhibit the activation of hepatic stellate cells by activating Hippo pathway-mediated YAP inhibition, thus playing an anti-fibrosis role (Li et al., 2021). Although YAP signaling is closely associated with the progression of renal fibrosis, little is known the effect of PD on YAP regulation. Based on the known anti-fibrosis effect of PD through regulating biochemical signals, this study intends to further evaluate the role of PD in regulating mechanosensitive YAP-dependent pathways in the renal fibroblast activation induced by matrix stiffness. As expected, we preliminarily confirmed the inhibitory effect of PD on fibroblast activation, YAP expression and ROS production activated by PA gels with different stiffness gradients, suggesting the potential role of PD in anti-fibrosis by regulating the mechano-transduction pathway. Importantly, PD showed no inferior effect to VP in inhibiting the expression and nuclear translocation of YAP in kidneys of DKD rats, suggesting that the anti-fibrosis effect of PD was at least partly through regulating YAP. However, the mechanism underlying the direct regulation of YAP by PD remains to be explored. We hypothesize that the activation of Hippo pathway may be related to the effect of PD treatment on renal fibrosis, although it has not been reported yet.

## Conclusion

PD has been clinically used in the treatment of patients with interstitial cystitis/bladder pain syndrome (Cervigni et al., 2019), irritable bowel syndrome (Cremon et al., 2017) and chronic alcoholism (Pace et al., 2015). Surprisingly, PD has recently been proposed as a potential natural active product with a therapeutic effect on Coronavirus Disease 2019 (De Angelis et al., 2021; Doustimotlagh and Eftekhari, 2021; Perrella et al., 2021; Xu et al., 2021; Lo Muzio et al., 2022; Sun et al., 2022). Although this study only confirmed the potential role of PD in inhibiting pathological matrix stiffness induced activation of renal fibroblasts by regulating YAP-related mechano-transduction pathways *in vitro*, and the correlation between the anti-fibrosis effect of PD and the inhibition of YAP activation in DKD model *in vivo*, our research results are expected to open up a new perspective for the mechanism of PD in the clinical treatment of renal fibrosis.

## Data Availability

The original contributions presented in the study are included in the article/Supplementary Material, further inquiries can be directed to the corresponding authors (mail: xjsnlhb@fmmu.edu.cn).
