# Supplementary figures and images for: Polydatin attenuates tubulointerstitial fibrosis in diabetic kidney disease by inhibiting YAP expression and nuclear translocation
Source: Front Physiol. 2022 Oct 7;13:927794. doi: 10.3389/fphys.2022.927794 (PMC9585250; doi:10.3389/fphys.2022.927794)

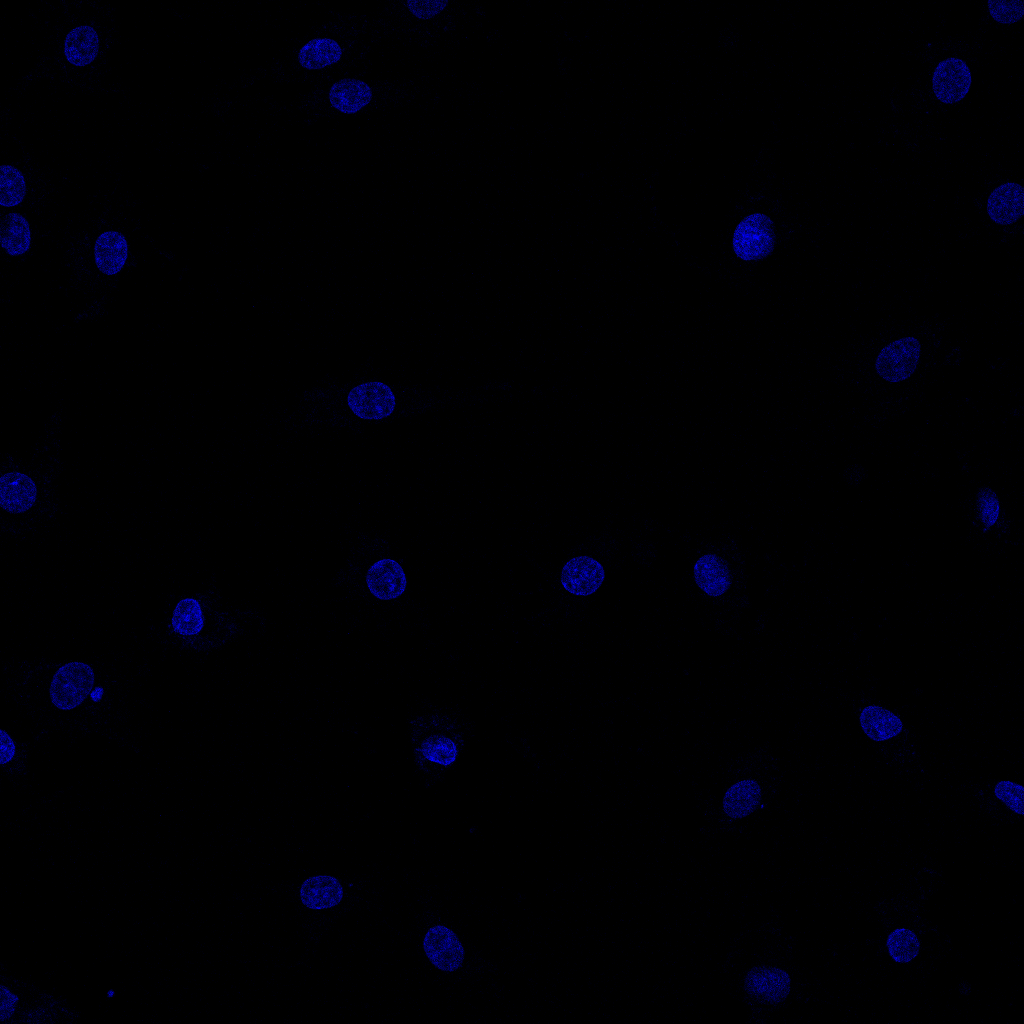

Supplement: Supplementary file 1 [file DataSheet3.ZIP › original files for Fig.3/Fig.3A 12kPa (+)PD DAPI.tif]

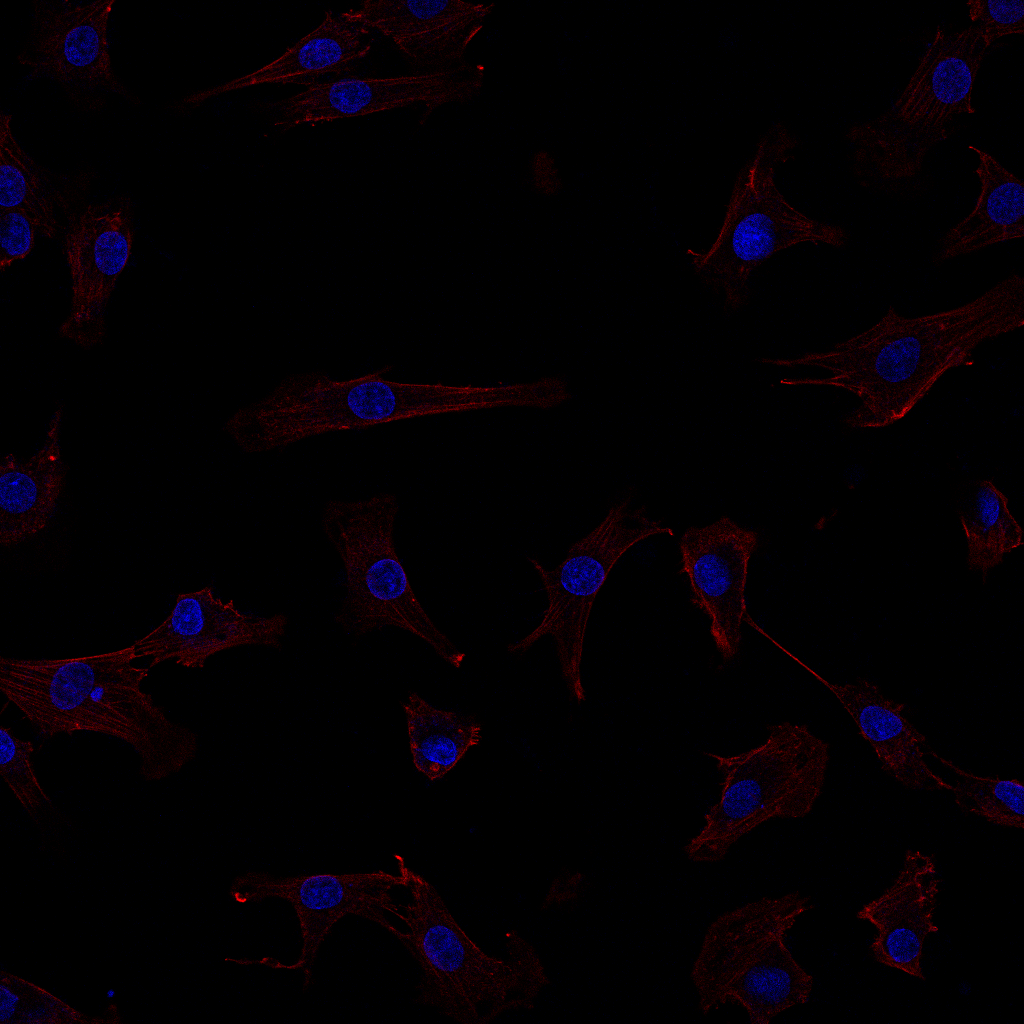

Supplement: Supplementary file 1 [file DataSheet3.ZIP › original files for Fig.3/Fig.3A 12kPa (+)PD Merged.tif]

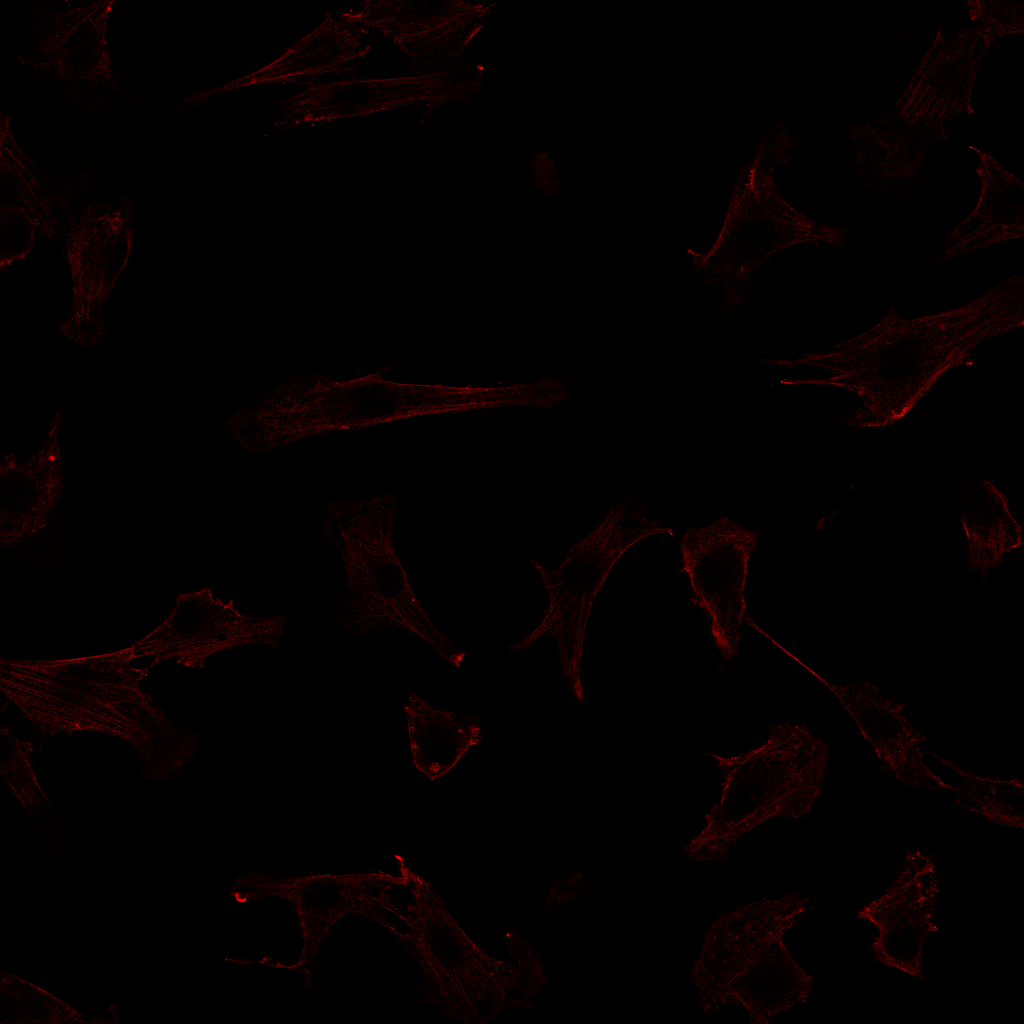

Supplement: Supplementary file 1 [file DataSheet3.ZIP › original files for Fig.3/Fig.3A 12kPa (+)PD a-SMA.tif]

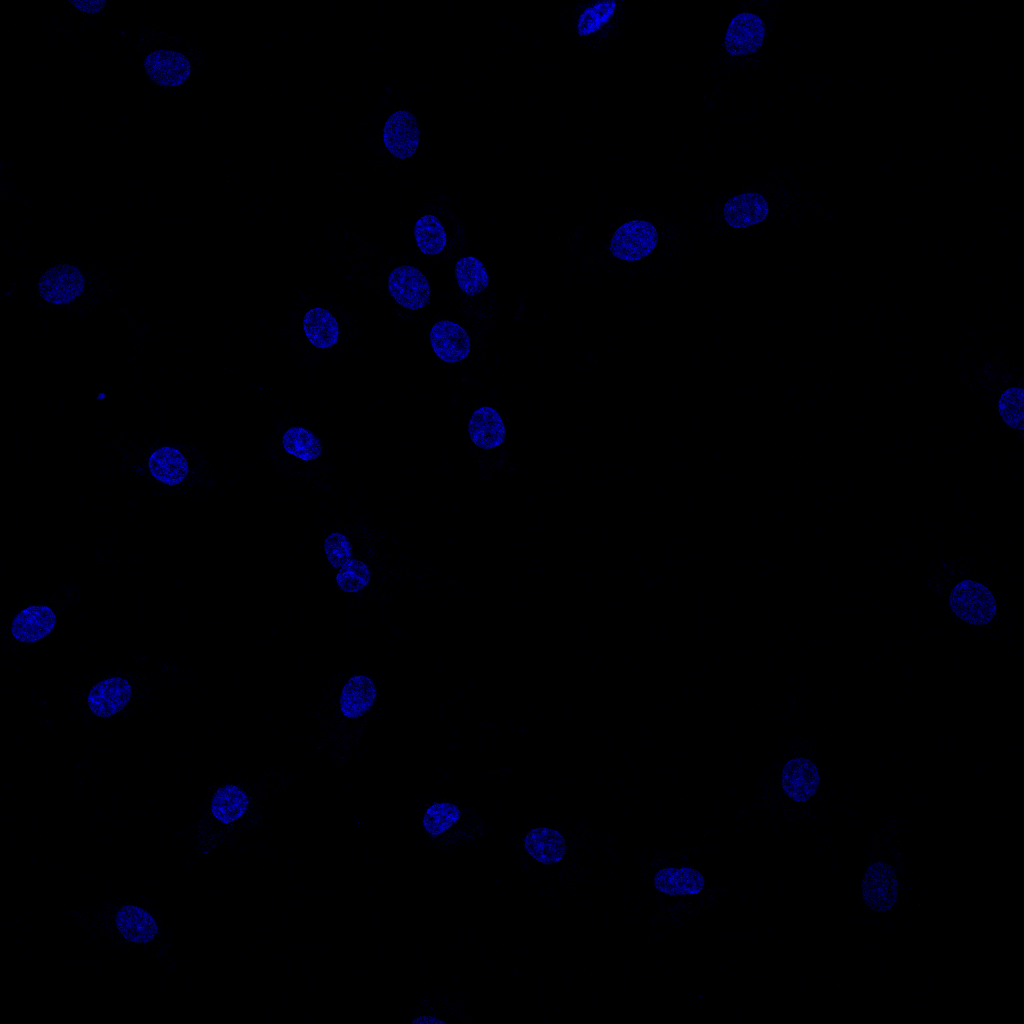

Supplement: Supplementary file 1 [file DataSheet3.ZIP › original files for Fig.3/Fig.3A 12kPa (-)PD DAPI.tif]

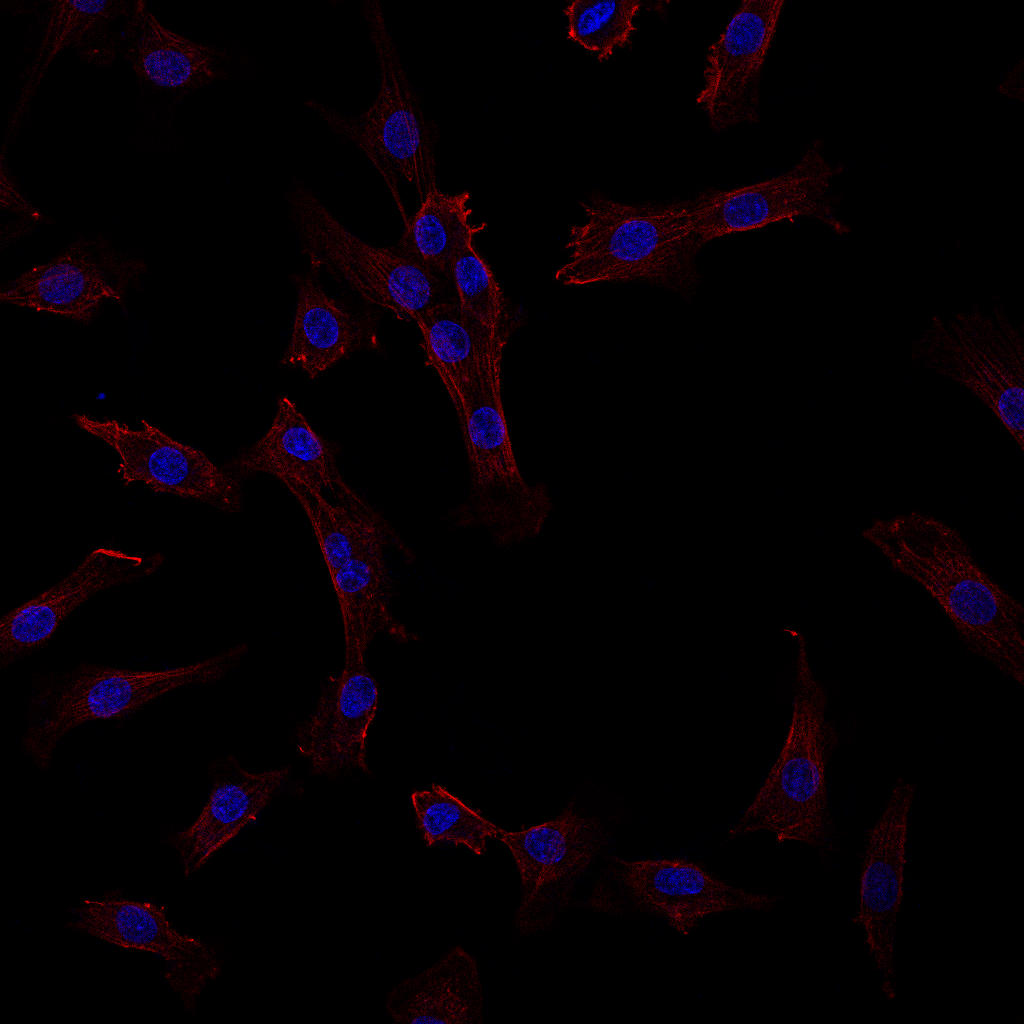

Supplement: Supplementary file 1 [file DataSheet3.ZIP › original files for Fig.3/Fig.3A 12kPa (-)PD Merged.tif]

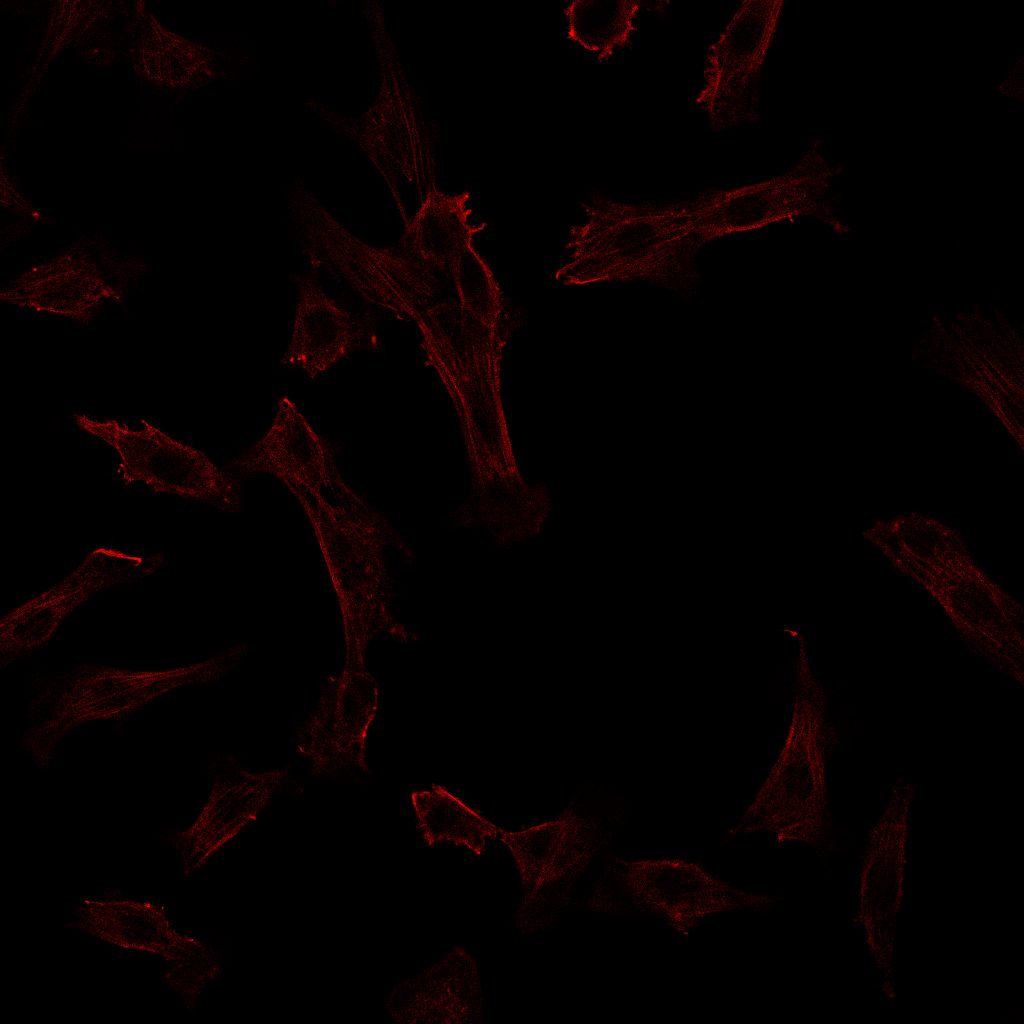

Supplement: Supplementary file 1 [file DataSheet3.ZIP › original files for Fig.3/Fig.3A 12kPa (-)PD a-SMA.tif]

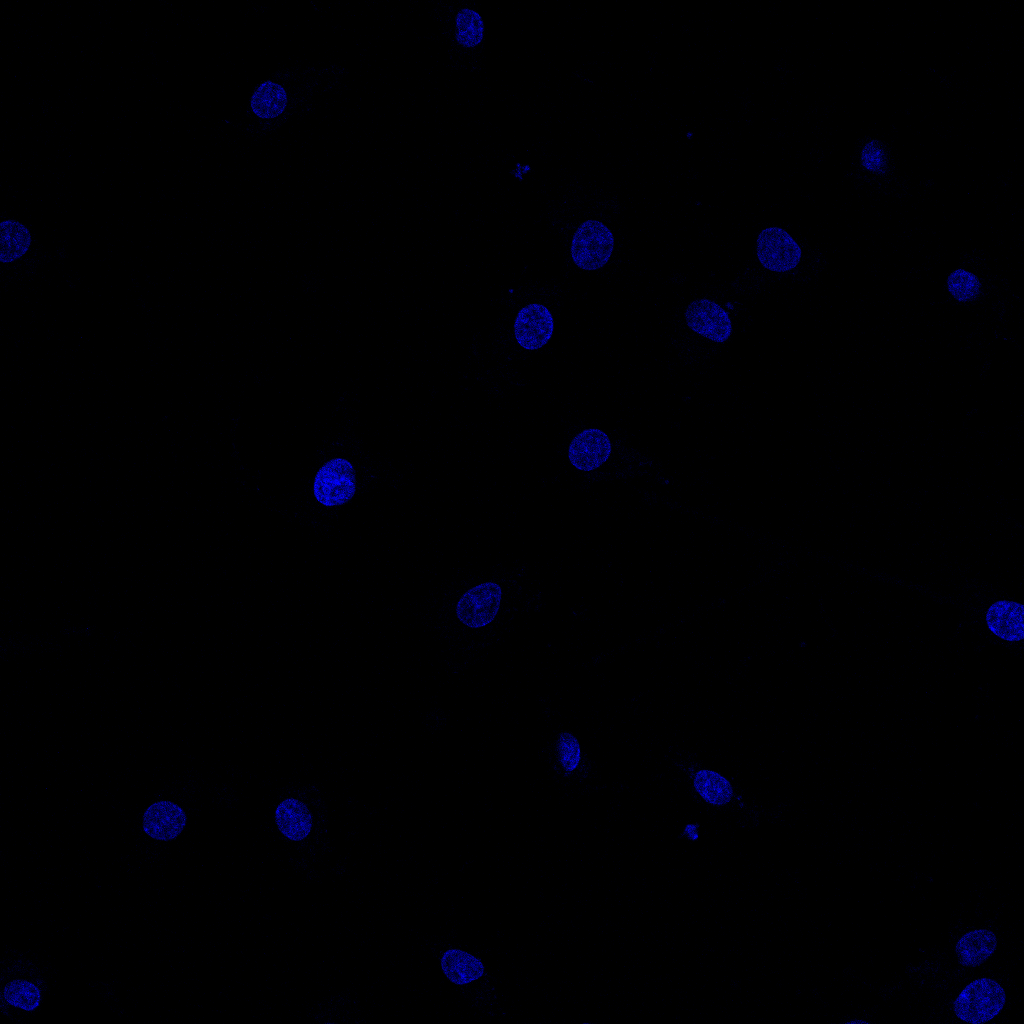

Supplement: Supplementary file 1 [file DataSheet3.ZIP › original files for Fig.3/Fig.3A 30kPa (+)PD DAPI.tif]

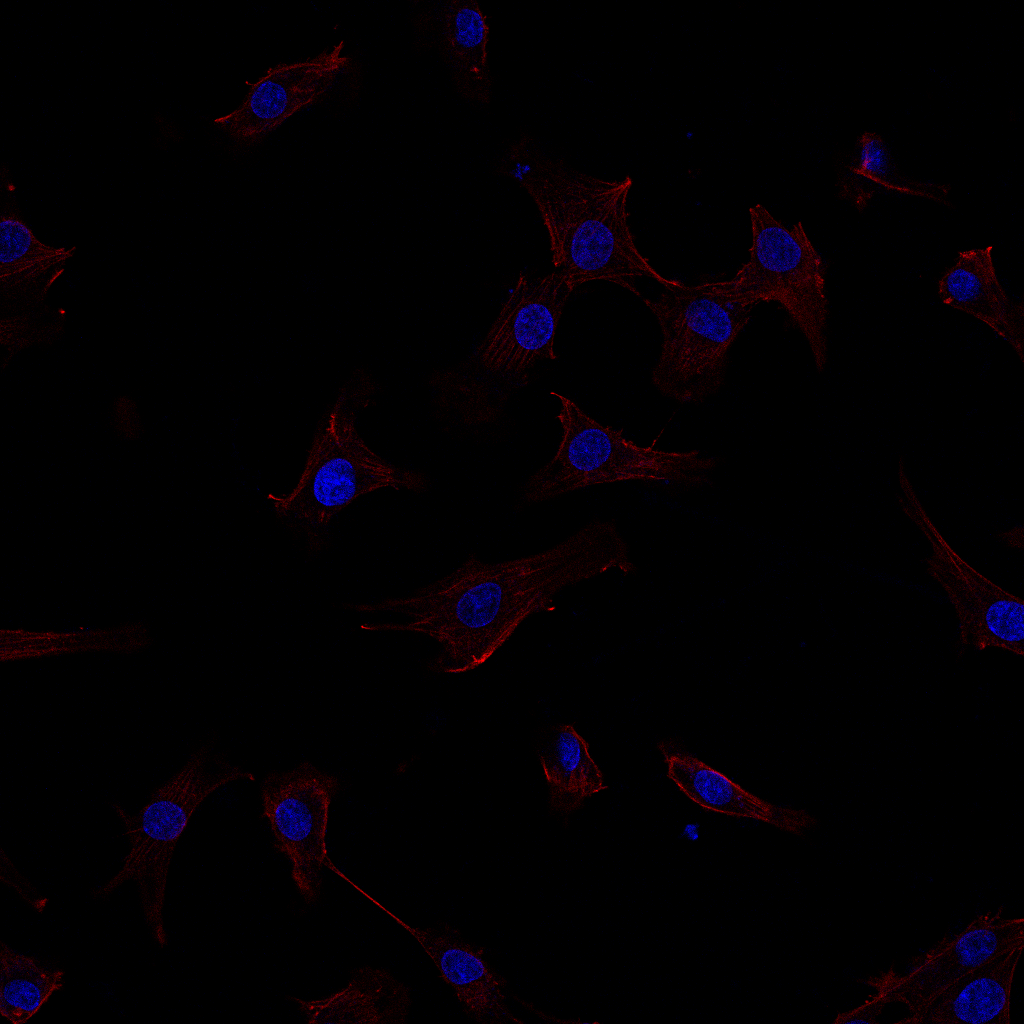

Supplement: Supplementary file 1 [file DataSheet3.ZIP › original files for Fig.3/Fig.3A 30kPa (+)PD Merged.tif]

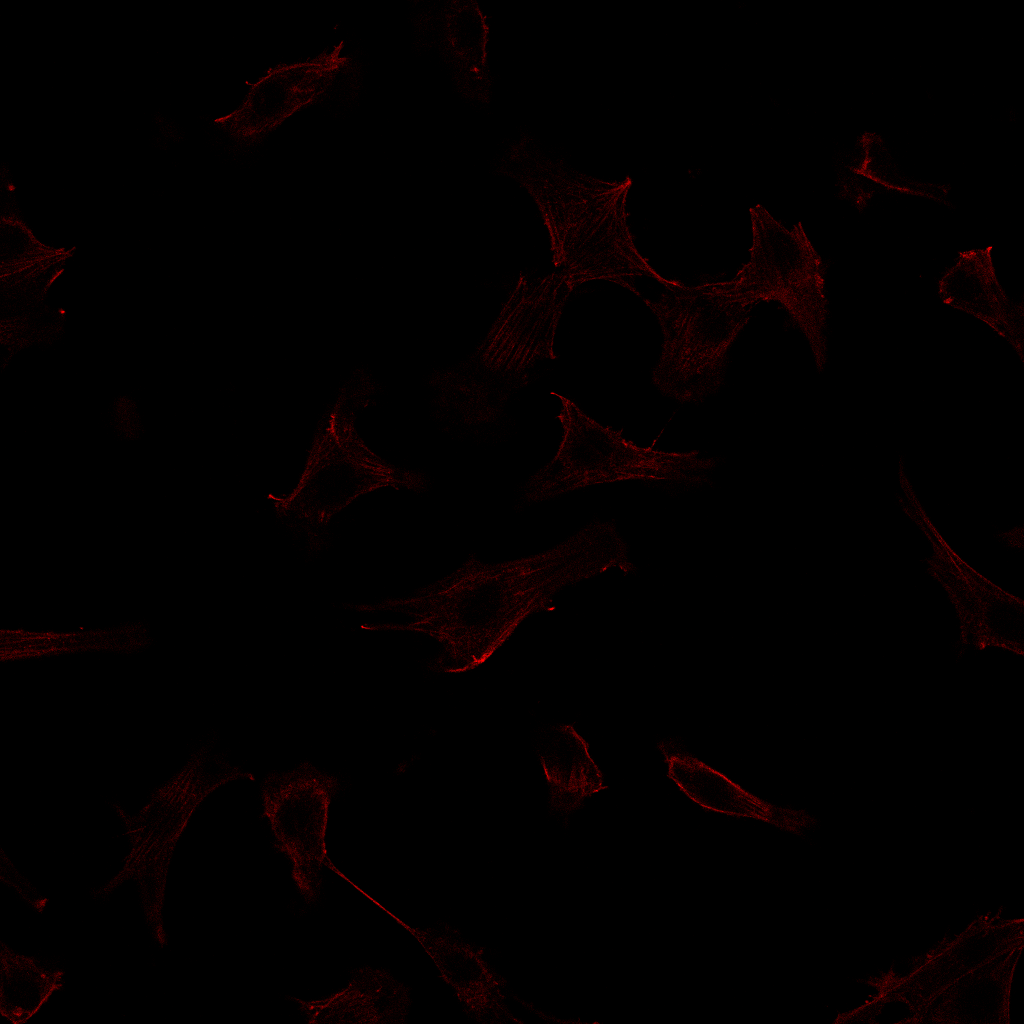

Supplement: Supplementary file 1 [file DataSheet3.ZIP › original files for Fig.3/Fig.3A 30kPa (+)PD a-SMA.tif]

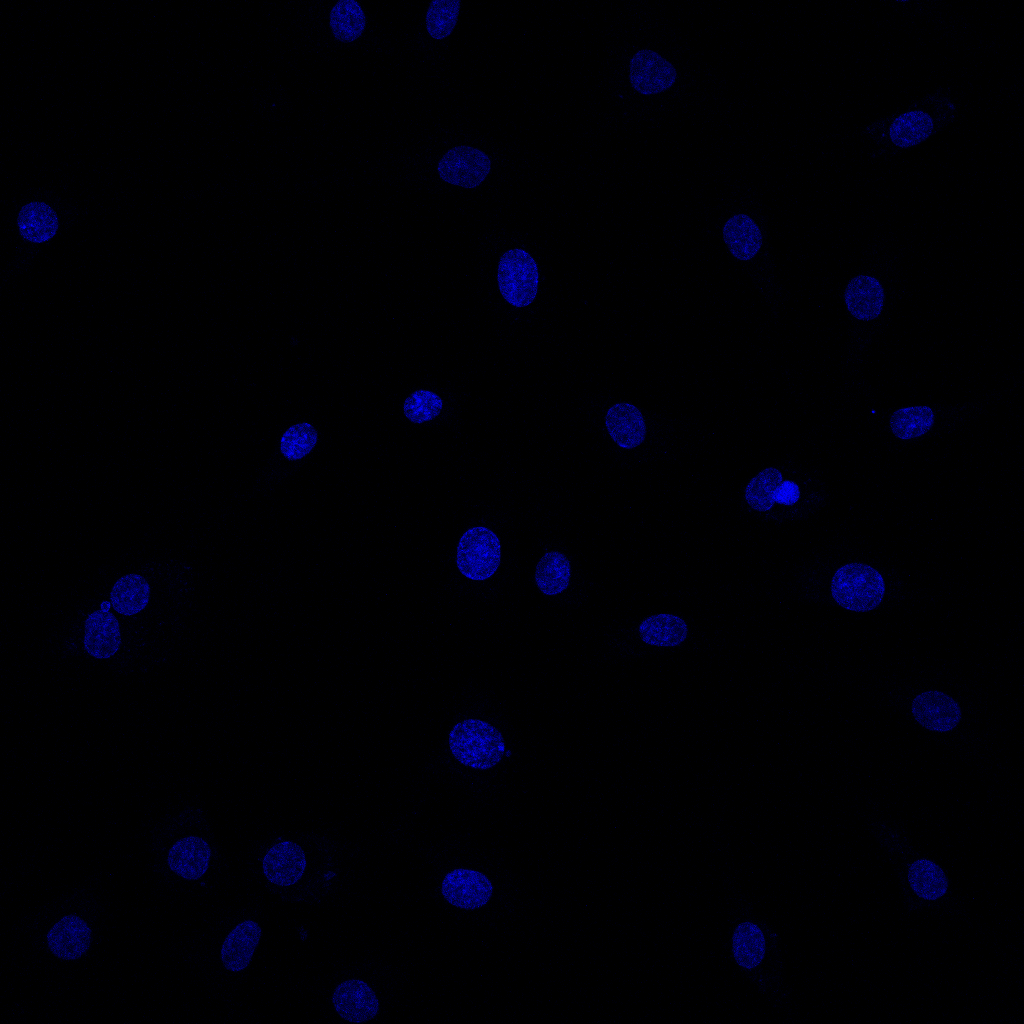

Supplement: Supplementary file 1 [file DataSheet3.ZIP › original files for Fig.3/Fig.3A 30kPa (-)PD DAPI.tif]

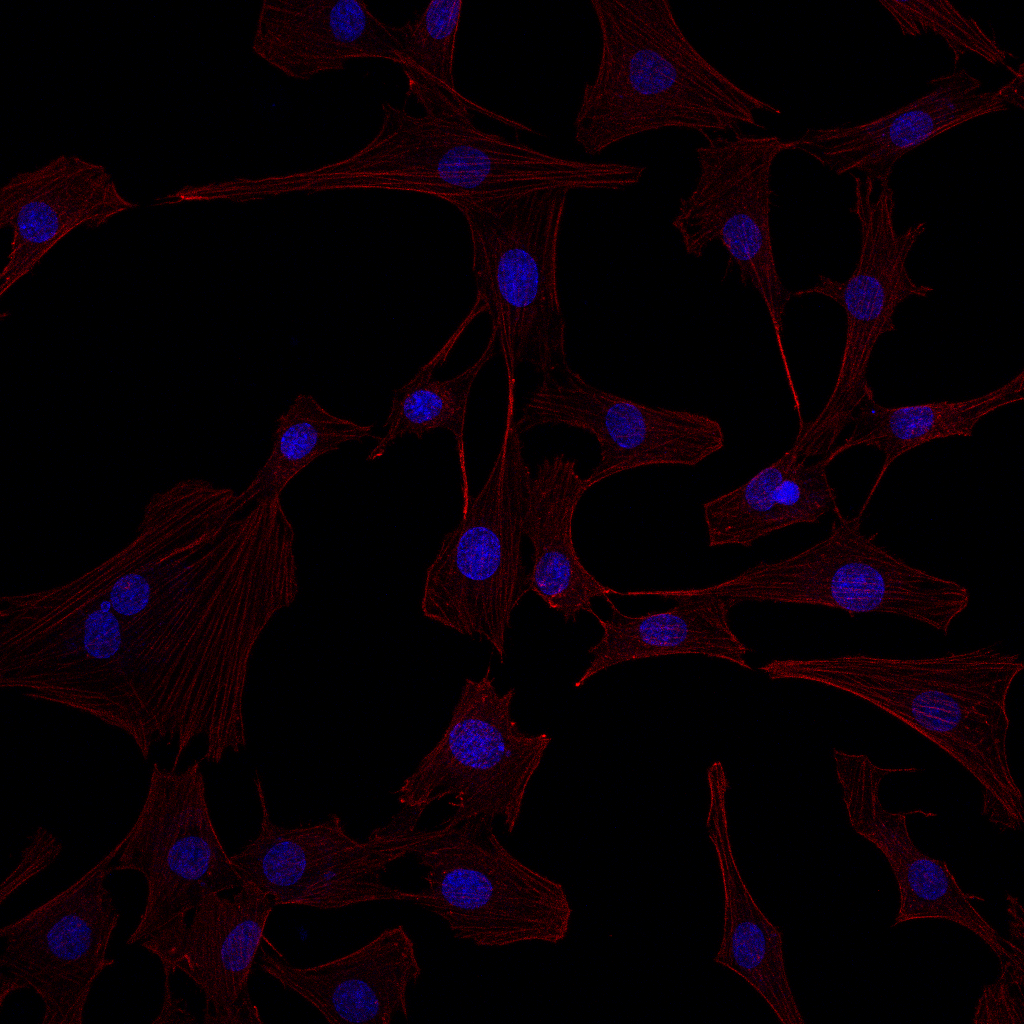

Supplement: Supplementary file 1 [file DataSheet3.ZIP › original files for Fig.3/Fig.3A 30kPa (-)PD Merged.tif]

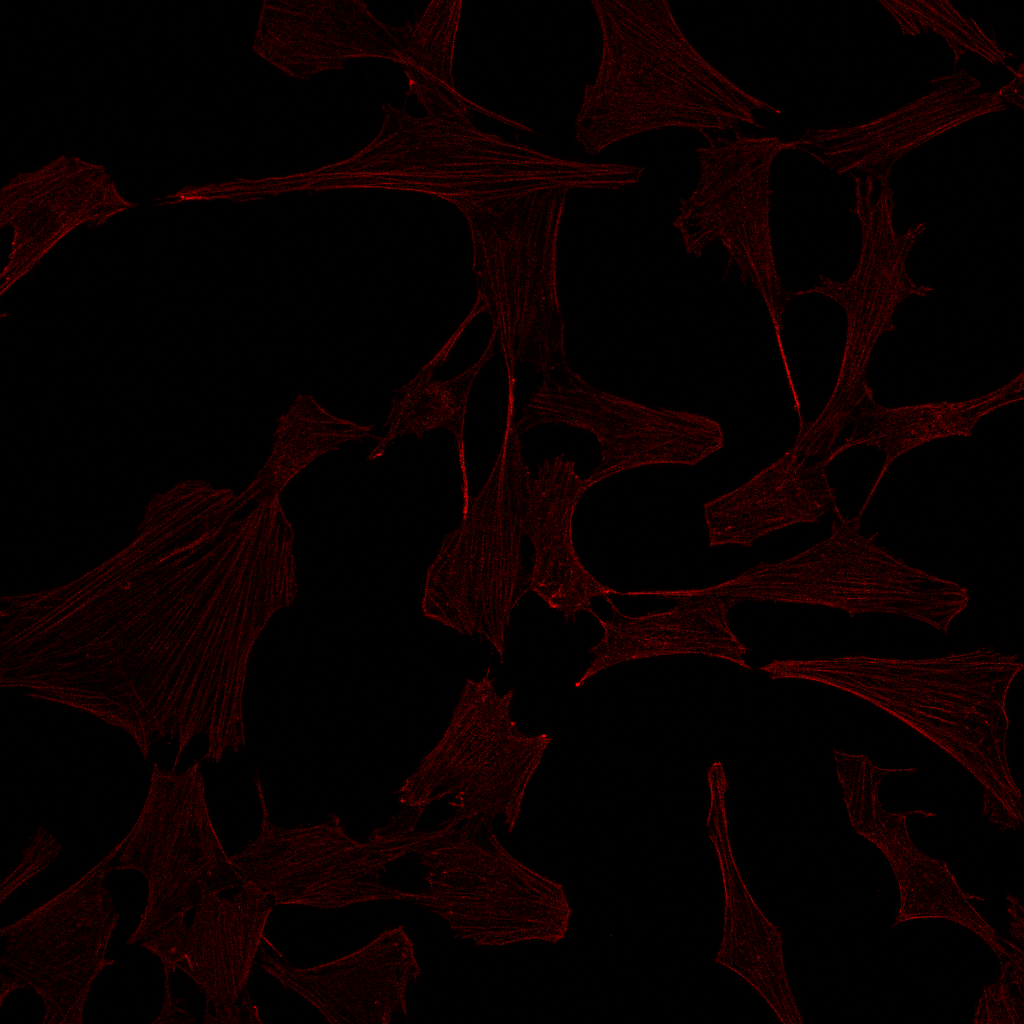

Supplement: Supplementary file 1 [file DataSheet3.ZIP › original files for Fig.3/Fig.3A 30kPa (-)PD a-SMA.tif]

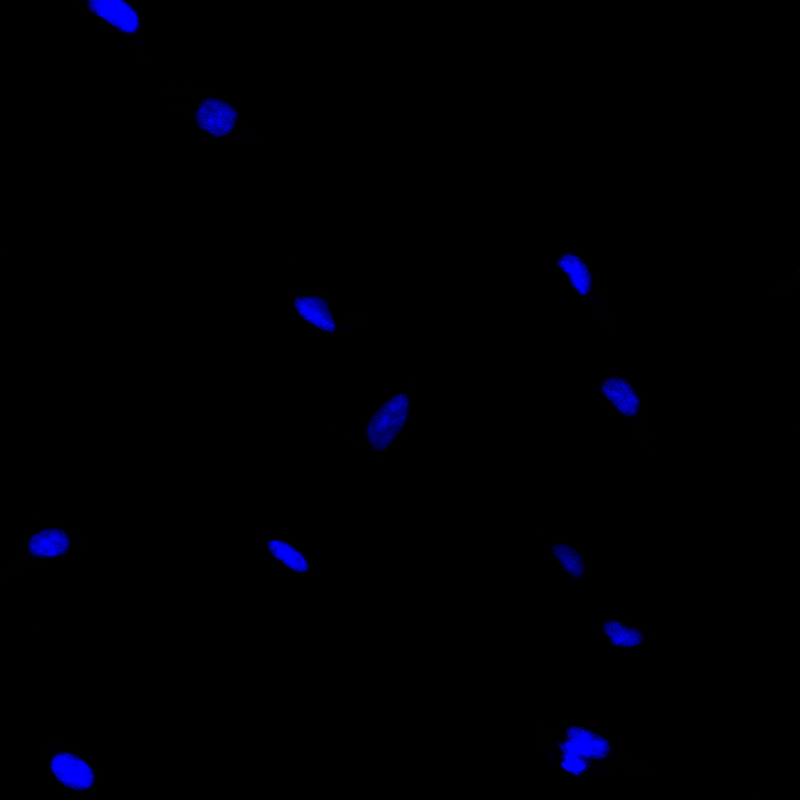

Supplement: Supplementary file 1 [file DataSheet3.ZIP › original files for Fig.3/Fig.3A 3kPa (+)PD DAPI.tif]

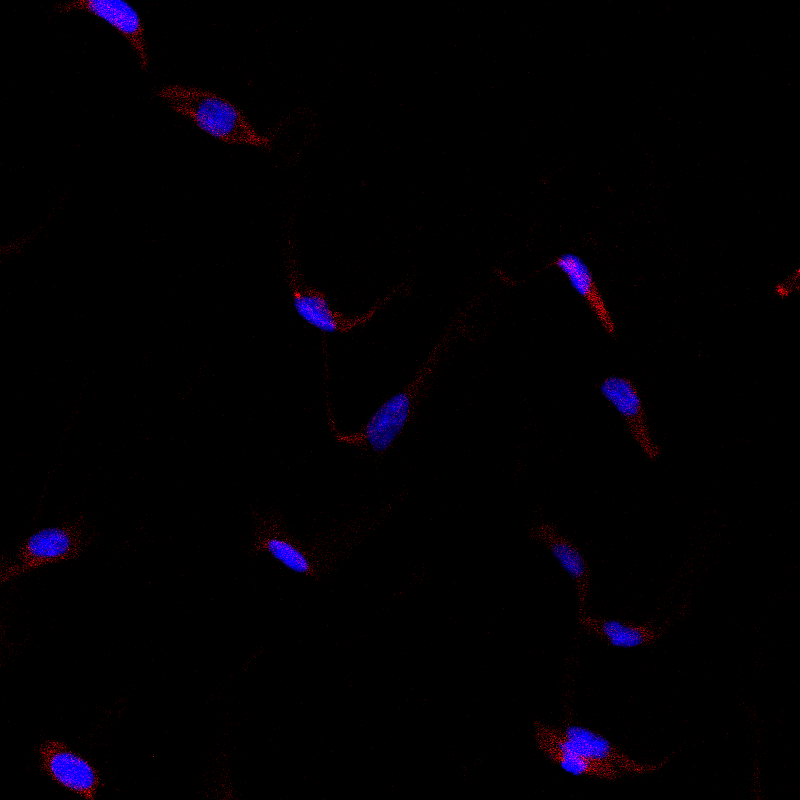

Supplement: Supplementary file 1 [file DataSheet3.ZIP › original files for Fig.3/Fig.3A 3kPa (+)PD Merged.tif]

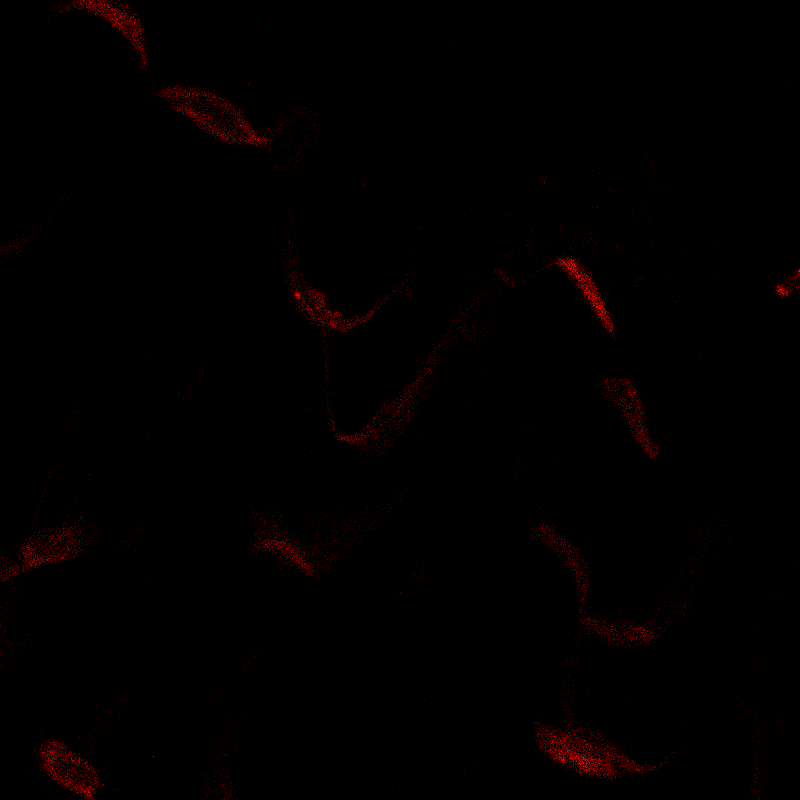

Supplement: Supplementary file 1 [file DataSheet3.ZIP › original files for Fig.3/Fig.3A 3kPa (+)PD a-SMA.tif]

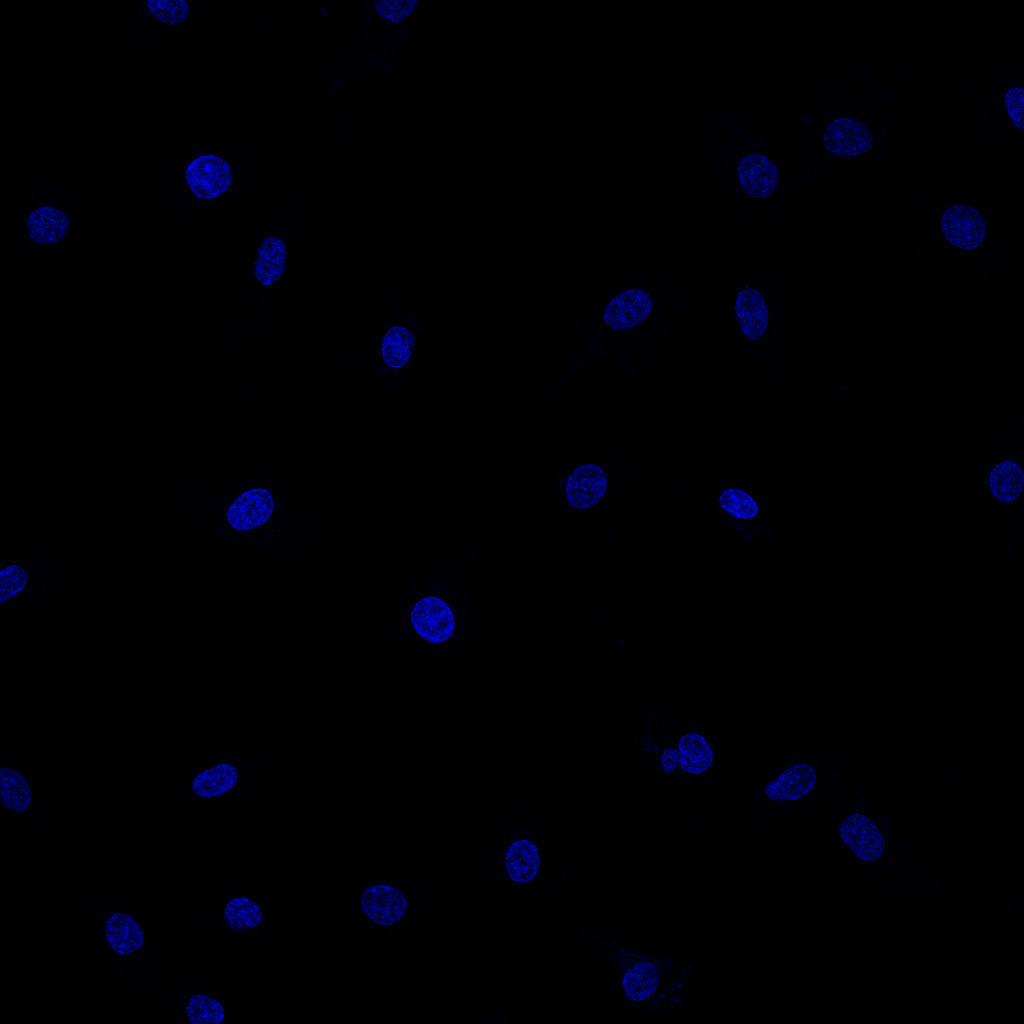

Supplement: Supplementary file 1 [file DataSheet3.ZIP › original files for Fig.3/Fig.3A 3kPa (-)PD DAPI.tif]

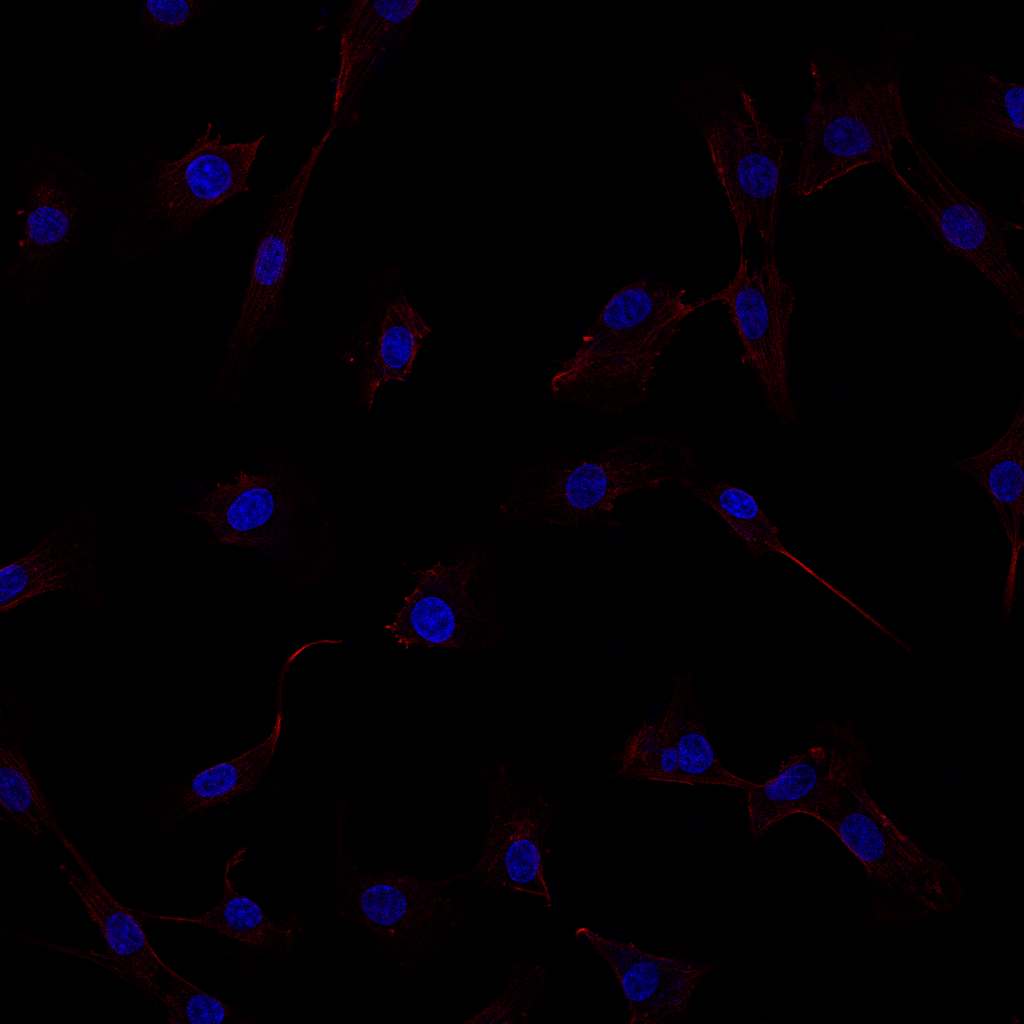

Supplement: Supplementary file 1 [file DataSheet3.ZIP › original files for Fig.3/Fig.3A 3kPa (-)PD Merged.tif]

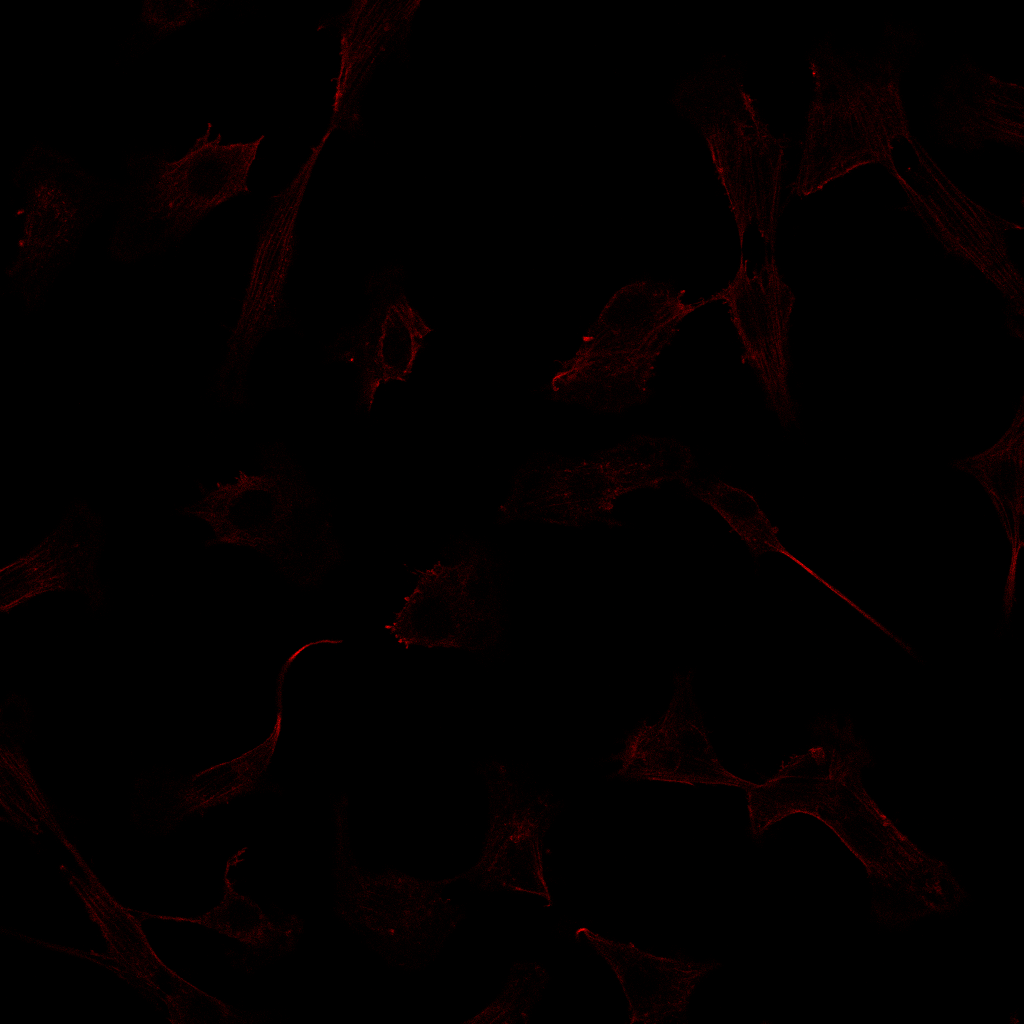

Supplement: Supplementary file 1 [file DataSheet3.ZIP › original files for Fig.3/Fig.3A 3kPa (-)PD a-SMA.tif]

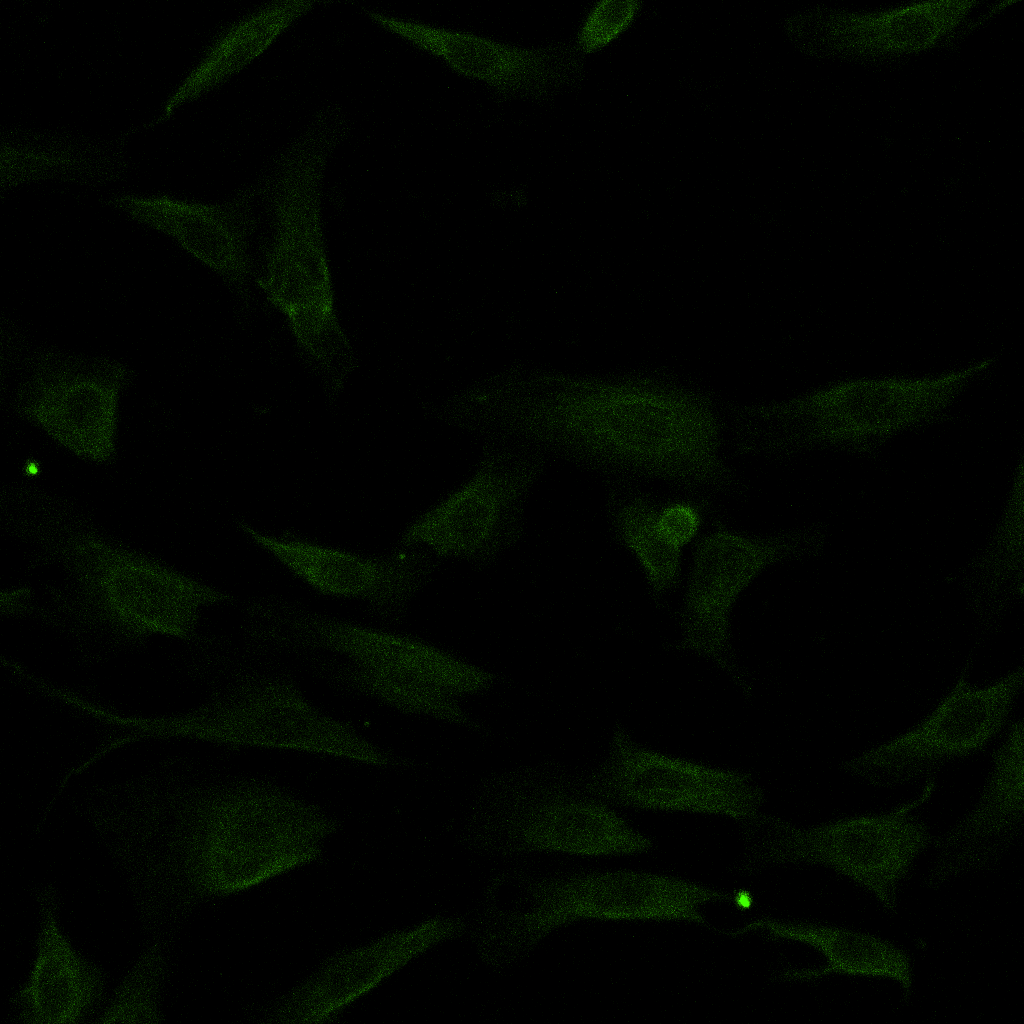

Supplement: Supplementary file 1 [file DataSheet3.ZIP › original files for Fig.3/Fig.3C 12kPa (+)PD Col I.tif]

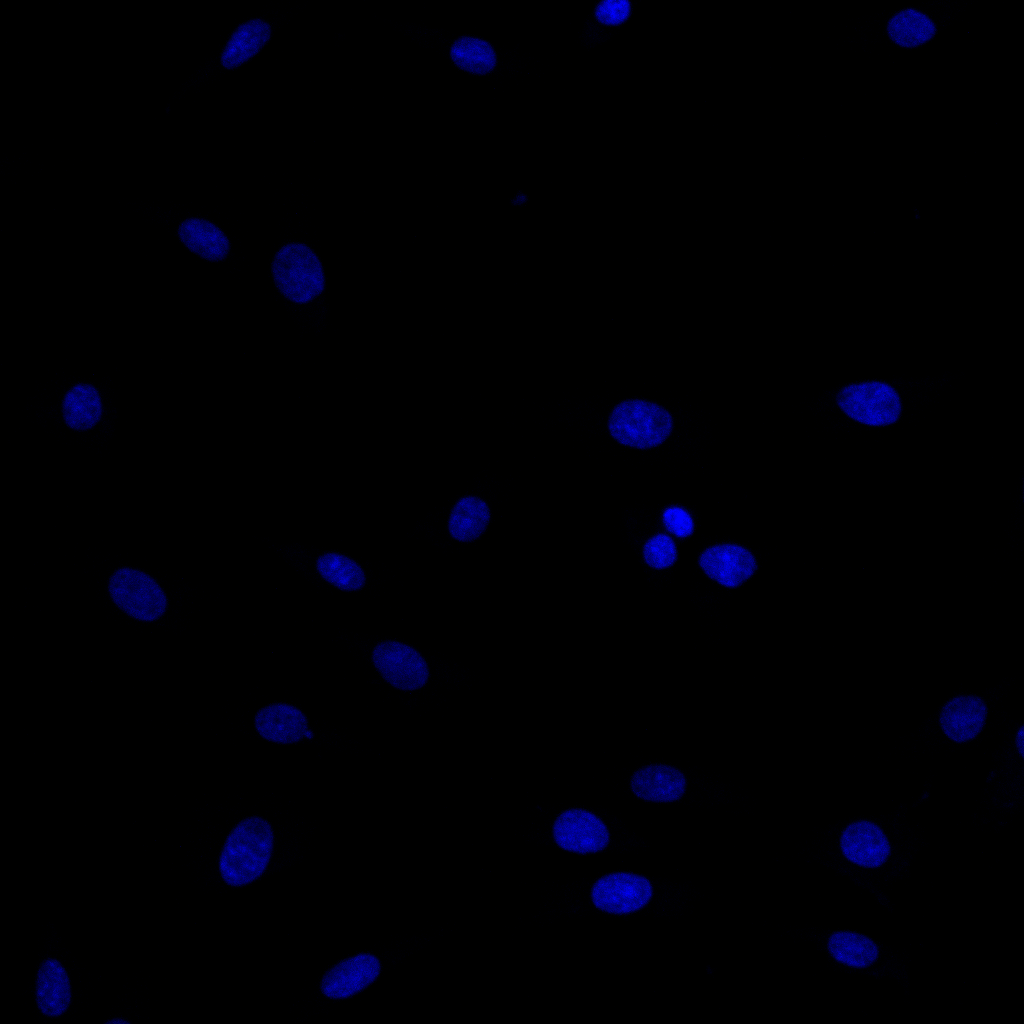

Supplement: Supplementary file 1 [file DataSheet3.ZIP › original files for Fig.3/Fig.3C 12kPa (+)PD DAPI.tif]

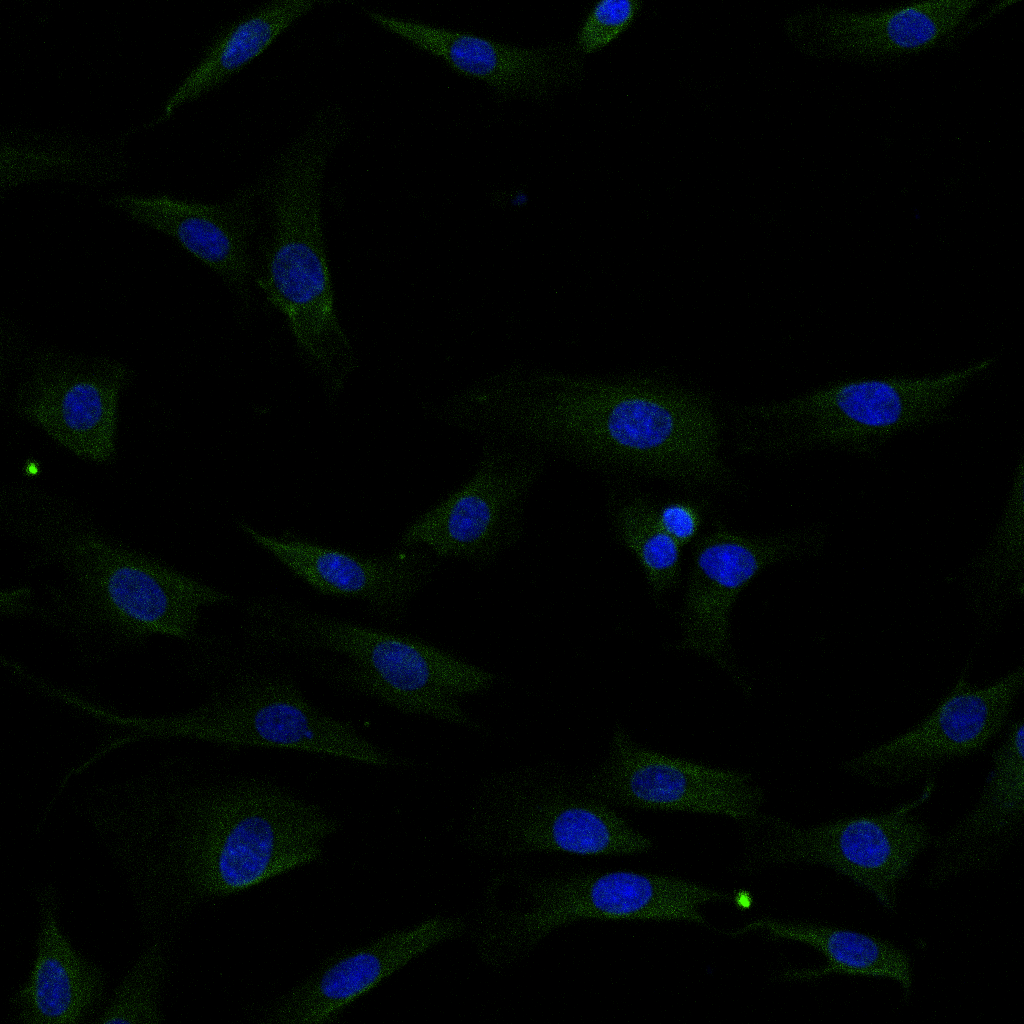

Supplement: Supplementary file 1 [file DataSheet3.ZIP › original files for Fig.3/Fig.3C 12kPa (+)PD Merged.tif]

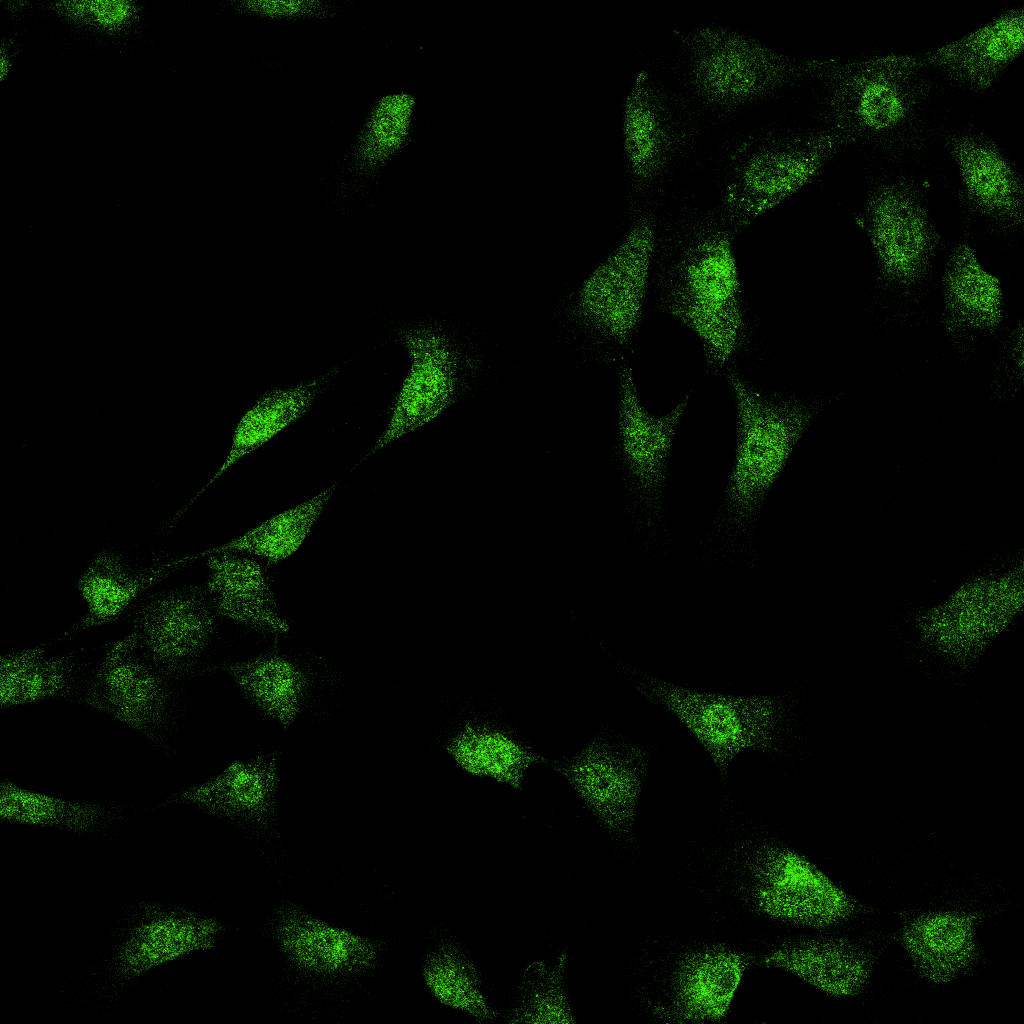

Supplement: Supplementary file 1 [file DataSheet3.ZIP › original files for Fig.3/Fig.3C 12kPa (-)PD Col I.tif]

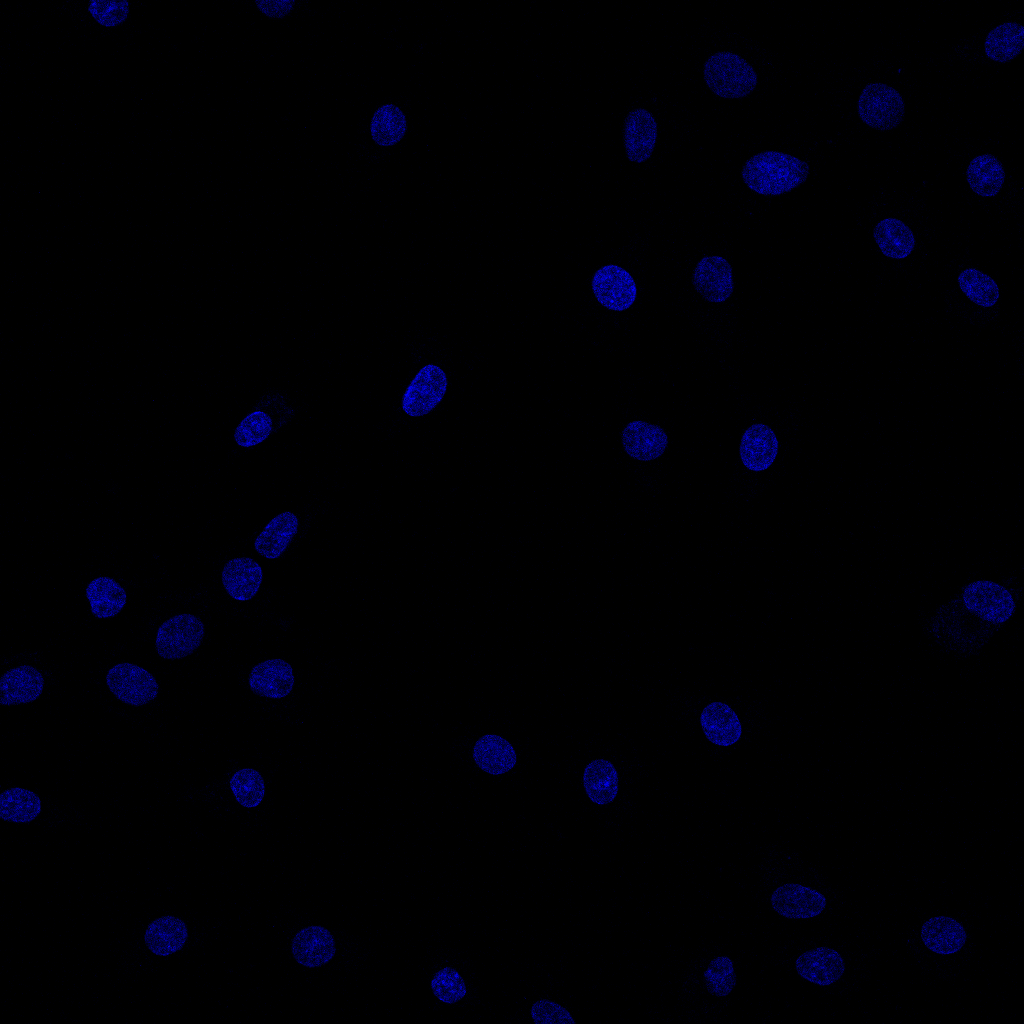

Supplement: Supplementary file 1 [file DataSheet3.ZIP › original files for Fig.3/Fig.3C 12kPa (-)PD DAPI .tif]

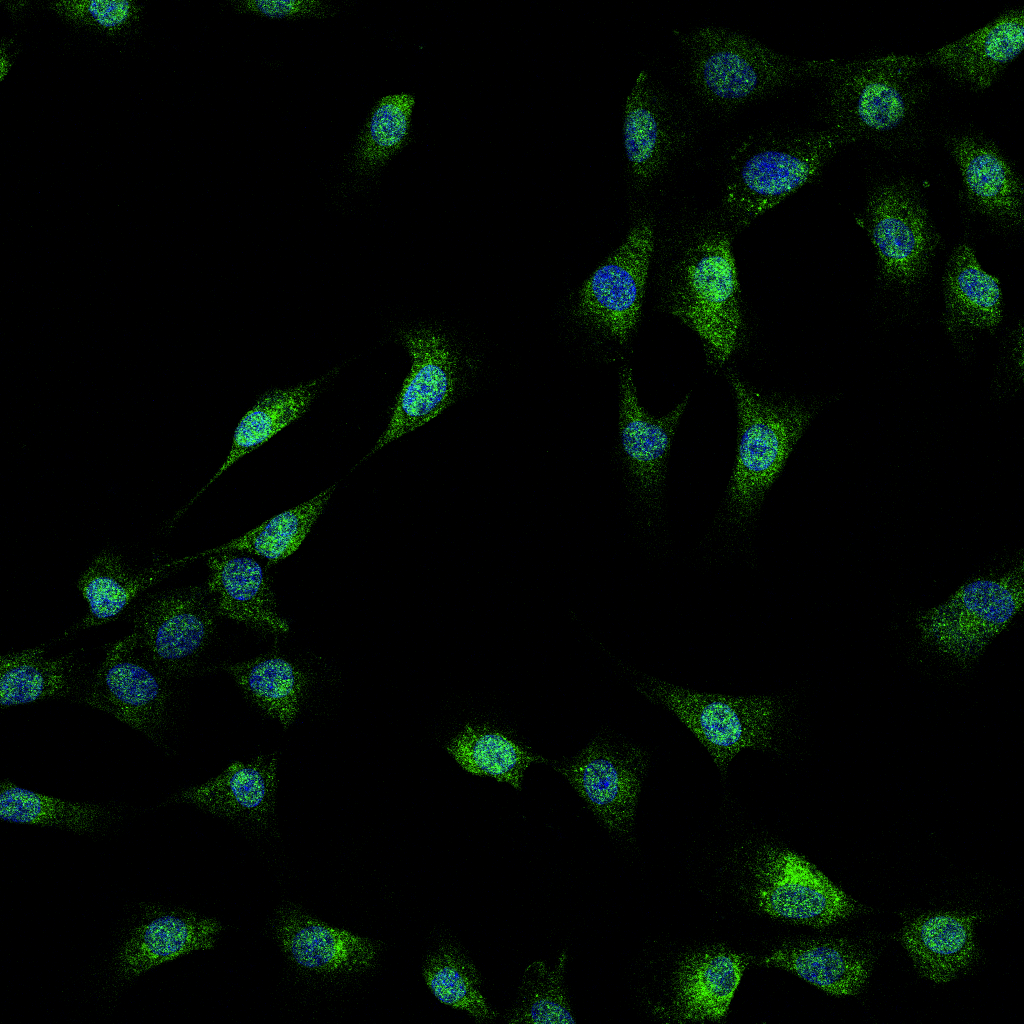

Supplement: Supplementary file 1 [file DataSheet3.ZIP › original files for Fig.3/Fig.3C 12kPa (-)PD Merged.tif]

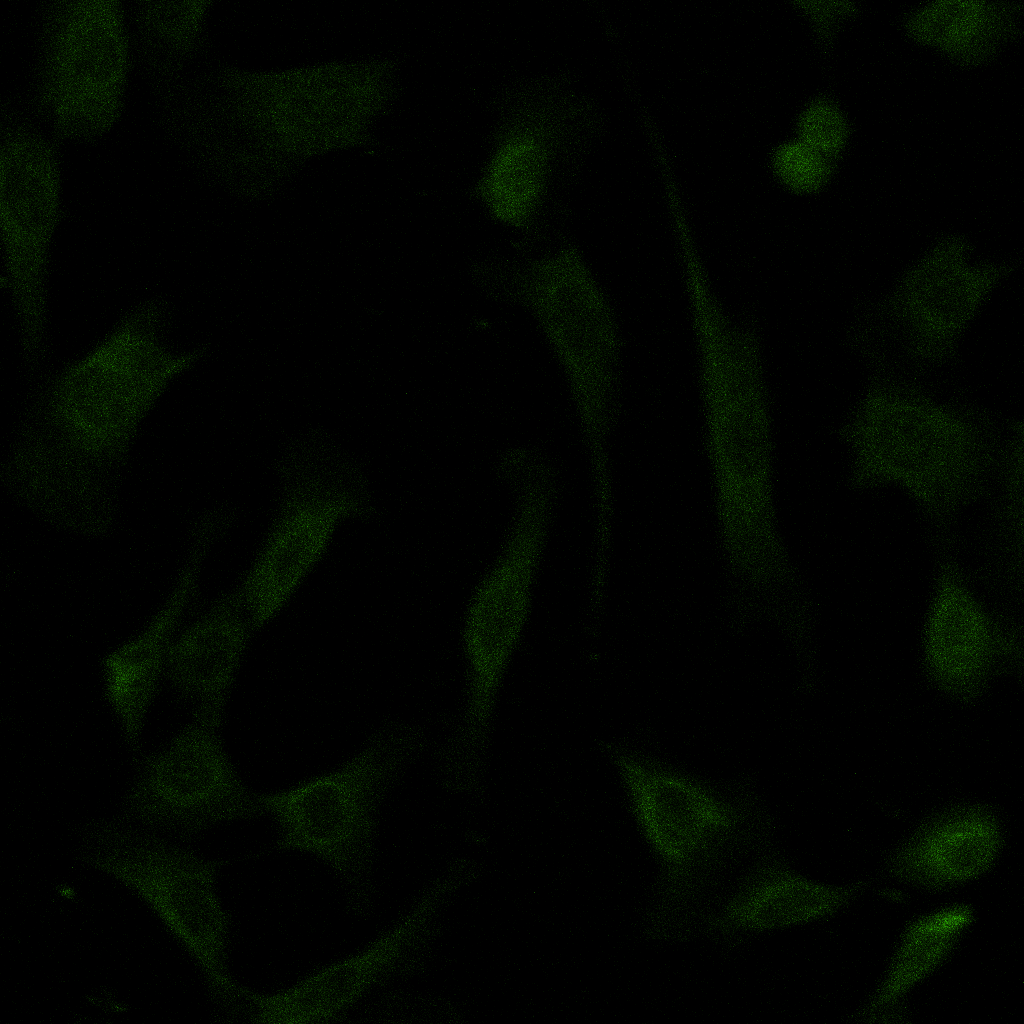

Supplement: Supplementary file 1 [file DataSheet3.ZIP › original files for Fig.3/Fig.3C 30kPa (+)PD Col I.tif]

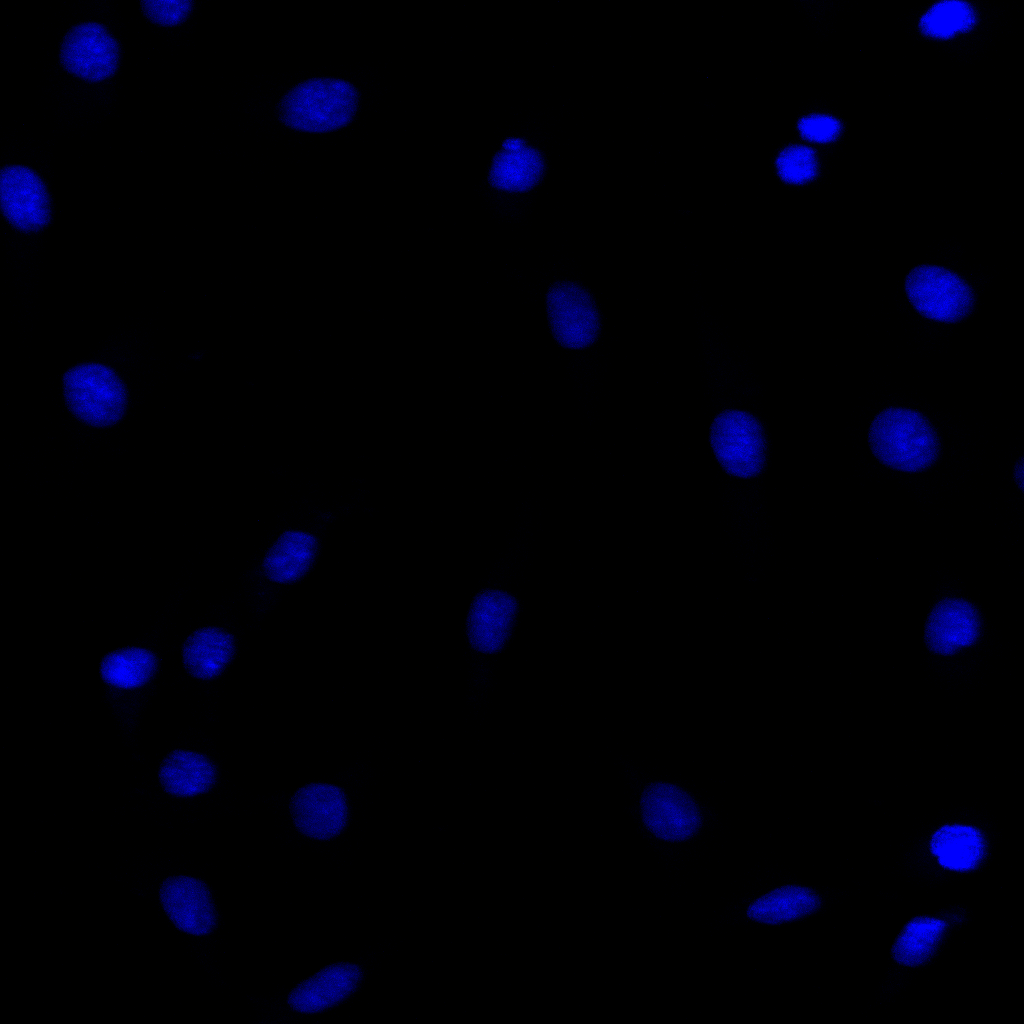

Supplement: Supplementary file 1 [file DataSheet3.ZIP › original files for Fig.3/Fig.3C 30kPa (+)PD DAPI.tif]

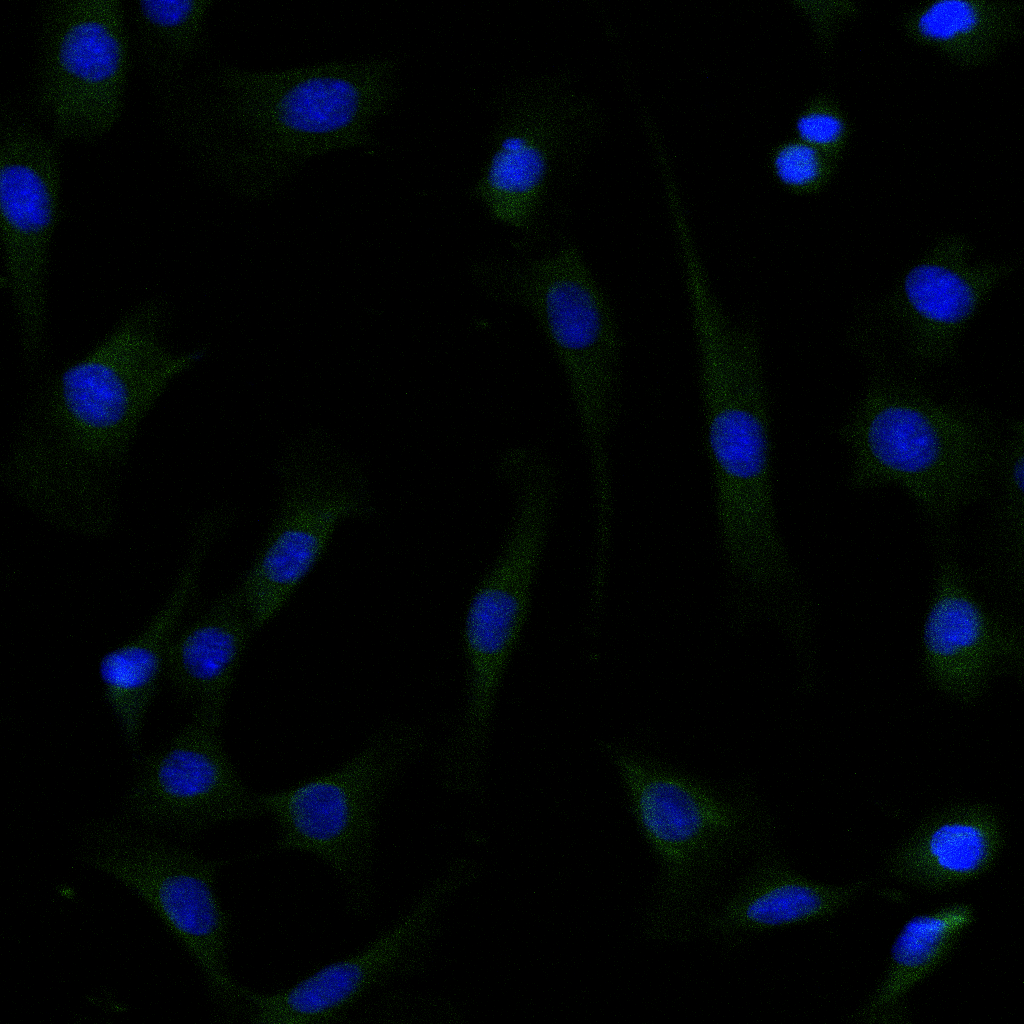

Supplement: Supplementary file 1 [file DataSheet3.ZIP › original files for Fig.3/Fig.3C 30kPa (+)PD Merged.tif]

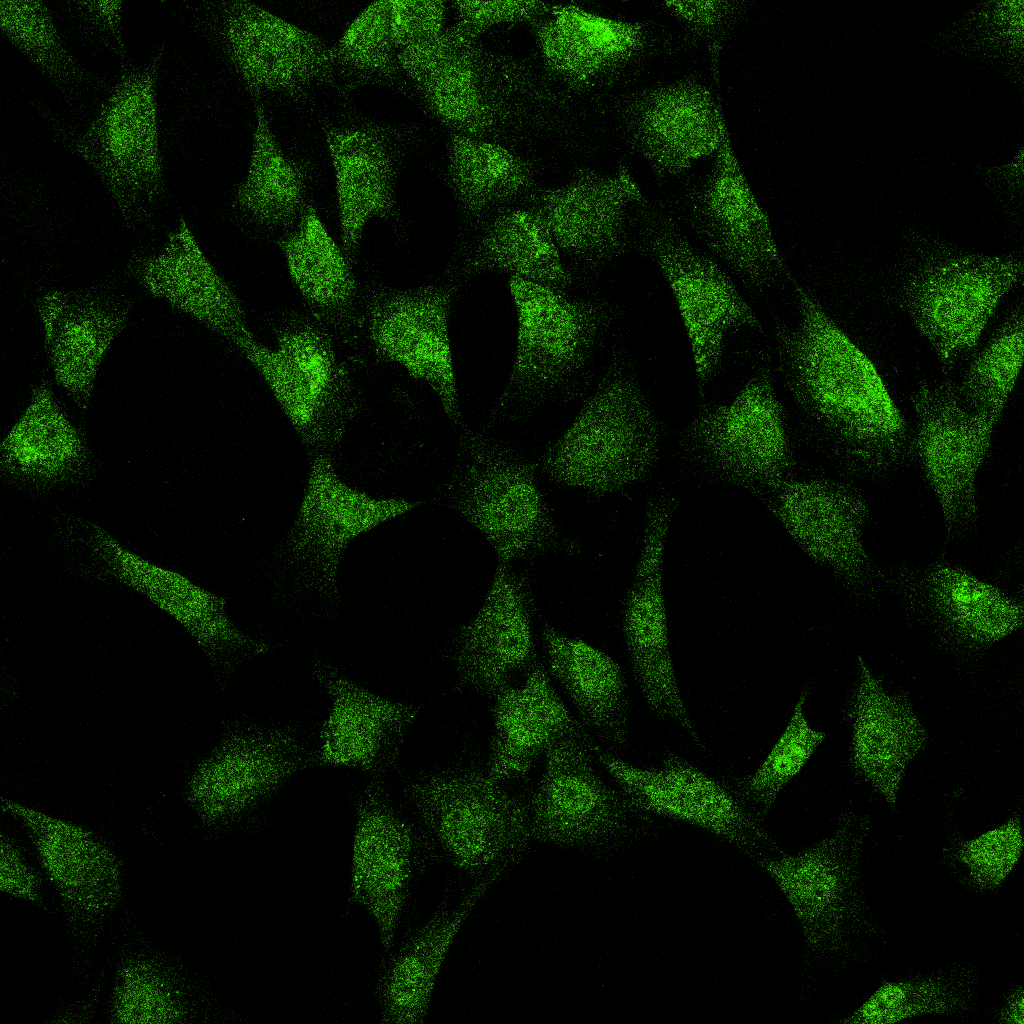

Supplement: Supplementary file 1 [file DataSheet3.ZIP › original files for Fig.3/Fig.3C 30kPa (-)PD Col I.tif]

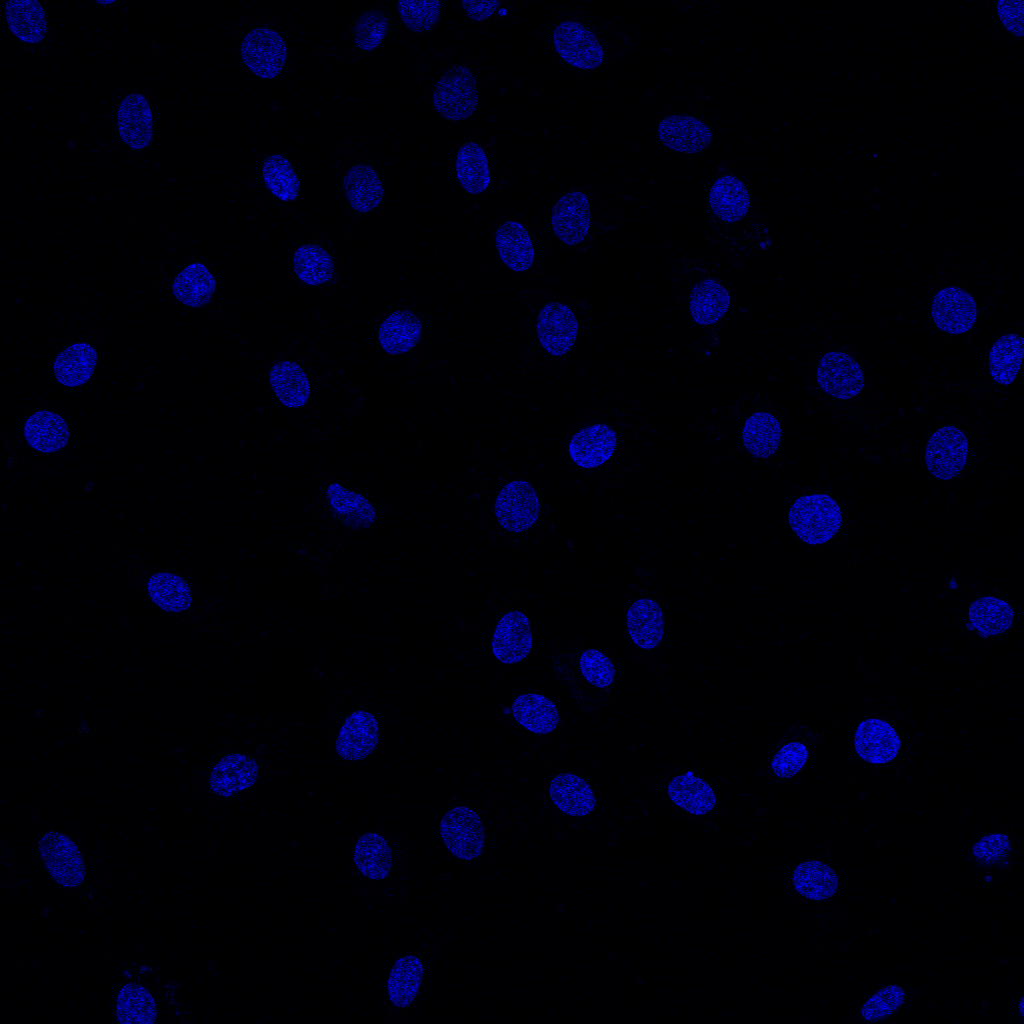

Supplement: Supplementary file 1 [file DataSheet3.ZIP › original files for Fig.3/Fig.3C 30kPa (-)PD DAPI.tif]

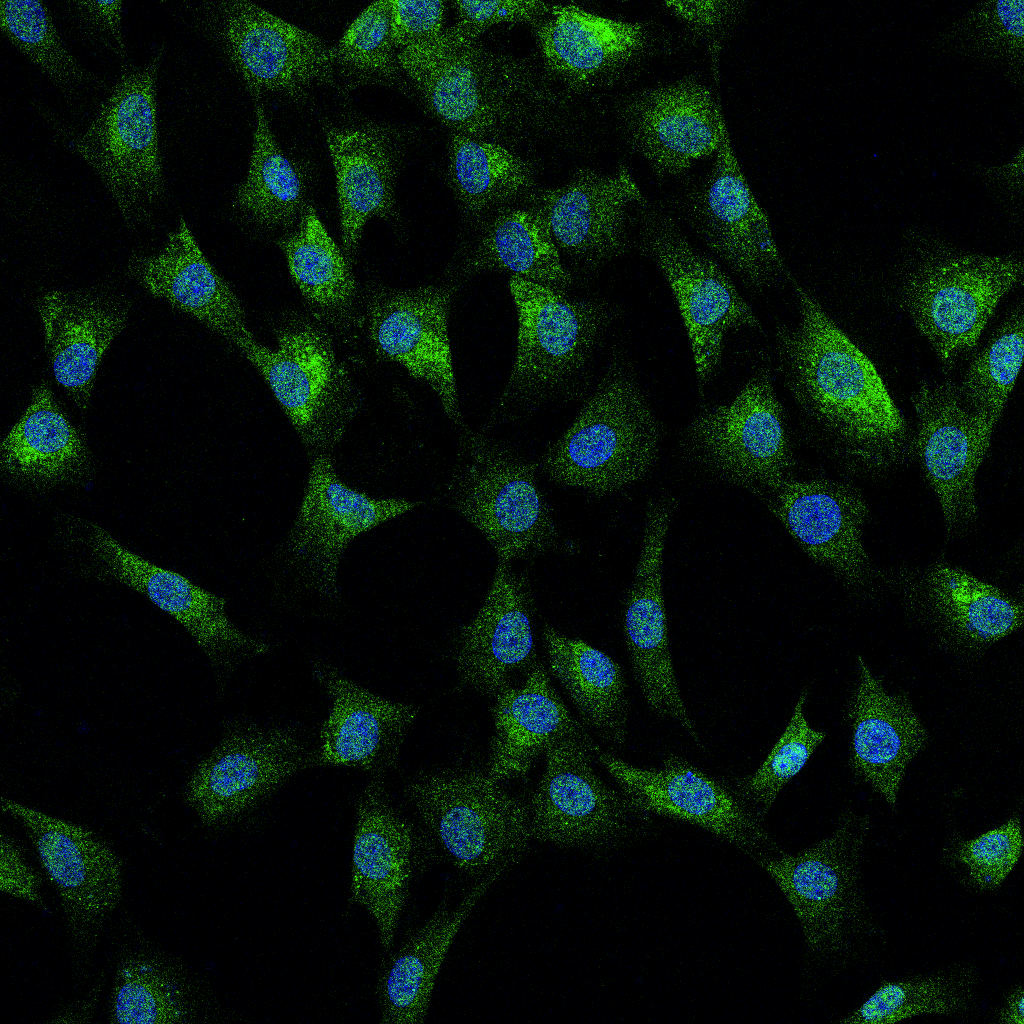

Supplement: Supplementary file 1 [file DataSheet3.ZIP › original files for Fig.3/Fig.3C 30kPa (-)PD Merged.tif]

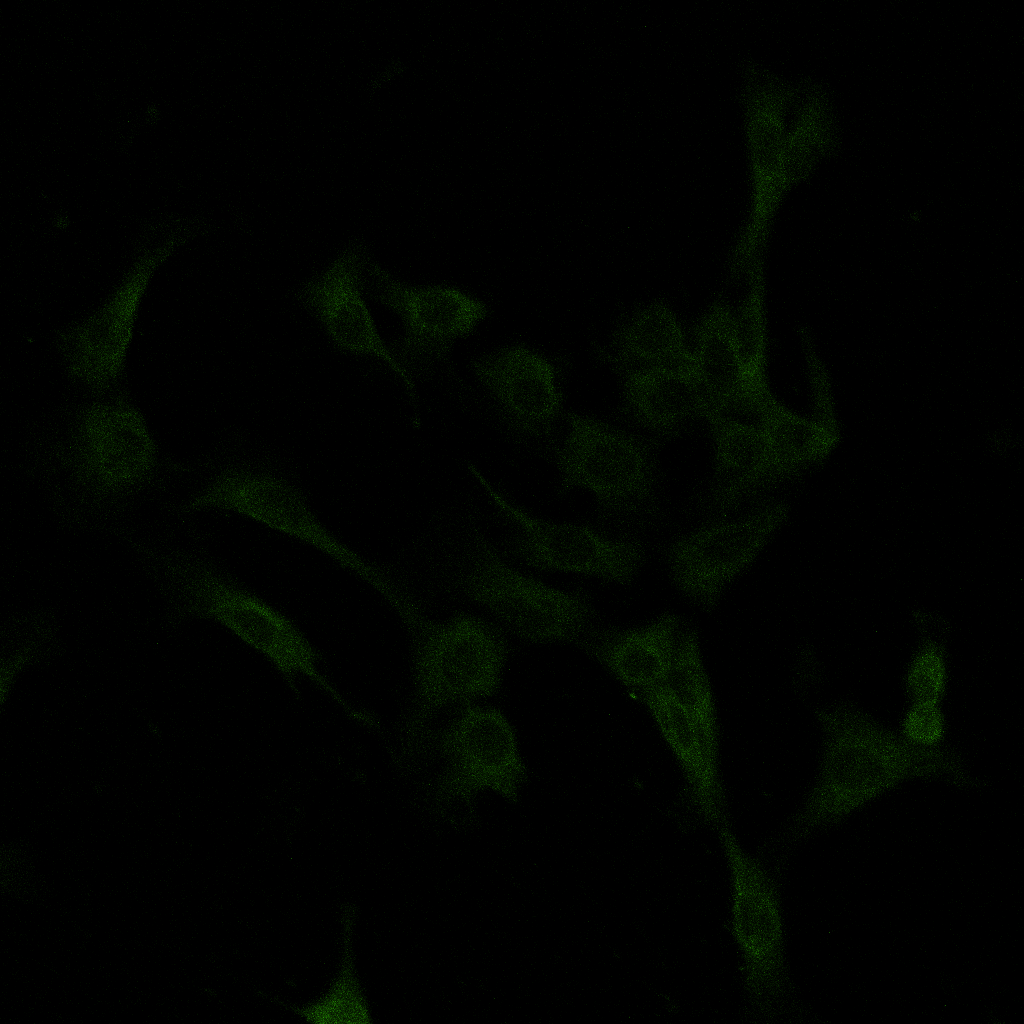

Supplement: Supplementary file 1 [file DataSheet3.ZIP › original files for Fig.3/Fig.3C 3kPa (+)PD Col I.tif]

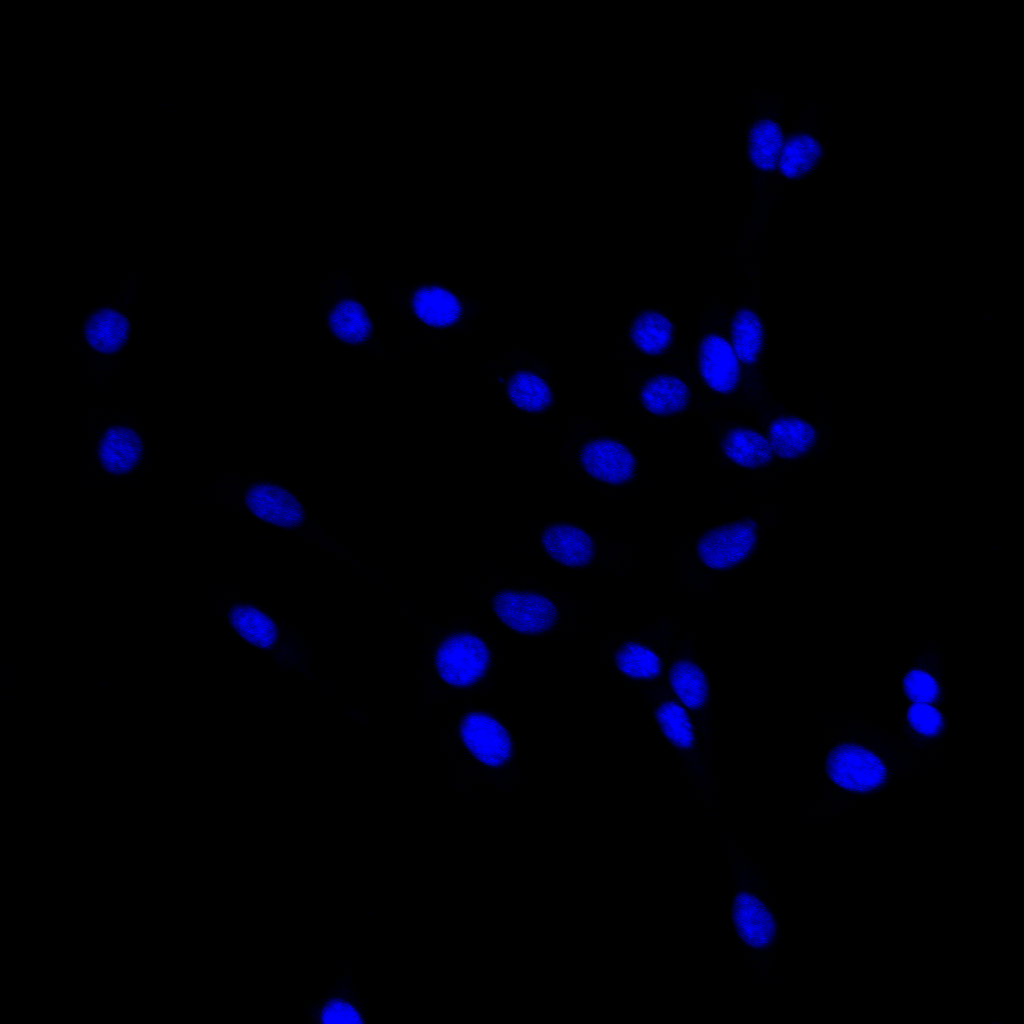

Supplement: Supplementary file 1 [file DataSheet3.ZIP › original files for Fig.3/Fig.3C 3kPa (+)PD DAPI.tif]

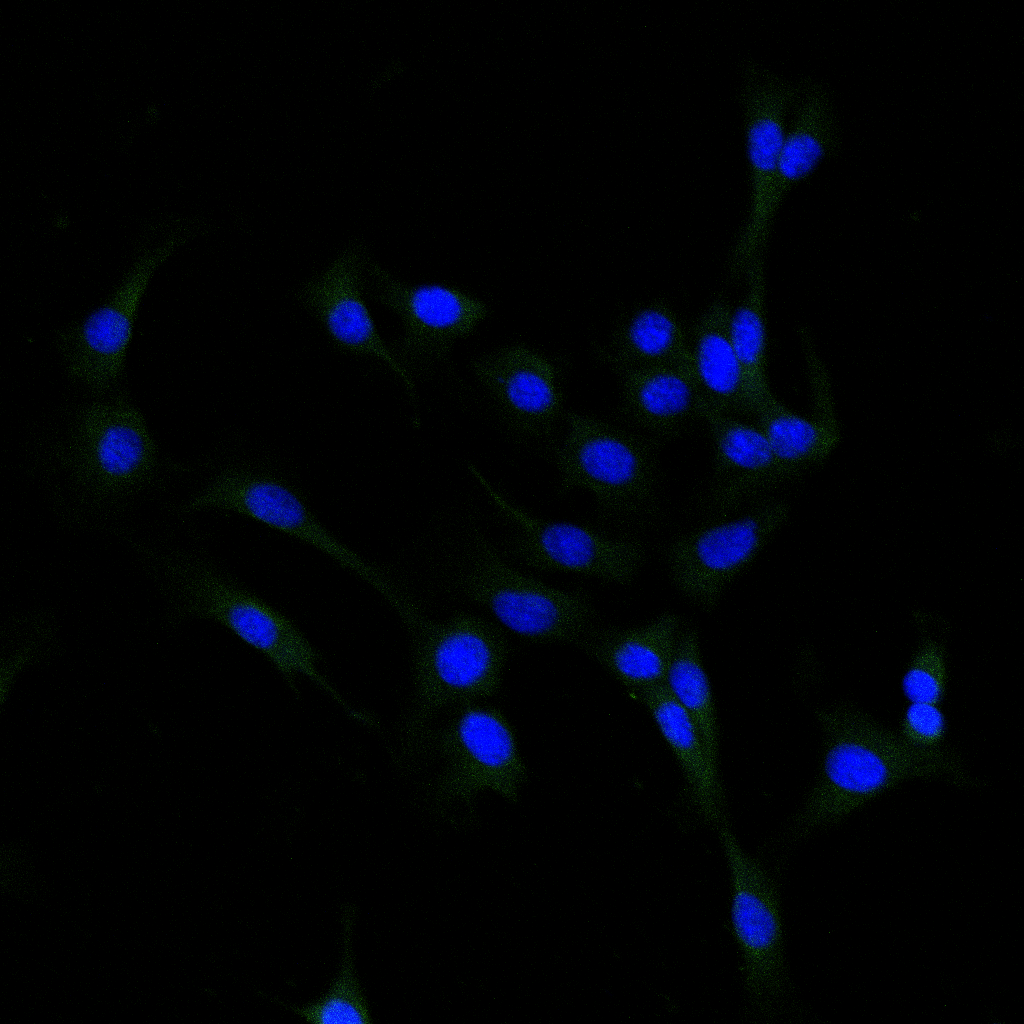

Supplement: Supplementary file 1 [file DataSheet3.ZIP › original files for Fig.3/Fig.3C 3kPa (+)PD Merged.tif]

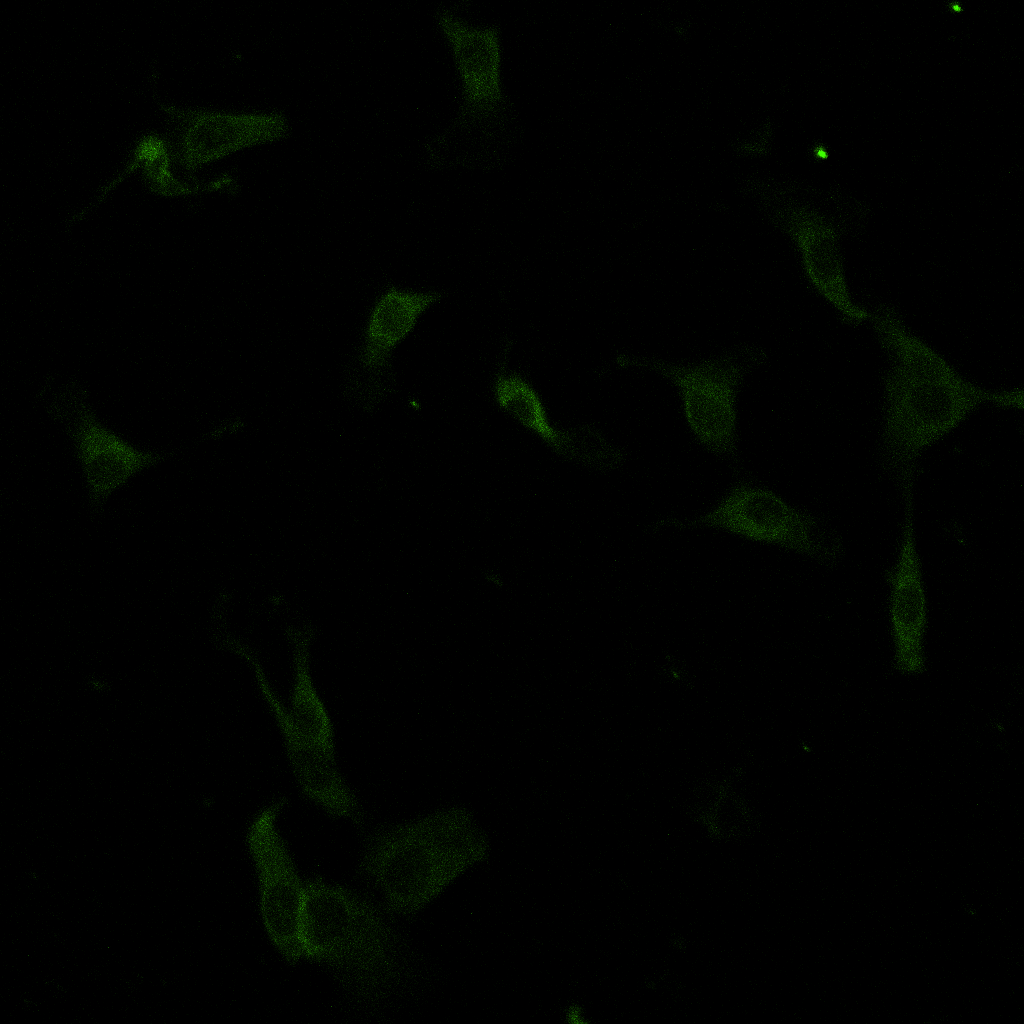

Supplement: Supplementary file 1 [file DataSheet3.ZIP › original files for Fig.3/Fig.3C 3kPa (-)PD Col I .tif]

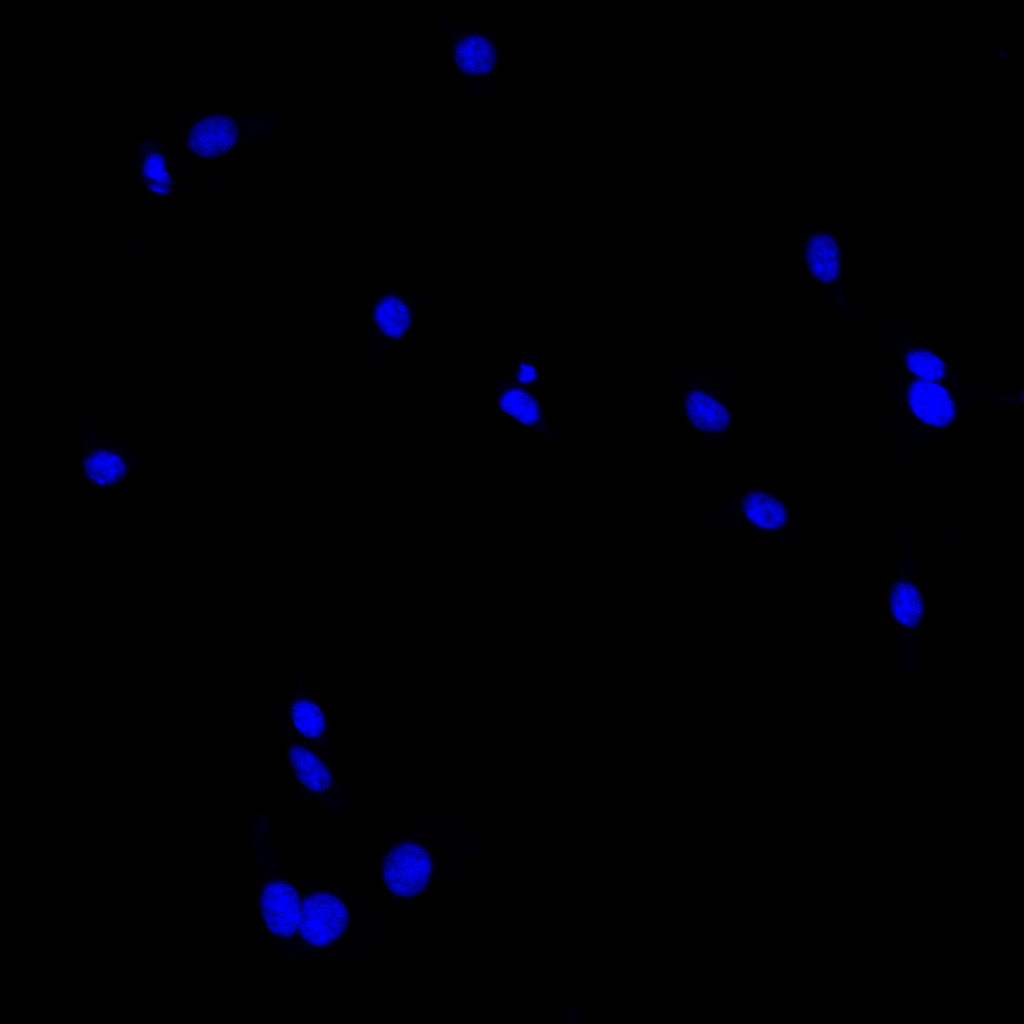

Supplement: Supplementary file 1 [file DataSheet3.ZIP › original files for Fig.3/Fig.3C 3kPa (-)PD DAPI.tif]

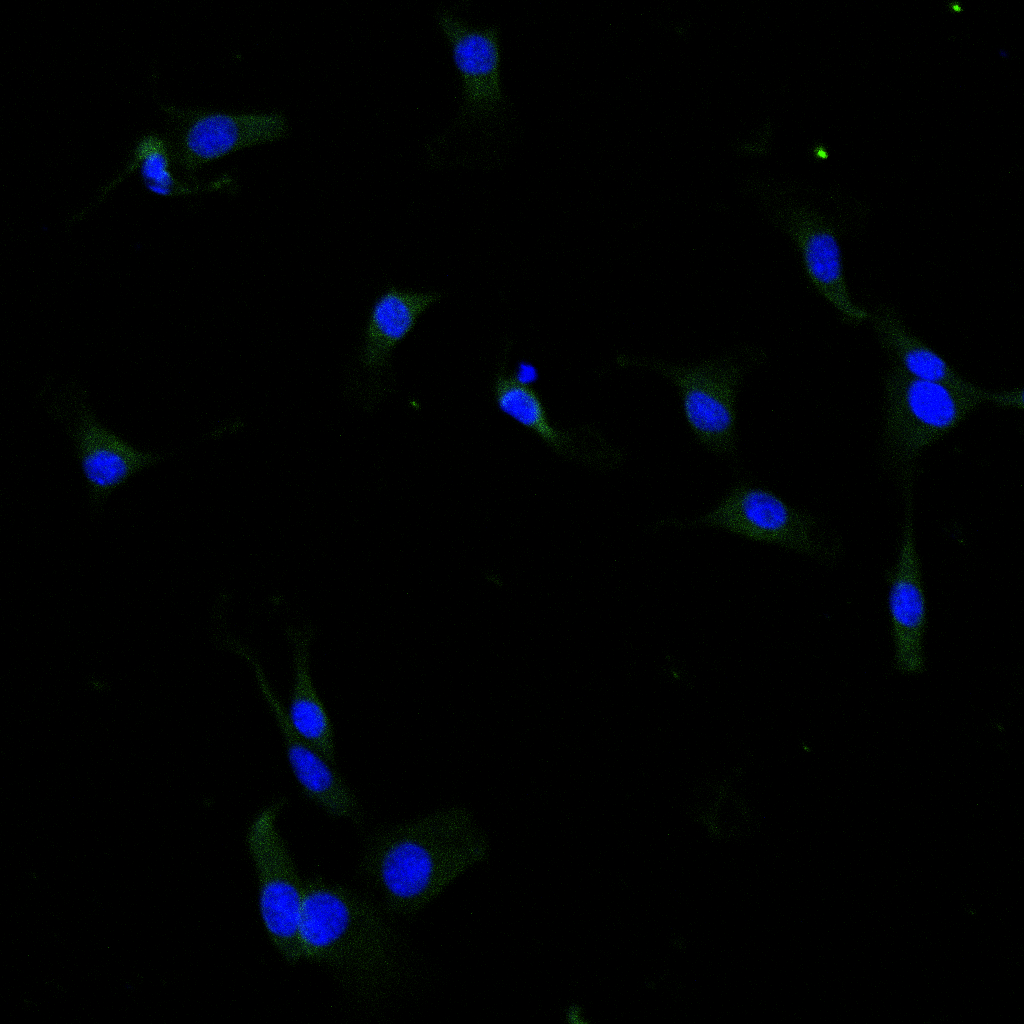

Supplement: Supplementary file 1 [file DataSheet3.ZIP › original files for Fig.3/Fig.3C 3kPa (-)PD Merged.tif]

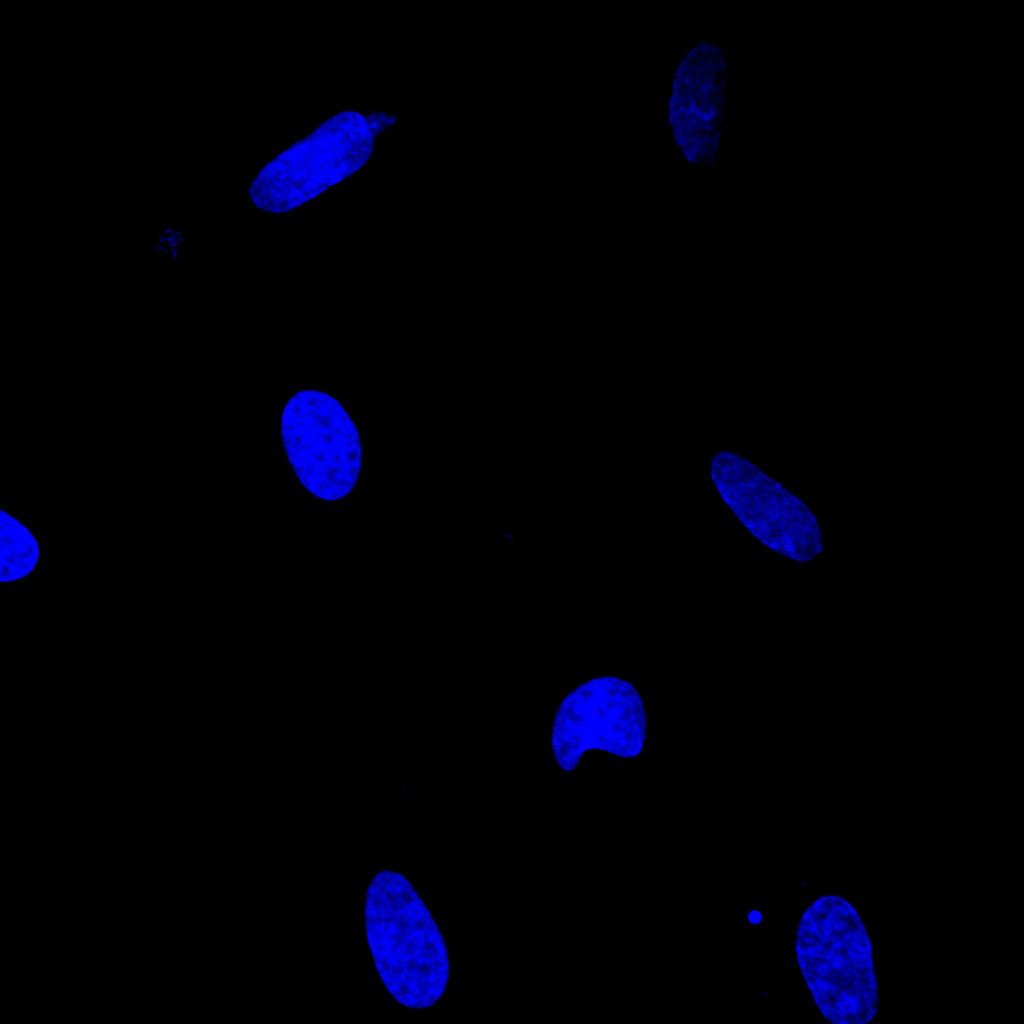

Supplement: Supplementary file 2 [file DataSheet4.ZIP › original files for Fig.4/Fig.4A 12kPa (+)PD DAPI.tif]

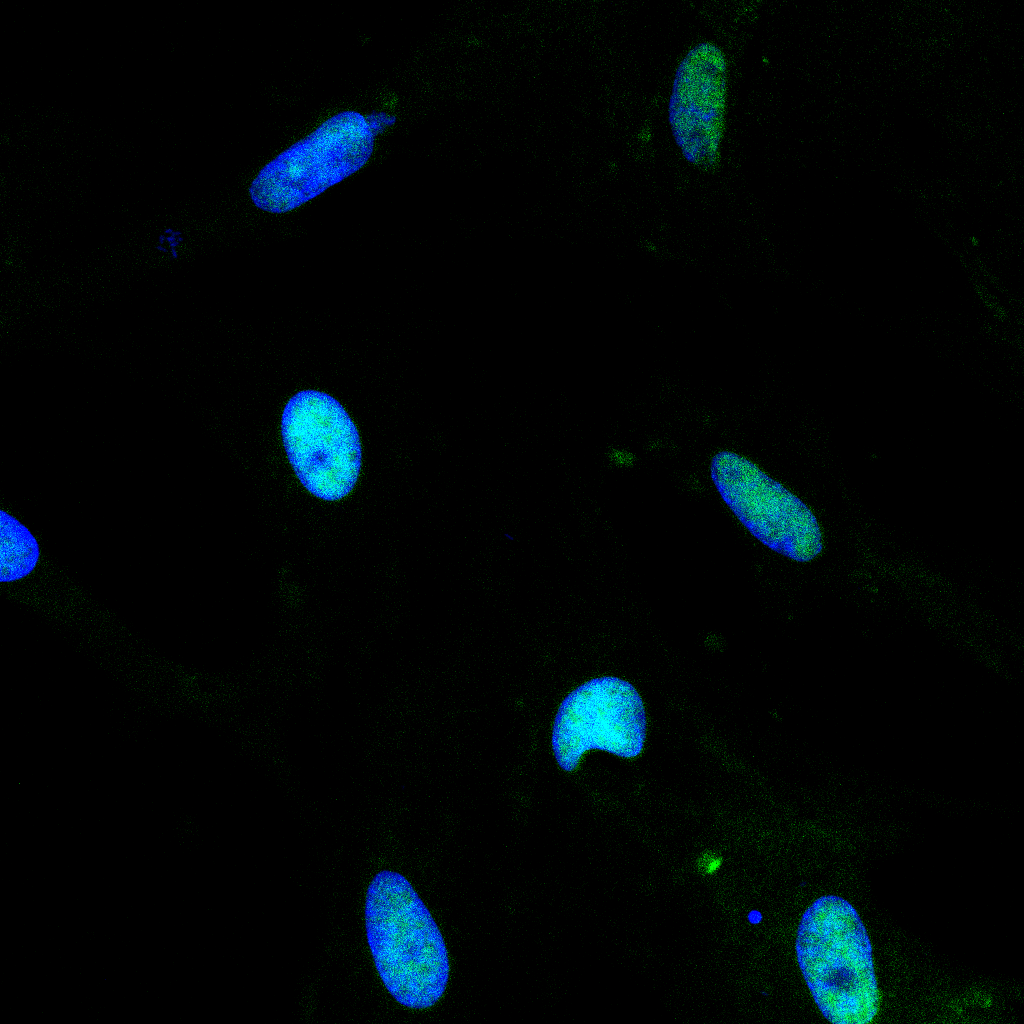

Supplement: Supplementary file 2 [file DataSheet4.ZIP › original files for Fig.4/Fig.4A 12kPa (+)PD Merged.tif]

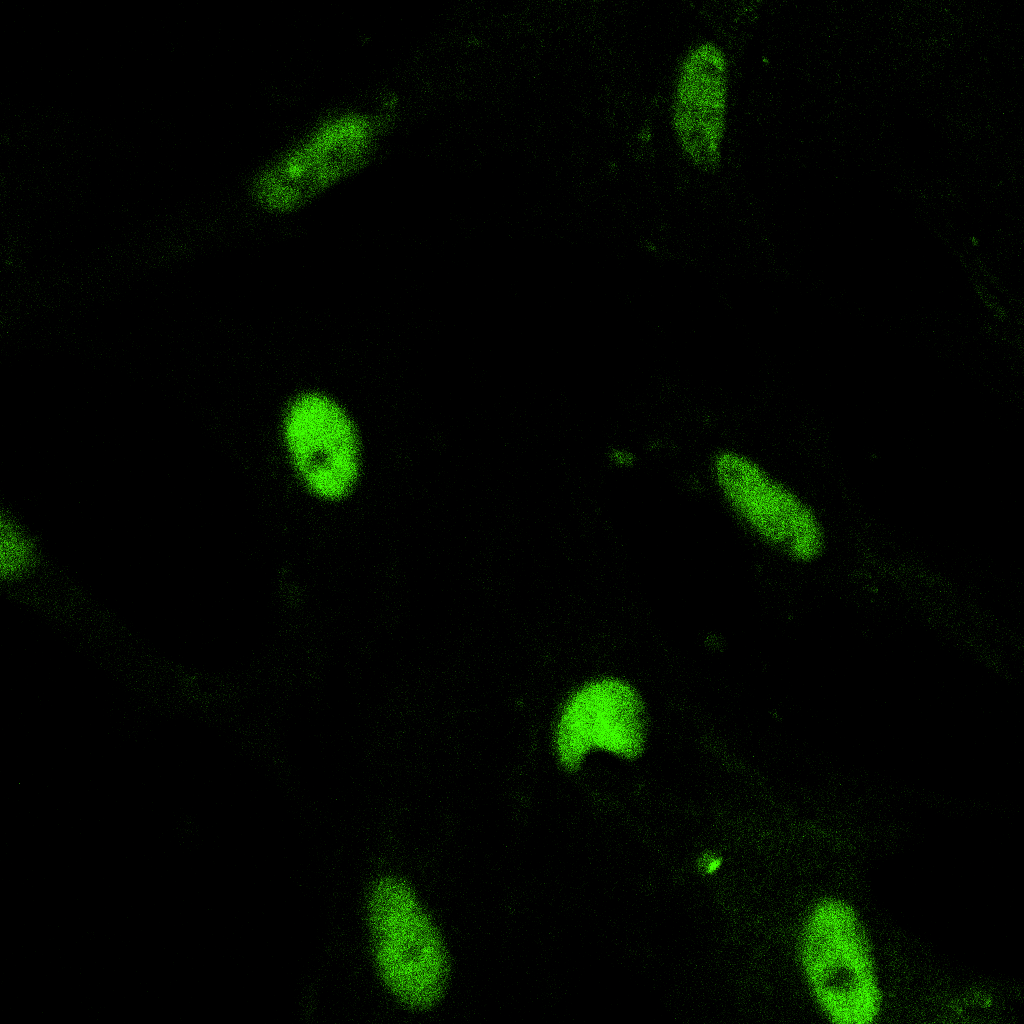

Supplement: Supplementary file 2 [file DataSheet4.ZIP › original files for Fig.4/Fig.4A 12kPa (+)PD YAP.tif]

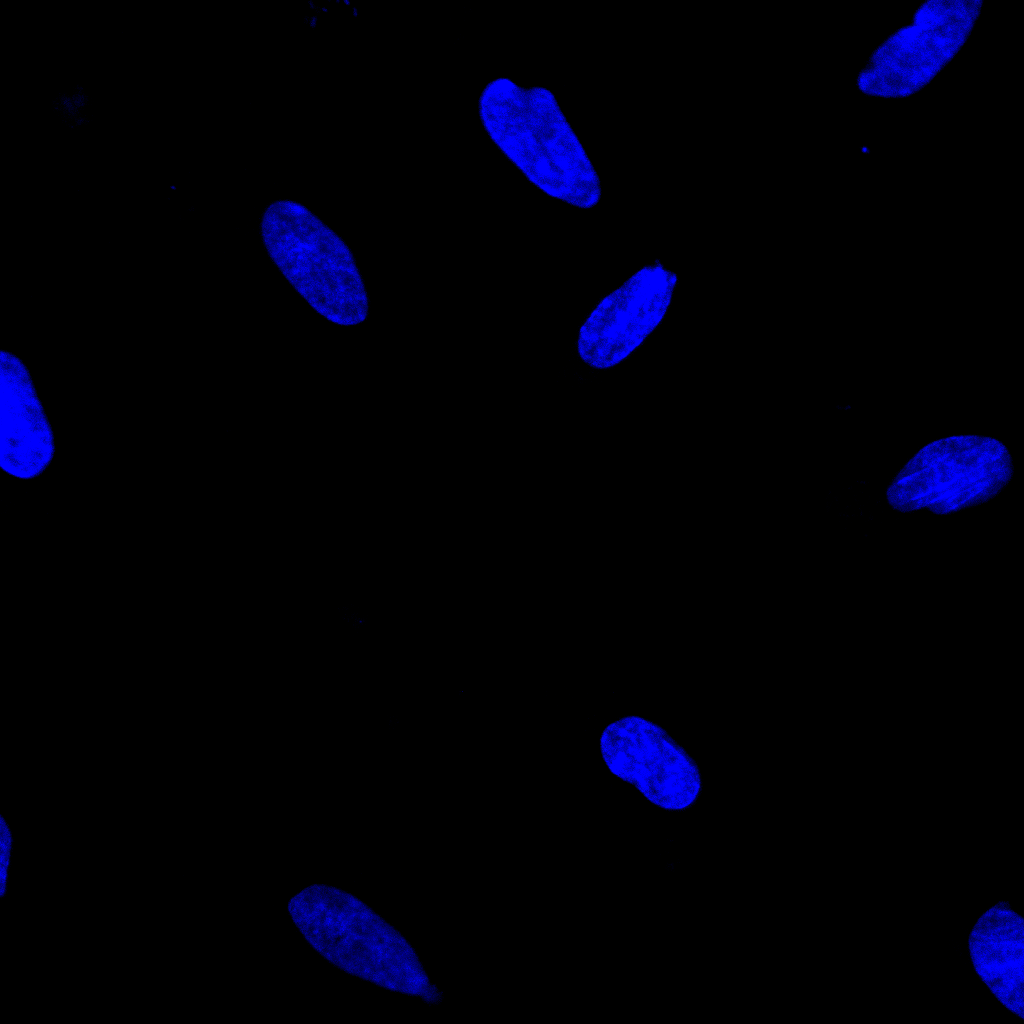

Supplement: Supplementary file 2 [file DataSheet4.ZIP › original files for Fig.4/Fig.4A 12kPa (-)PD DAPI.tif]

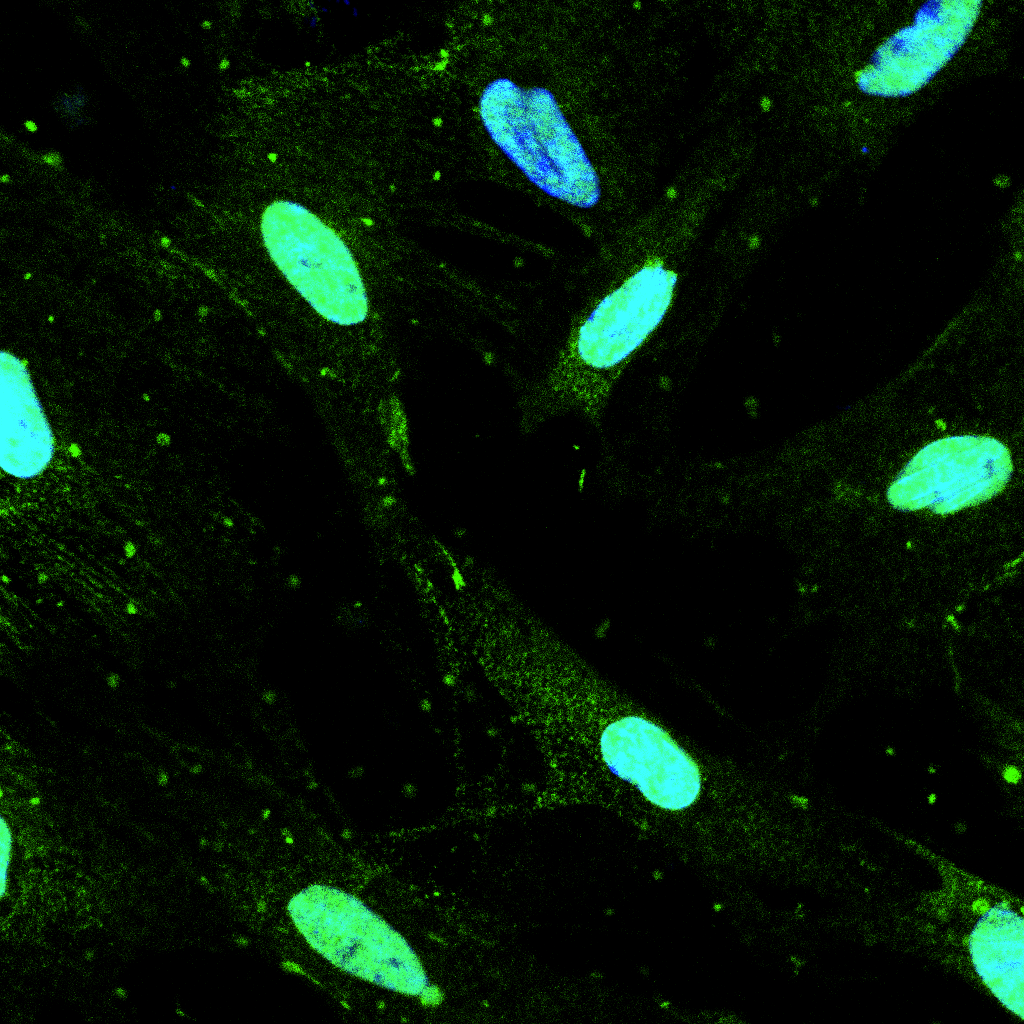

Supplement: Supplementary file 2 [file DataSheet4.ZIP › original files for Fig.4/Fig.4A 12kPa (-)PD Merged.tif]

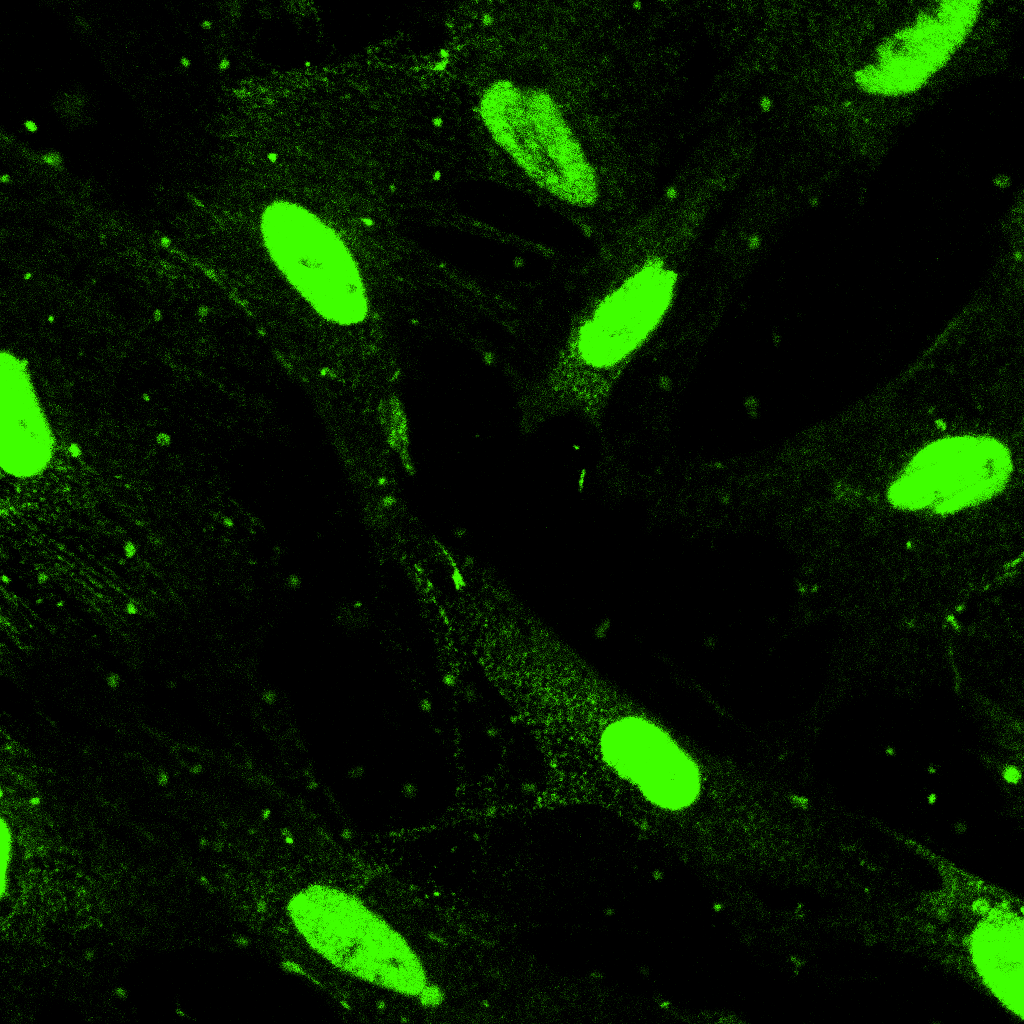

Supplement: Supplementary file 2 [file DataSheet4.ZIP › original files for Fig.4/Fig.4A 12kPa (-)PD YAP.tif]

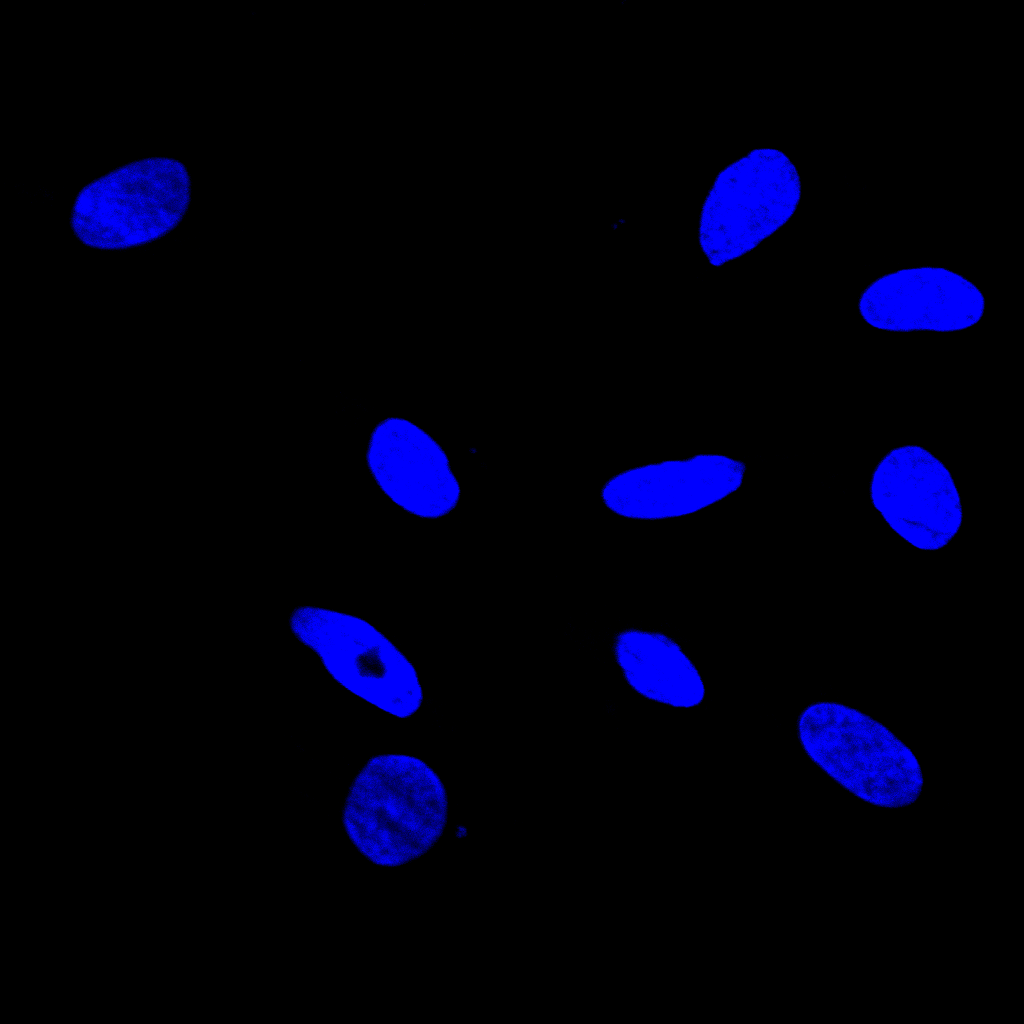

Supplement: Supplementary file 2 [file DataSheet4.ZIP › original files for Fig.4/Fig.4A 30kPa (+)PD DAPI.tif]

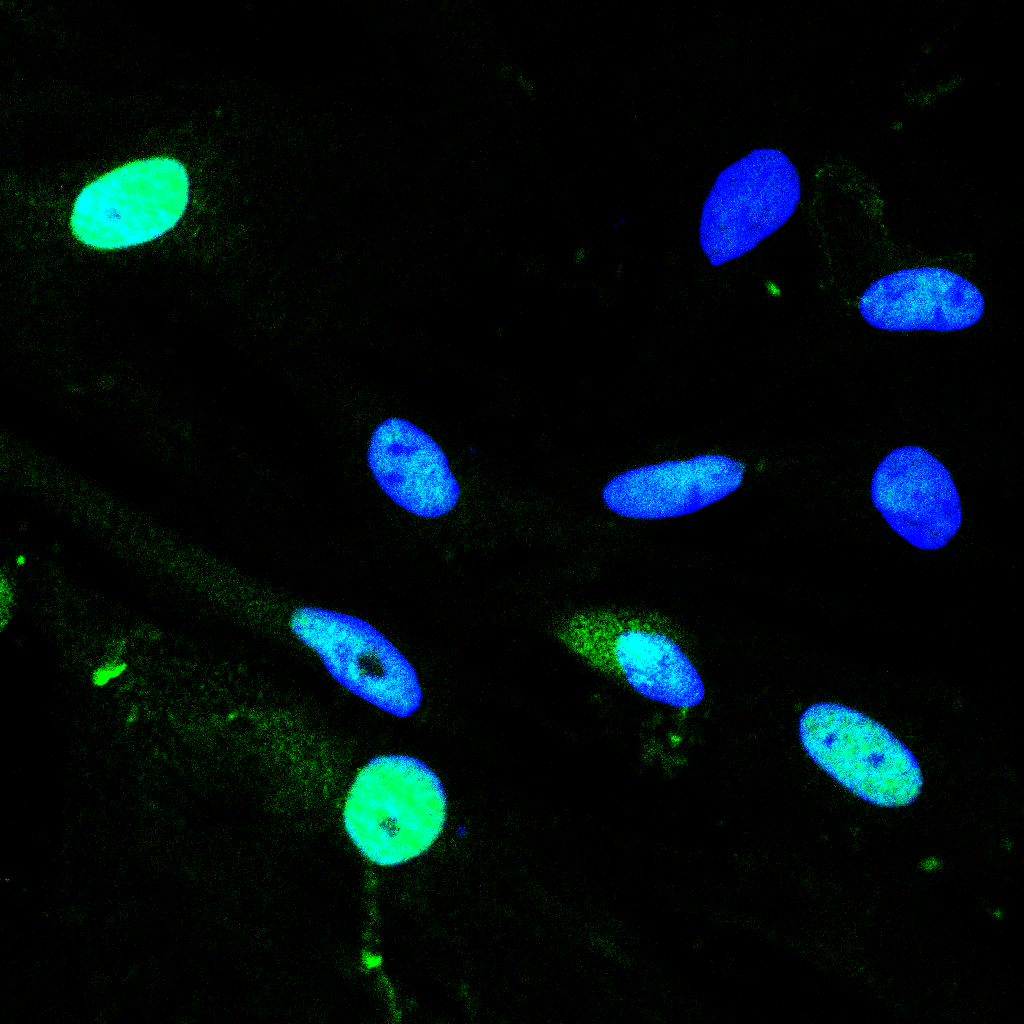

Supplement: Supplementary file 2 [file DataSheet4.ZIP › original files for Fig.4/Fig.4A 30kPa (+)PD Merged.tif]

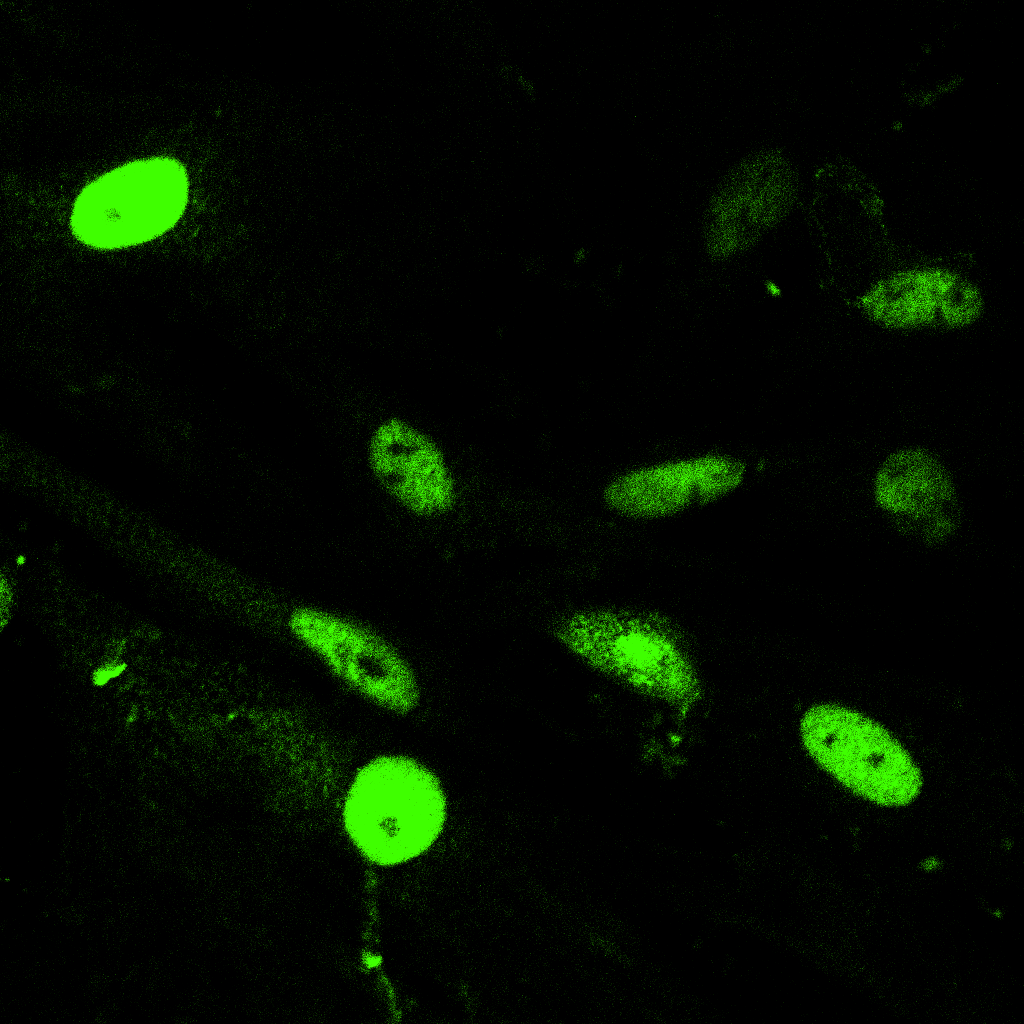

Supplement: Supplementary file 2 [file DataSheet4.ZIP › original files for Fig.4/Fig.4A 30kPa (+)PD YAP.tif]

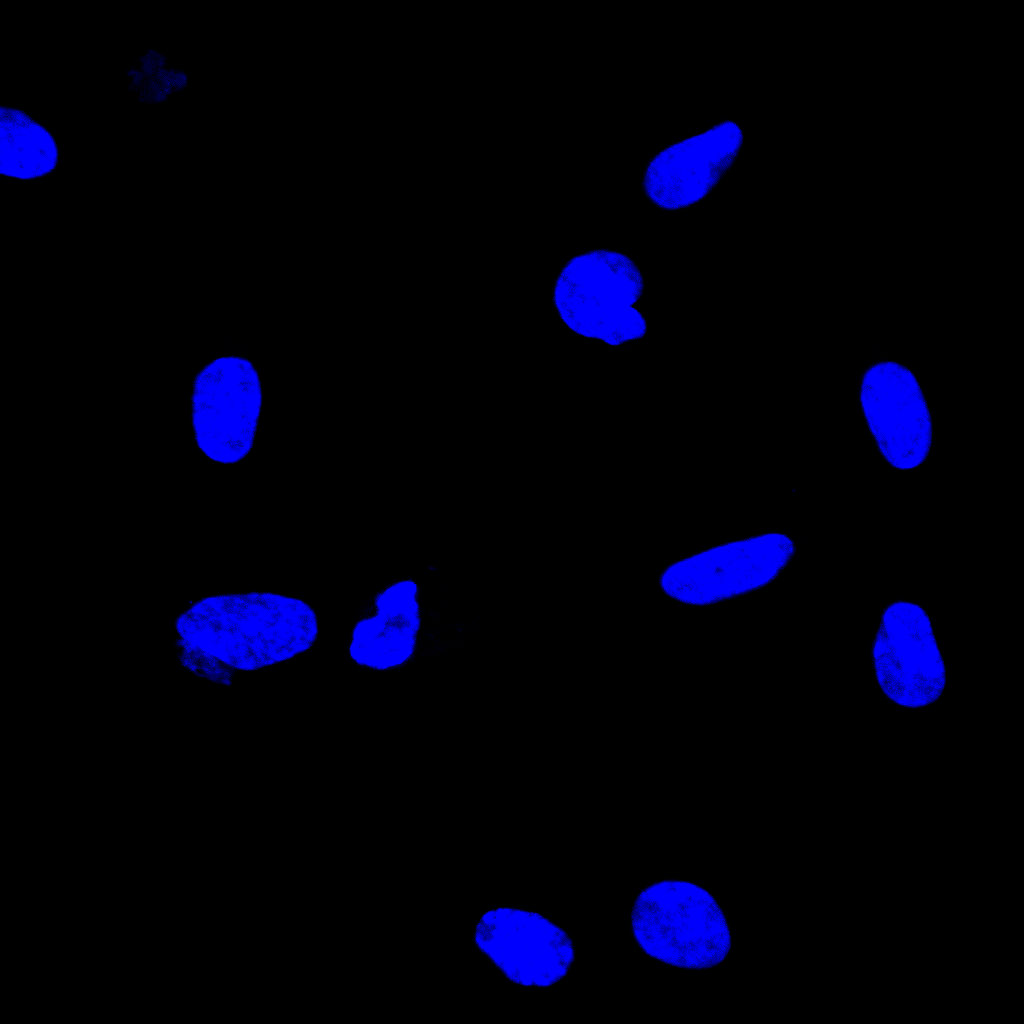

Supplement: Supplementary file 2 [file DataSheet4.ZIP › original files for Fig.4/Fig.4A 30kPa (-)PD DAPI.tif]

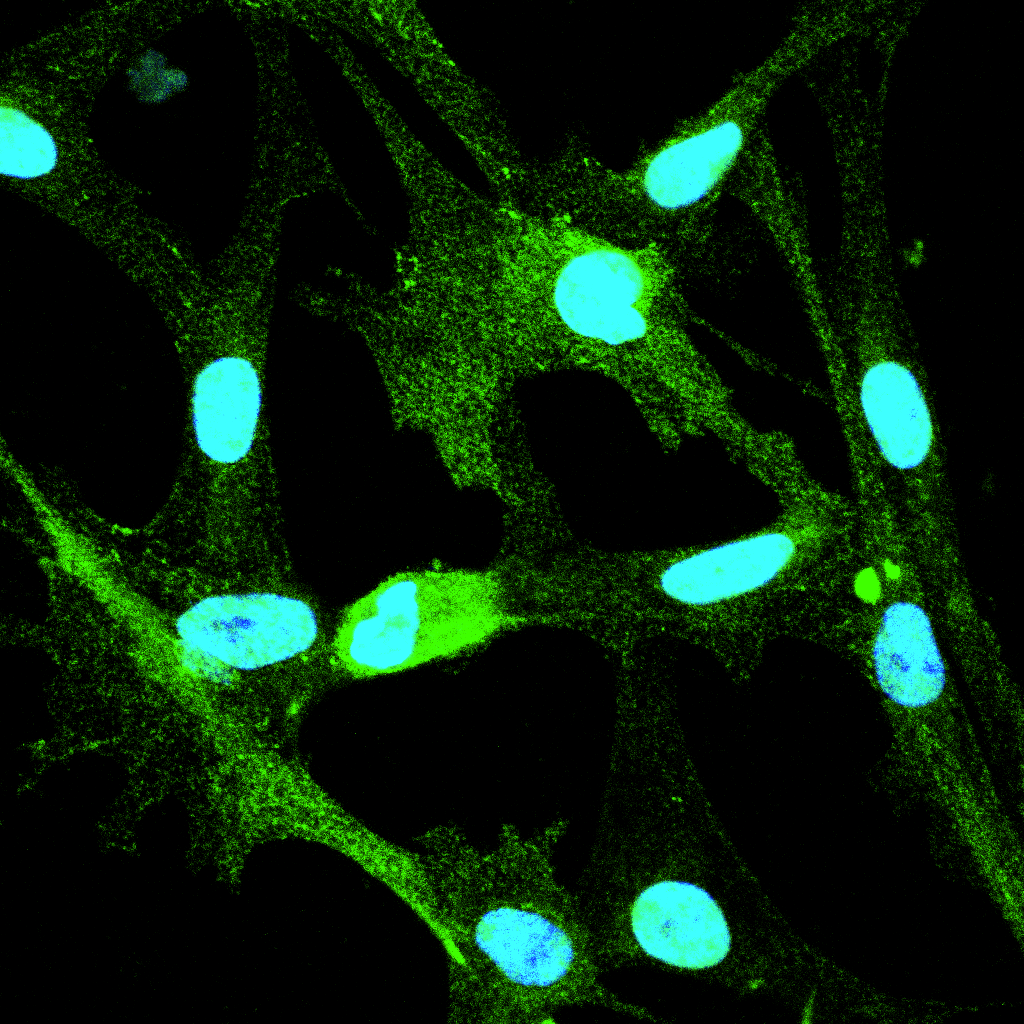

Supplement: Supplementary file 2 [file DataSheet4.ZIP › original files for Fig.4/Fig.4A 30kPa (-)PD Merged.tif]

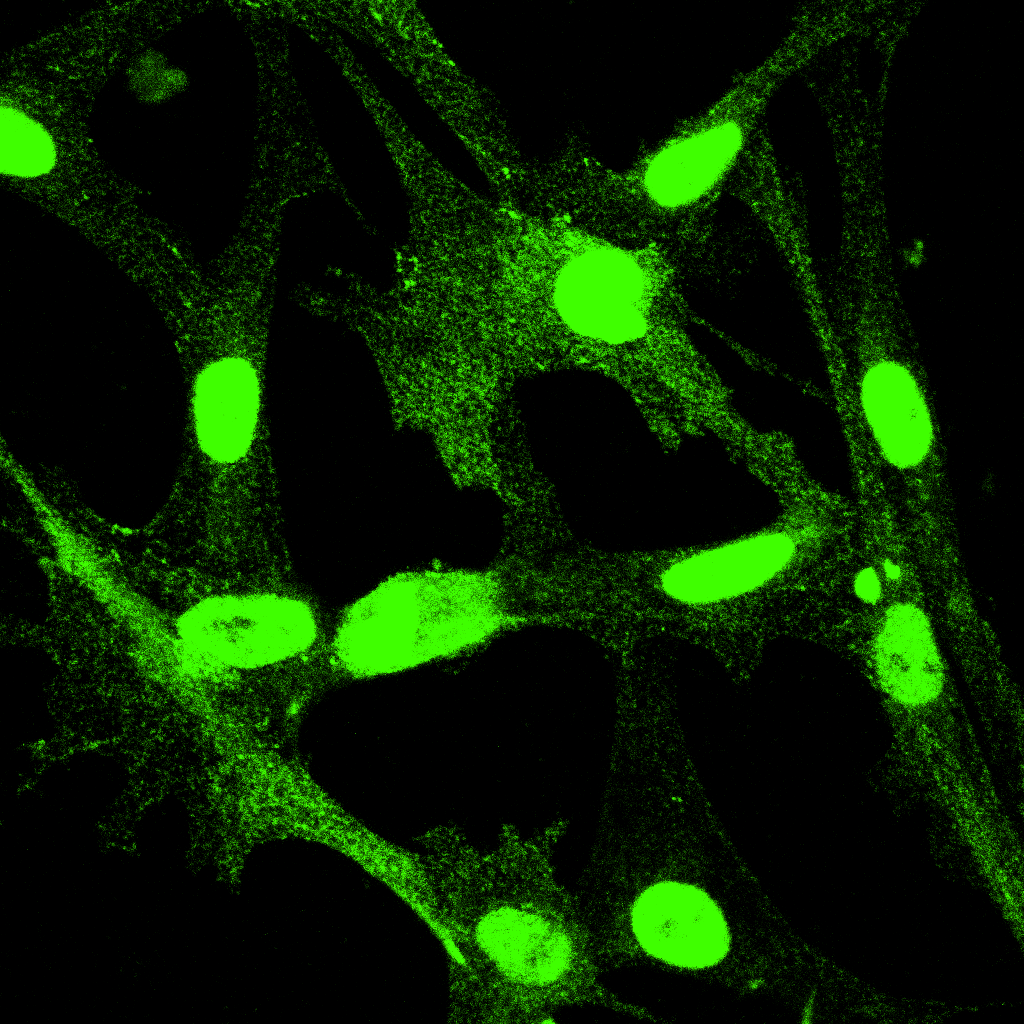

Supplement: Supplementary file 2 [file DataSheet4.ZIP › original files for Fig.4/Fig.4A 30kPa (-)PD YAP.tif]

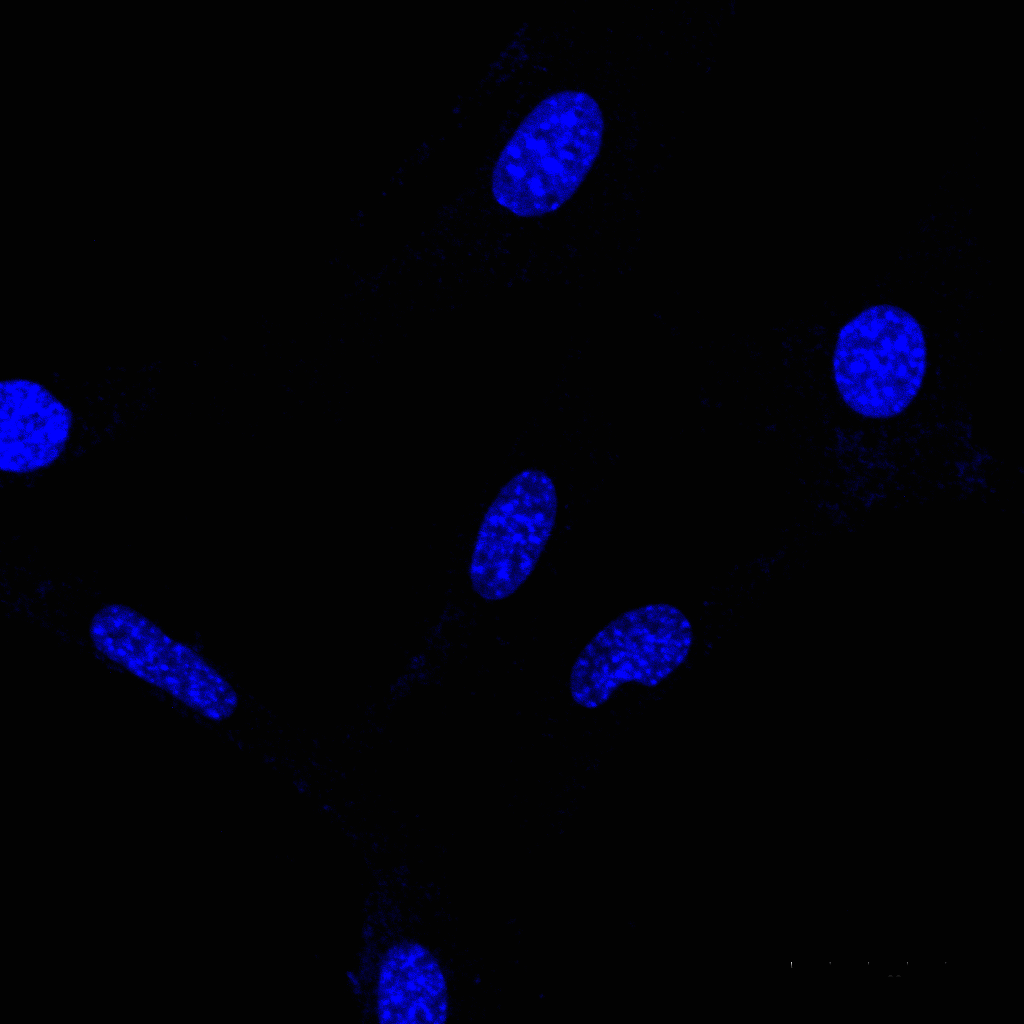

Supplement: Supplementary file 2 [file DataSheet4.ZIP › original files for Fig.4/Fig.4A 3kPa (+)PD DAPI.tif]

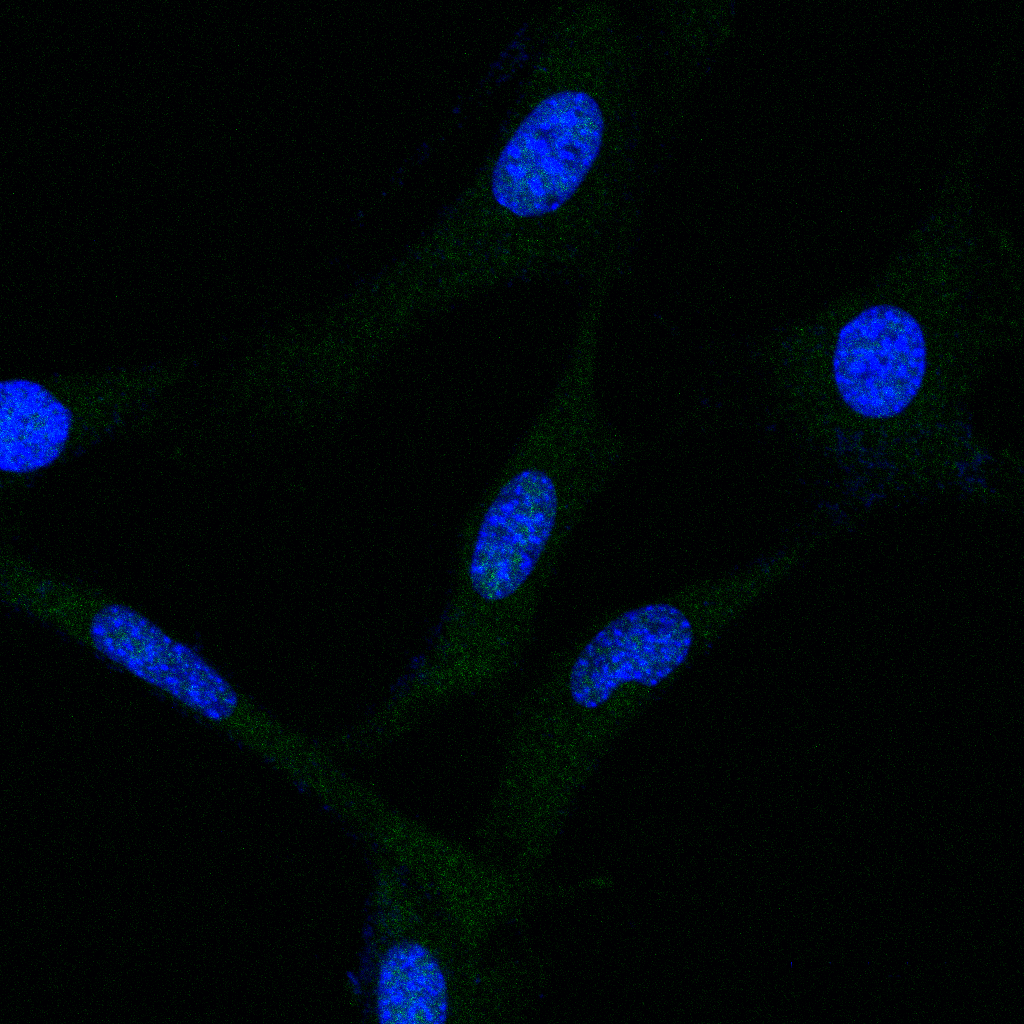

Supplement: Supplementary file 2 [file DataSheet4.ZIP › original files for Fig.4/Fig.4A 3kPa (+)PD Merged.tif]

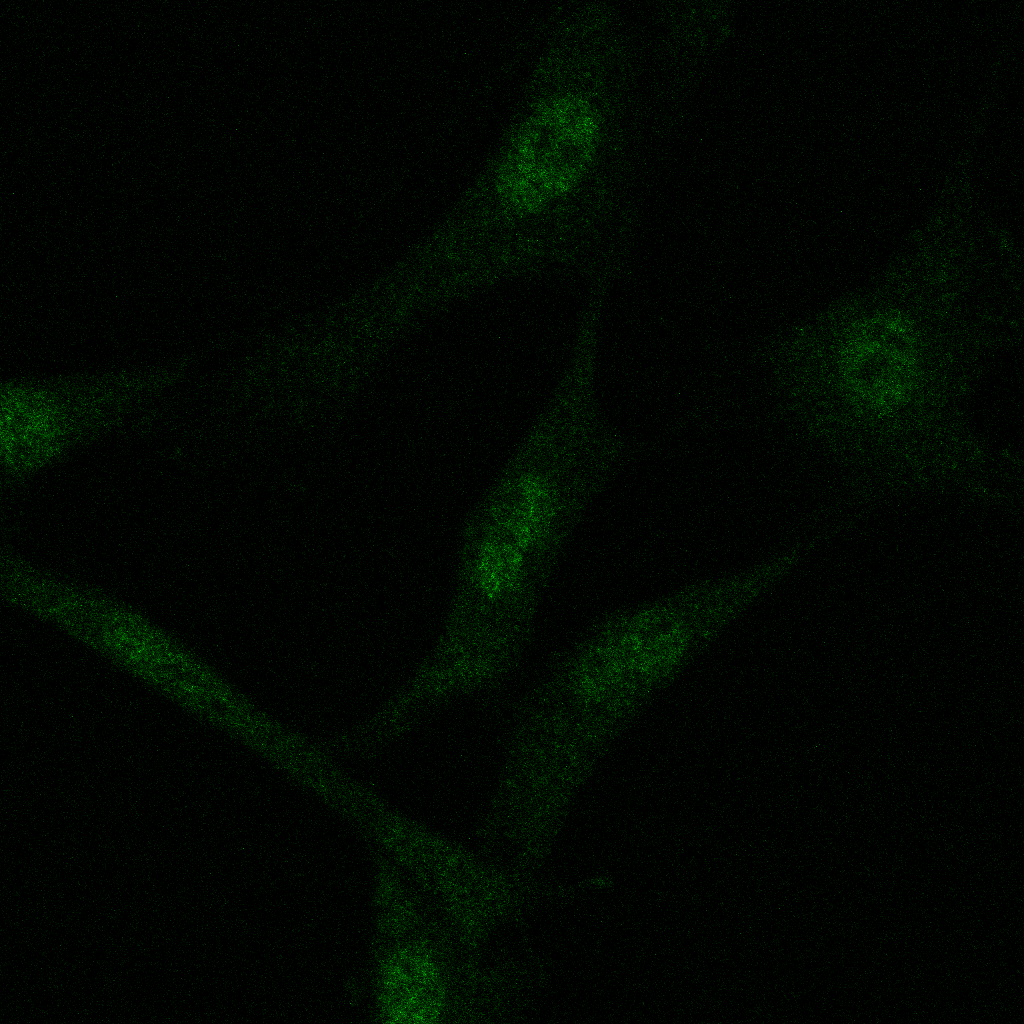

Supplement: Supplementary file 2 [file DataSheet4.ZIP › original files for Fig.4/Fig.4A 3kPa (+)PD YAP.tif]

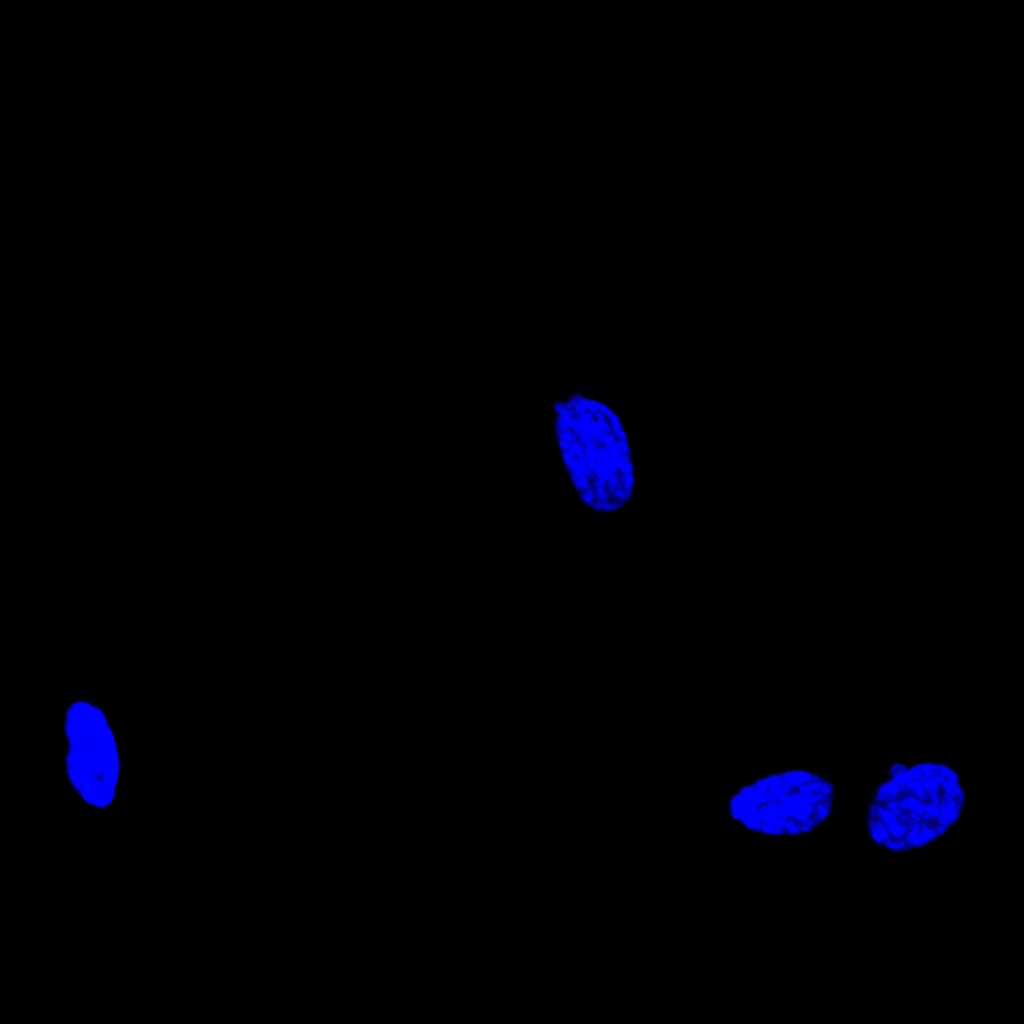

Supplement: Supplementary file 2 [file DataSheet4.ZIP › original files for Fig.4/Fig.4A 3kPa (-)PD DAPI.tif]

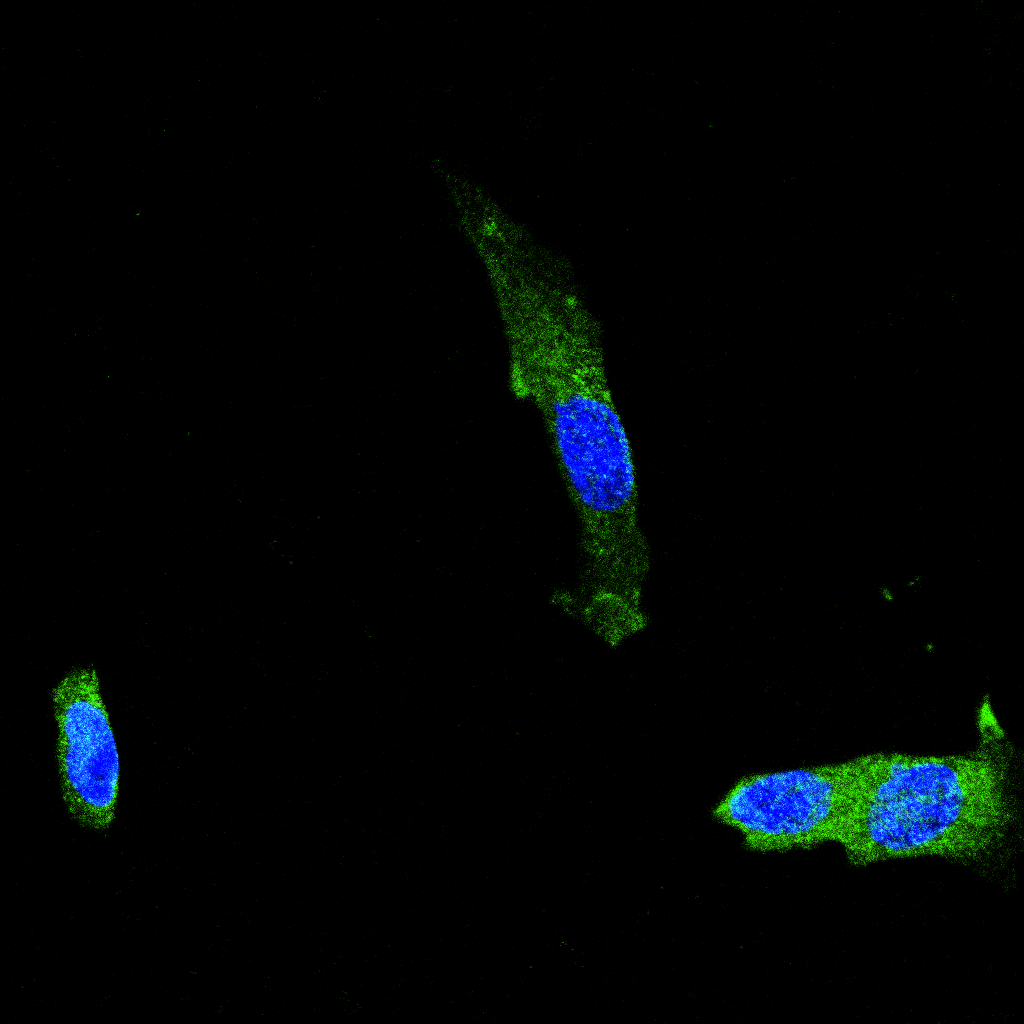

Supplement: Supplementary file 2 [file DataSheet4.ZIP › original files for Fig.4/Fig.4A 3kPa (-)PD Merged.tif]

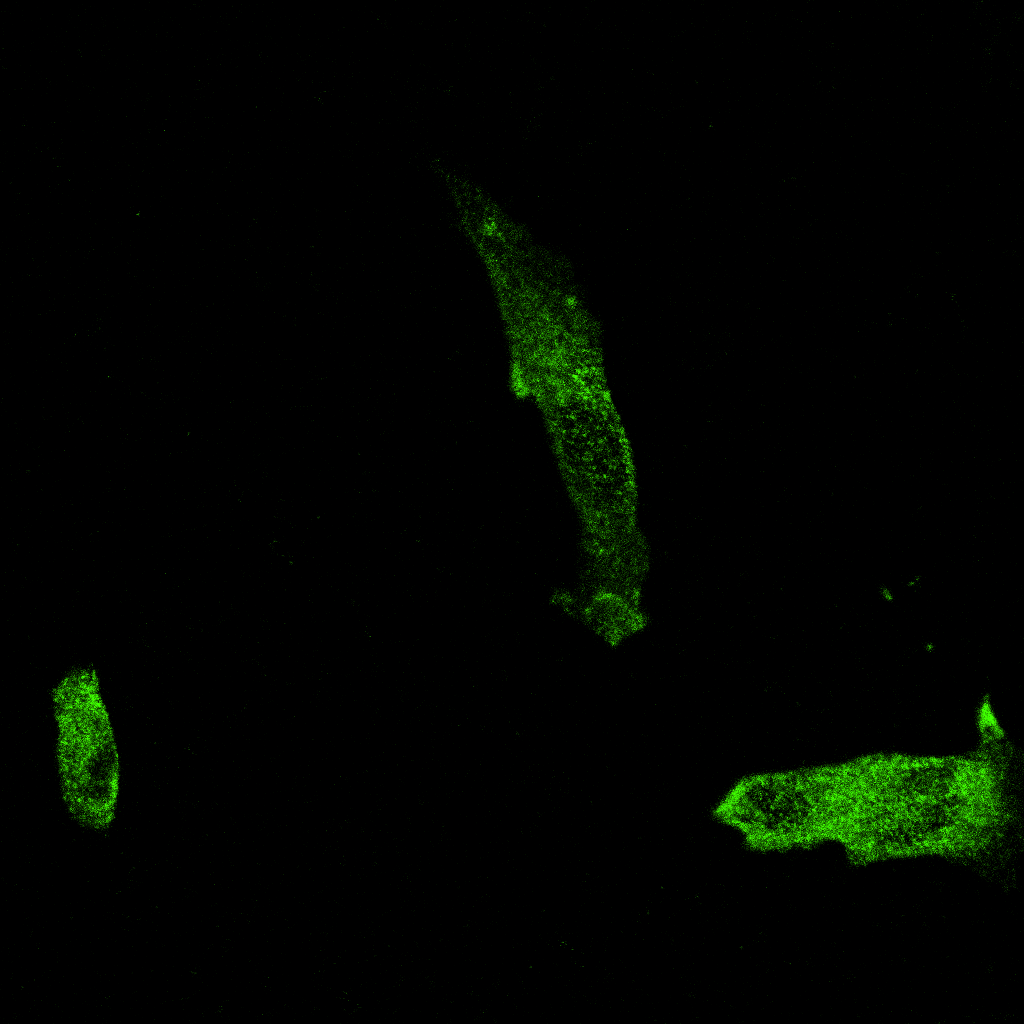

Supplement: Supplementary file 2 [file DataSheet4.ZIP › original files for Fig.4/Fig.4A 3kPa (-)PD YAP.tif]

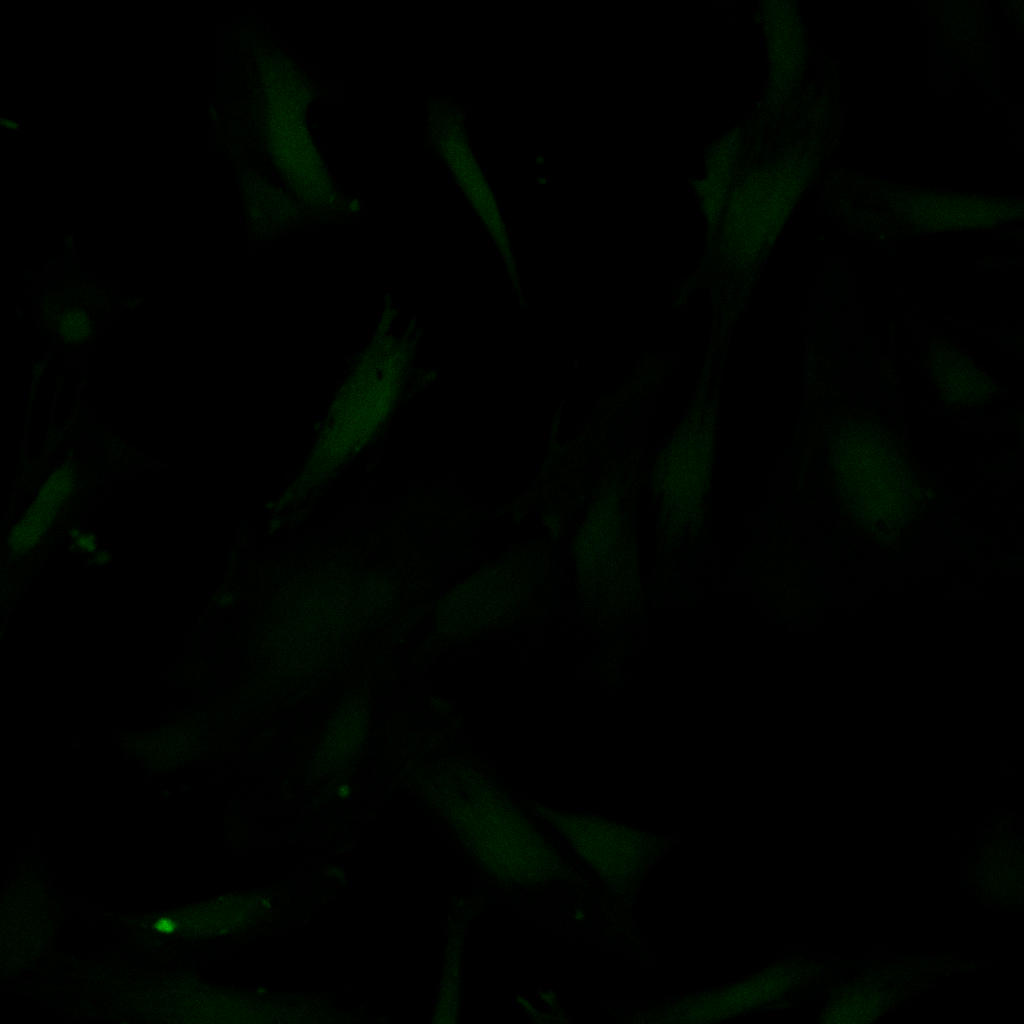

Supplement: Supplementary file 2 [file DataSheet4.ZIP › original files for Fig.4/Fig.4D 12kPa (+)PD ROS.tif]

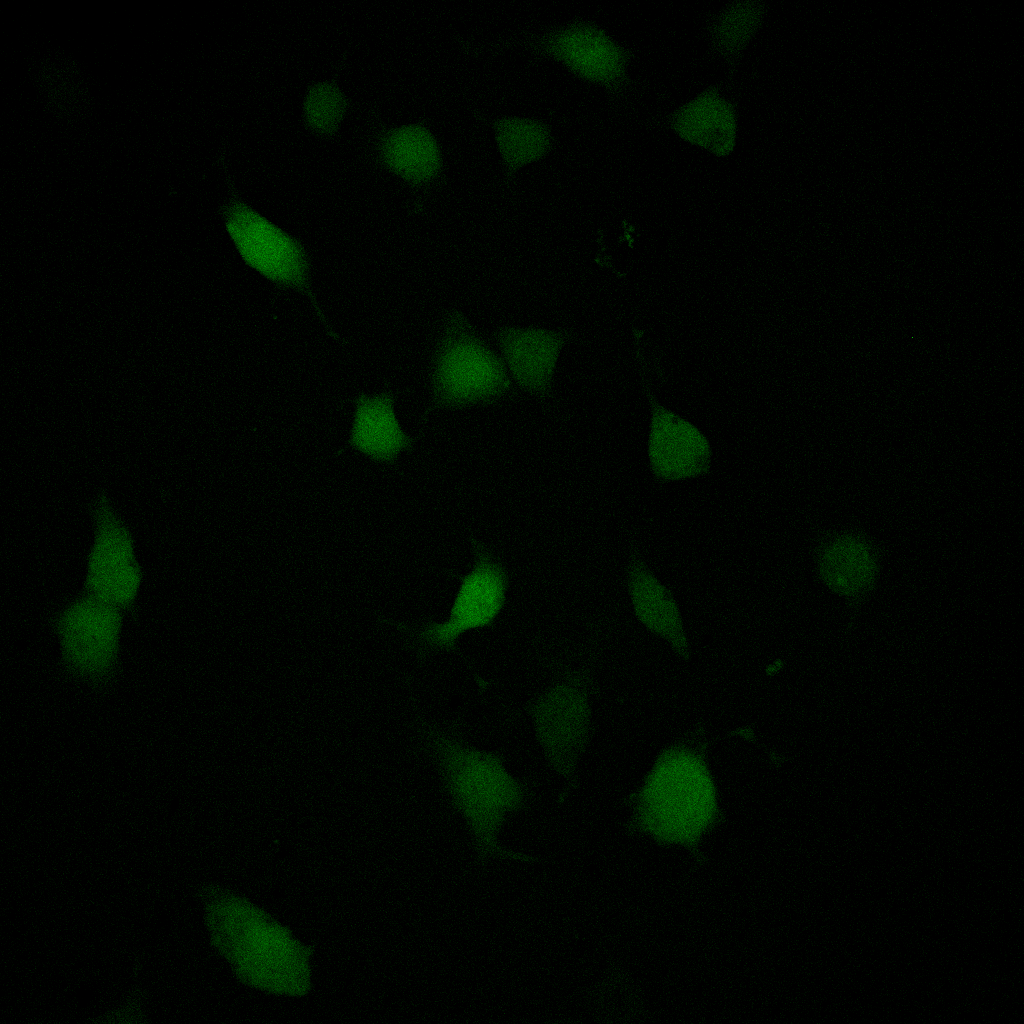

Supplement: Supplementary file 2 [file DataSheet4.ZIP › original files for Fig.4/Fig.4D 12kPa (-)PD ROS.tif]

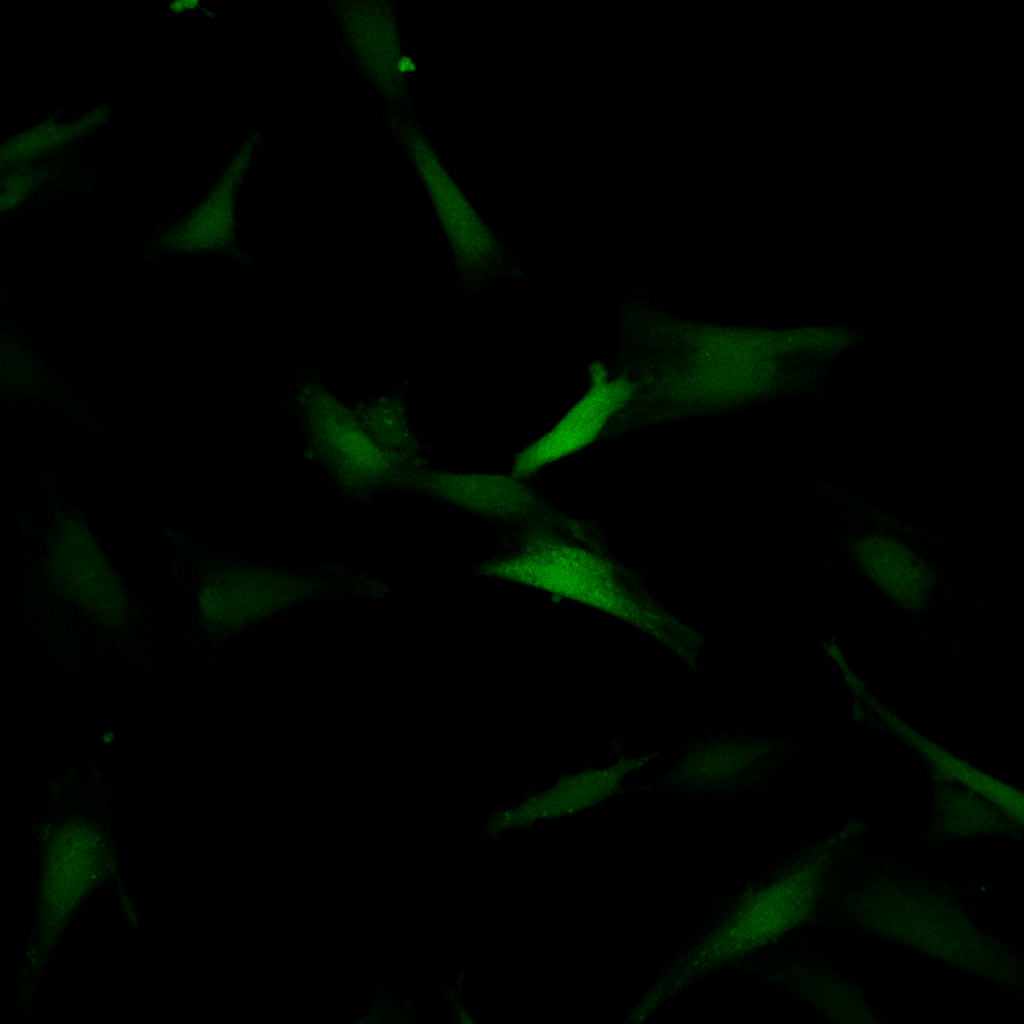

Supplement: Supplementary file 2 [file DataSheet4.ZIP › original files for Fig.4/Fig.4D 30kPa (+)PD ROS.tif]

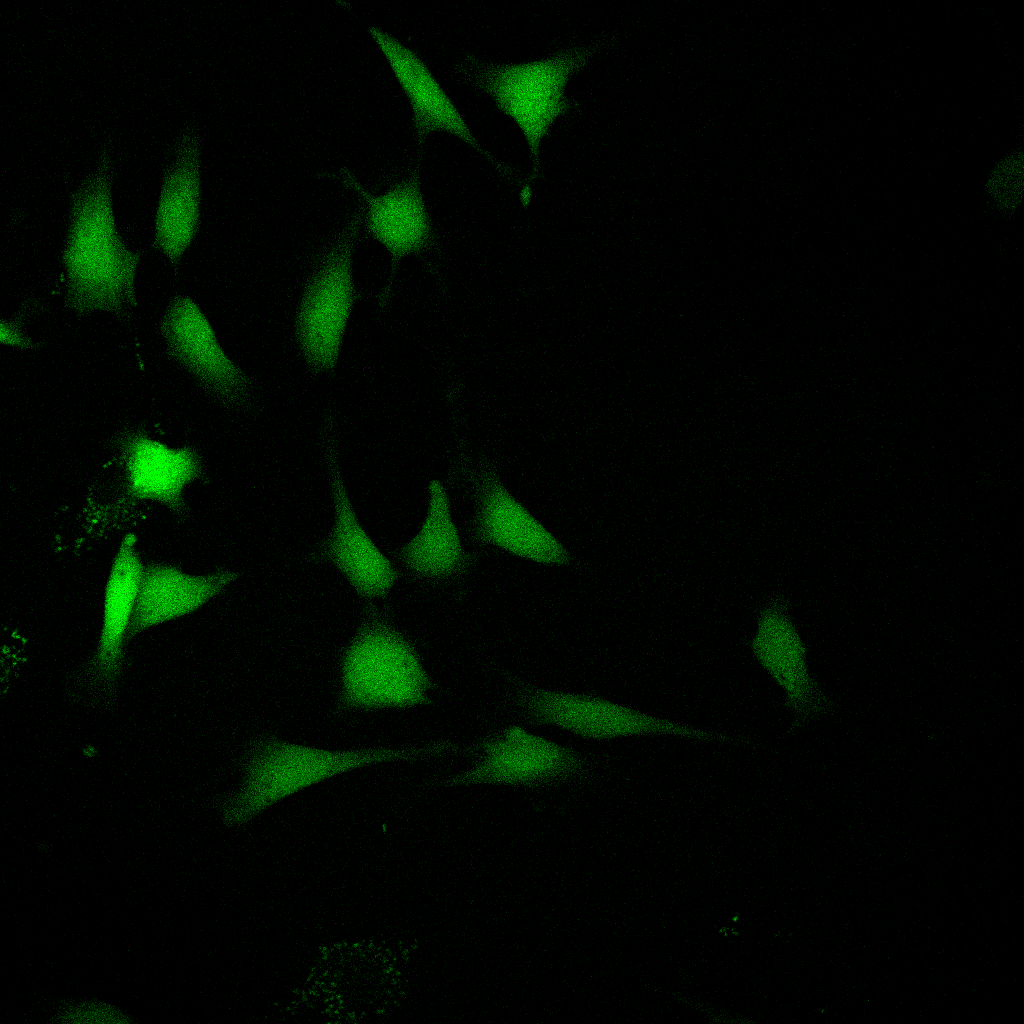

Supplement: Supplementary file 2 [file DataSheet4.ZIP › original files for Fig.4/Fig.4D 30kPa (-)PD ROS.tif]

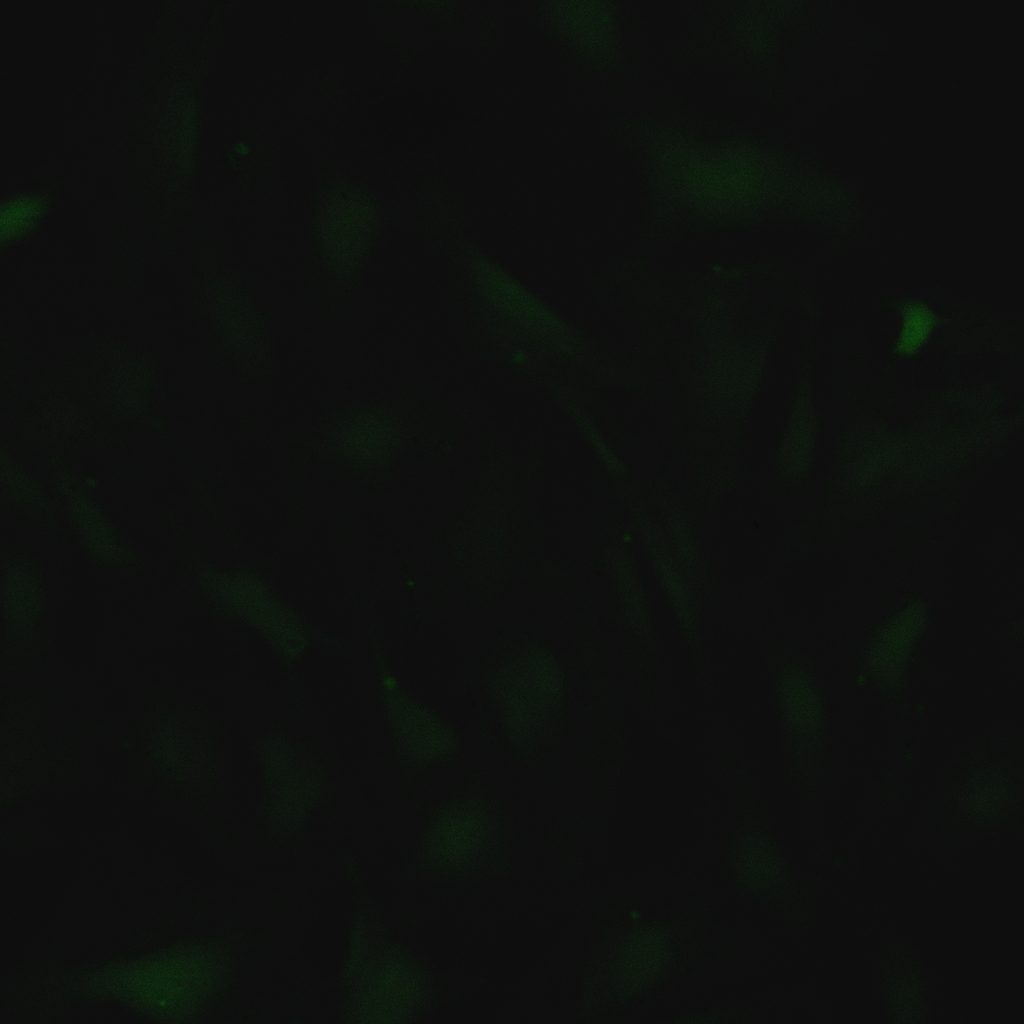

Supplement: Supplementary file 2 [file DataSheet4.ZIP › original files for Fig.4/Fig.4D 3kPa (+)PD ROS.tif]

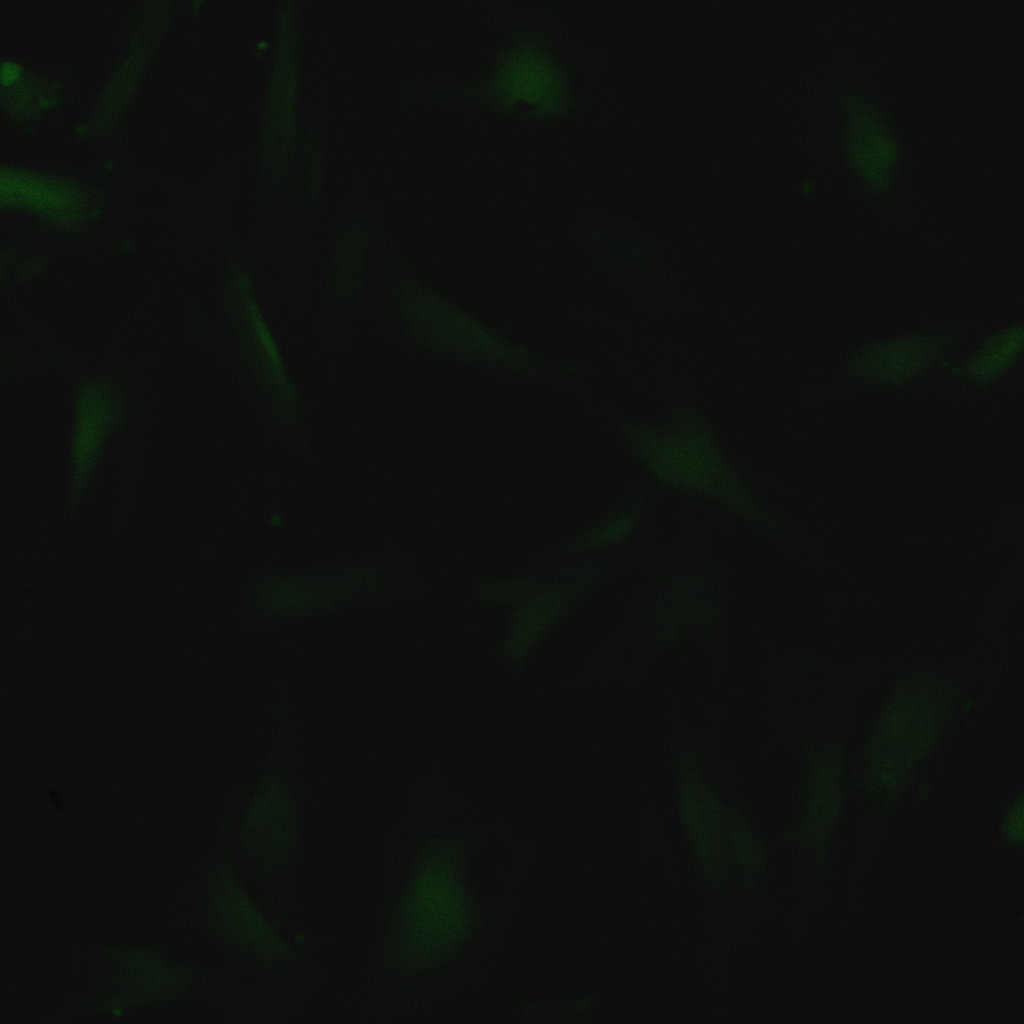

Supplement: Supplementary file 2 [file DataSheet4.ZIP › original files for Fig.4/Fig.4D 3kPa (-)PD ROS.tif]

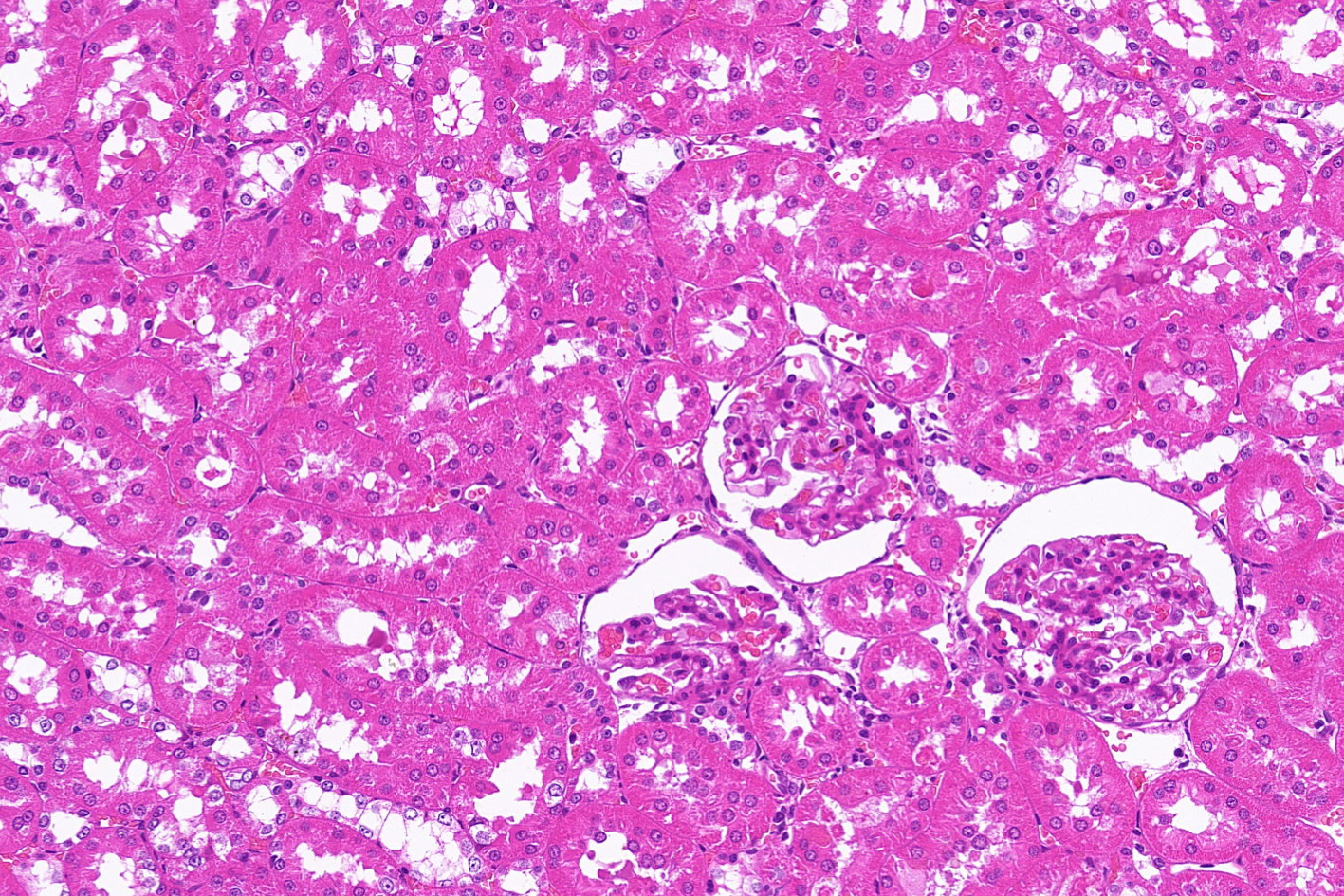

Supplement: Supplementary file 3 [file DataSheet1.ZIP › original files for Fig.1/Fig.1F HE-DN16W.jpg]

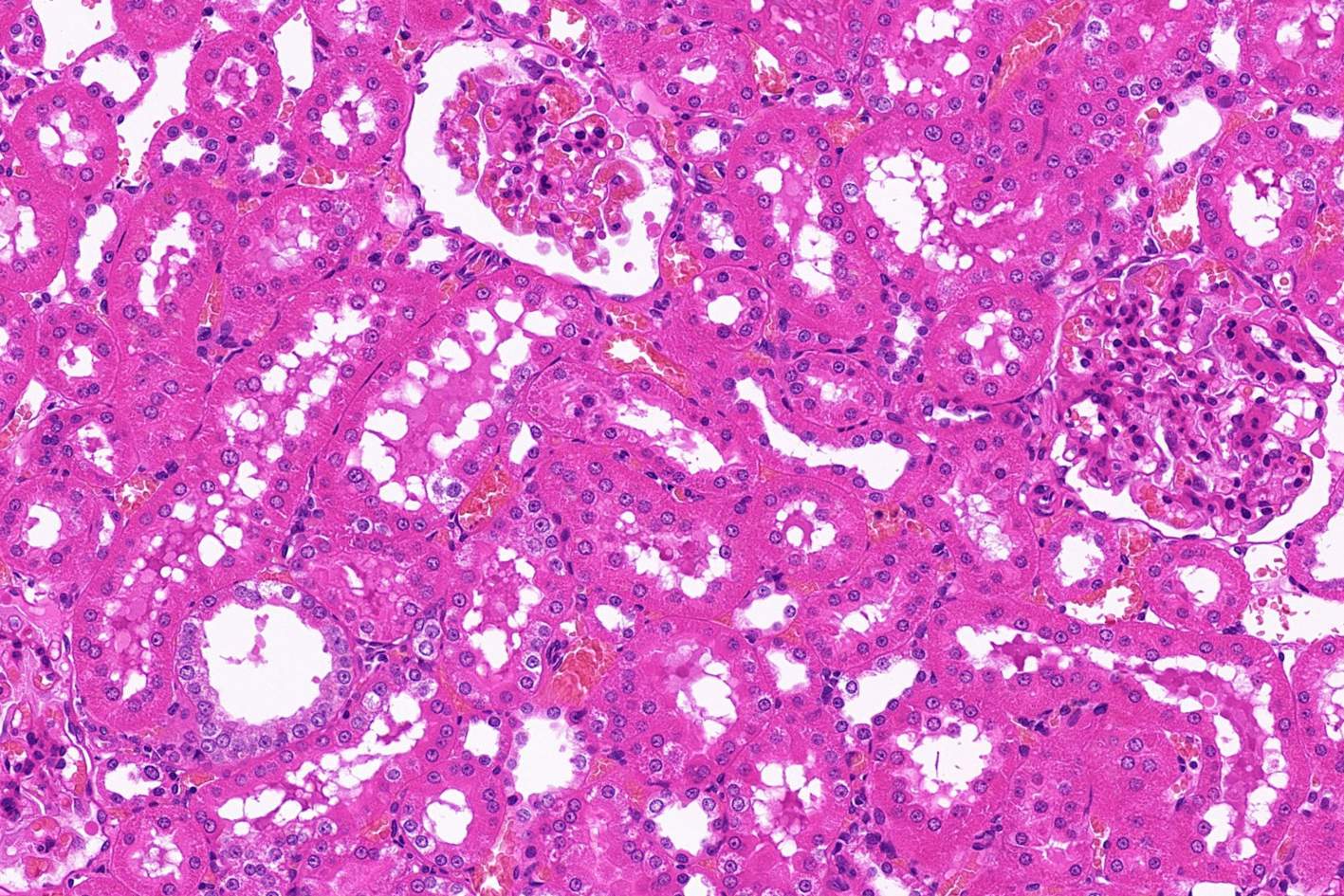

Supplement: Supplementary file 3 [file DataSheet1.ZIP › original files for Fig.1/Fig.1F HE-DN8W.jpg]

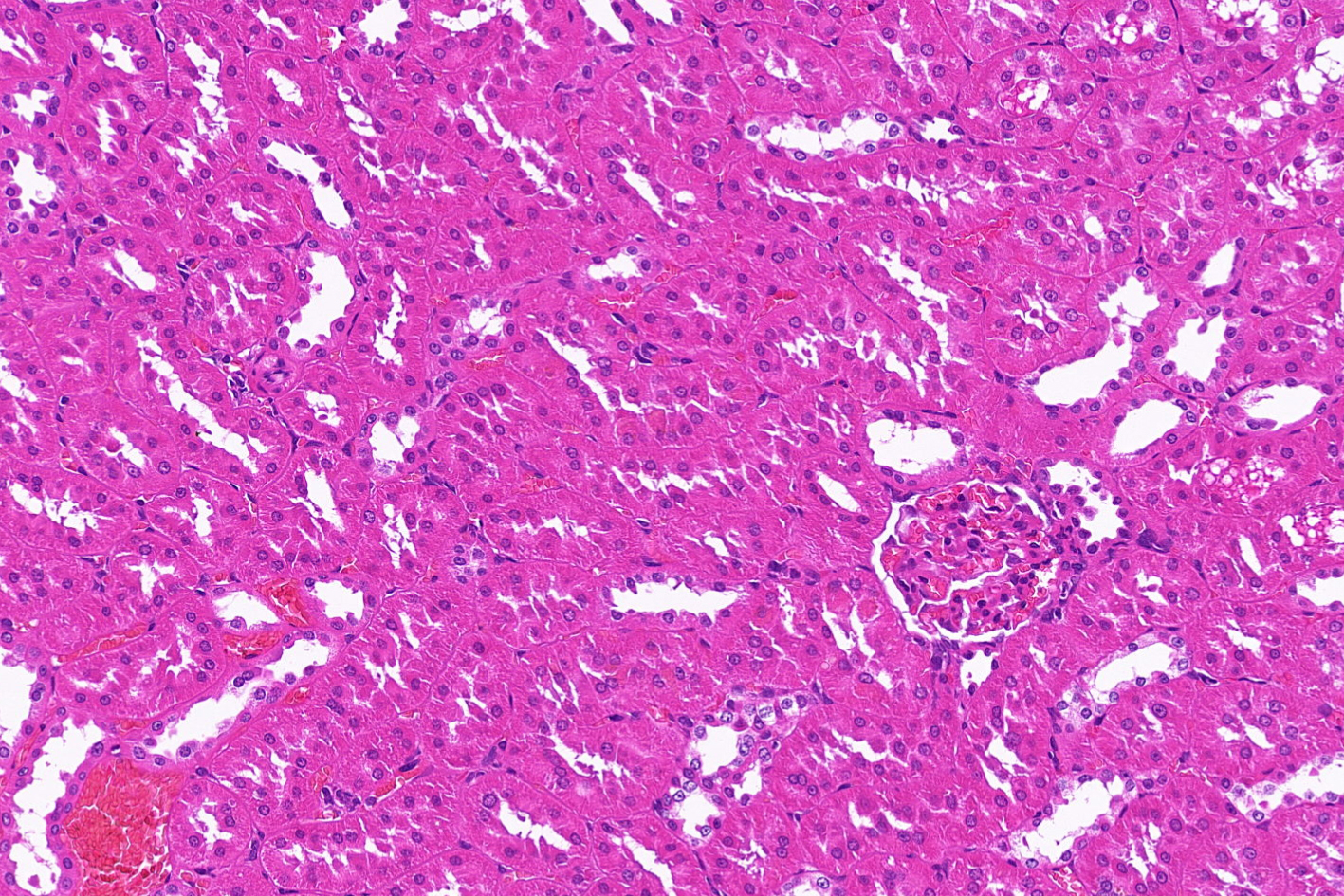

Supplement: Supplementary file 3 [file DataSheet1.ZIP › original files for Fig.1/Fig.1F HE-control.jpg]

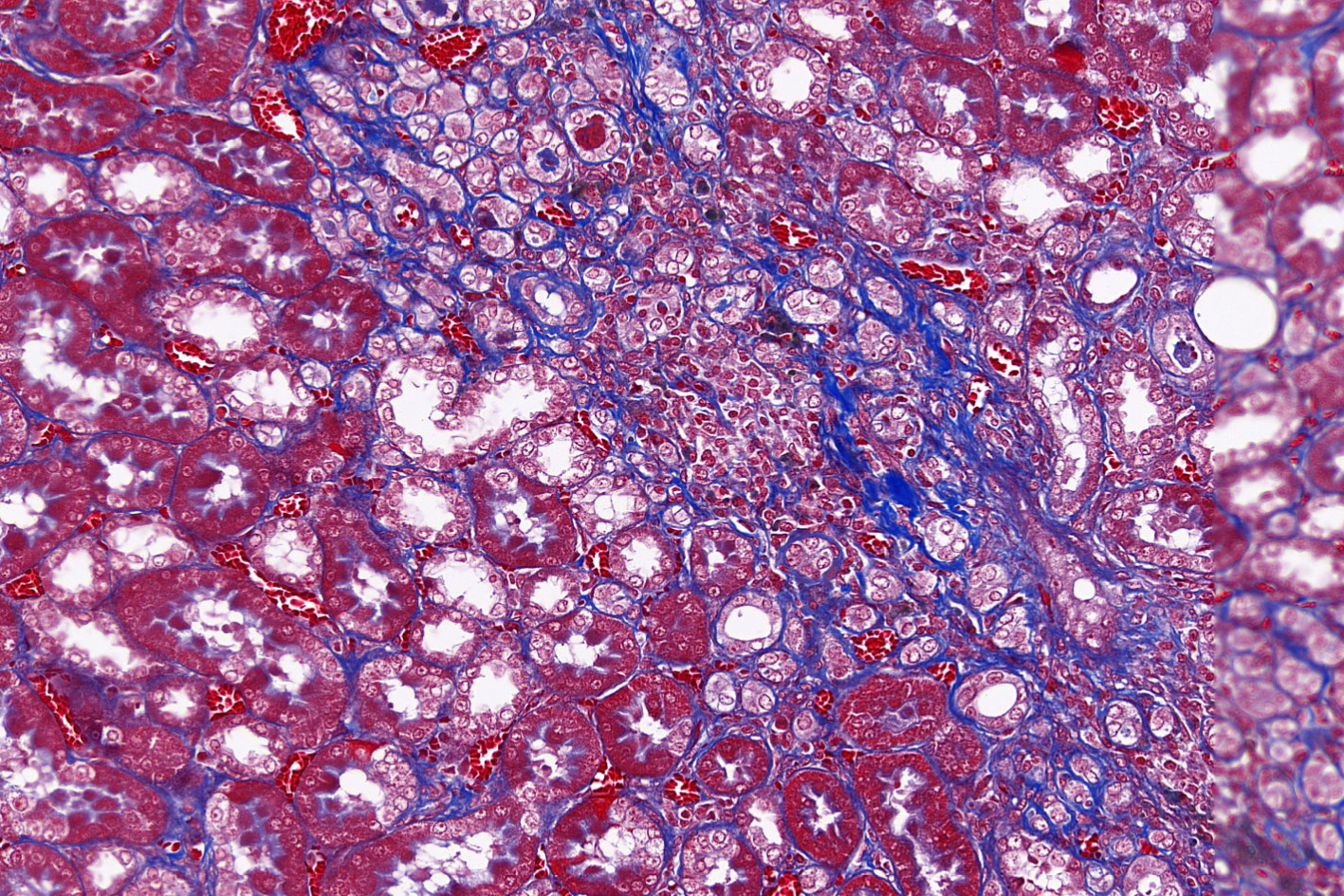

Supplement: Supplementary file 3 [file DataSheet1.ZIP › original files for Fig.1/Fig.1F Masson-DN16W.jpg]

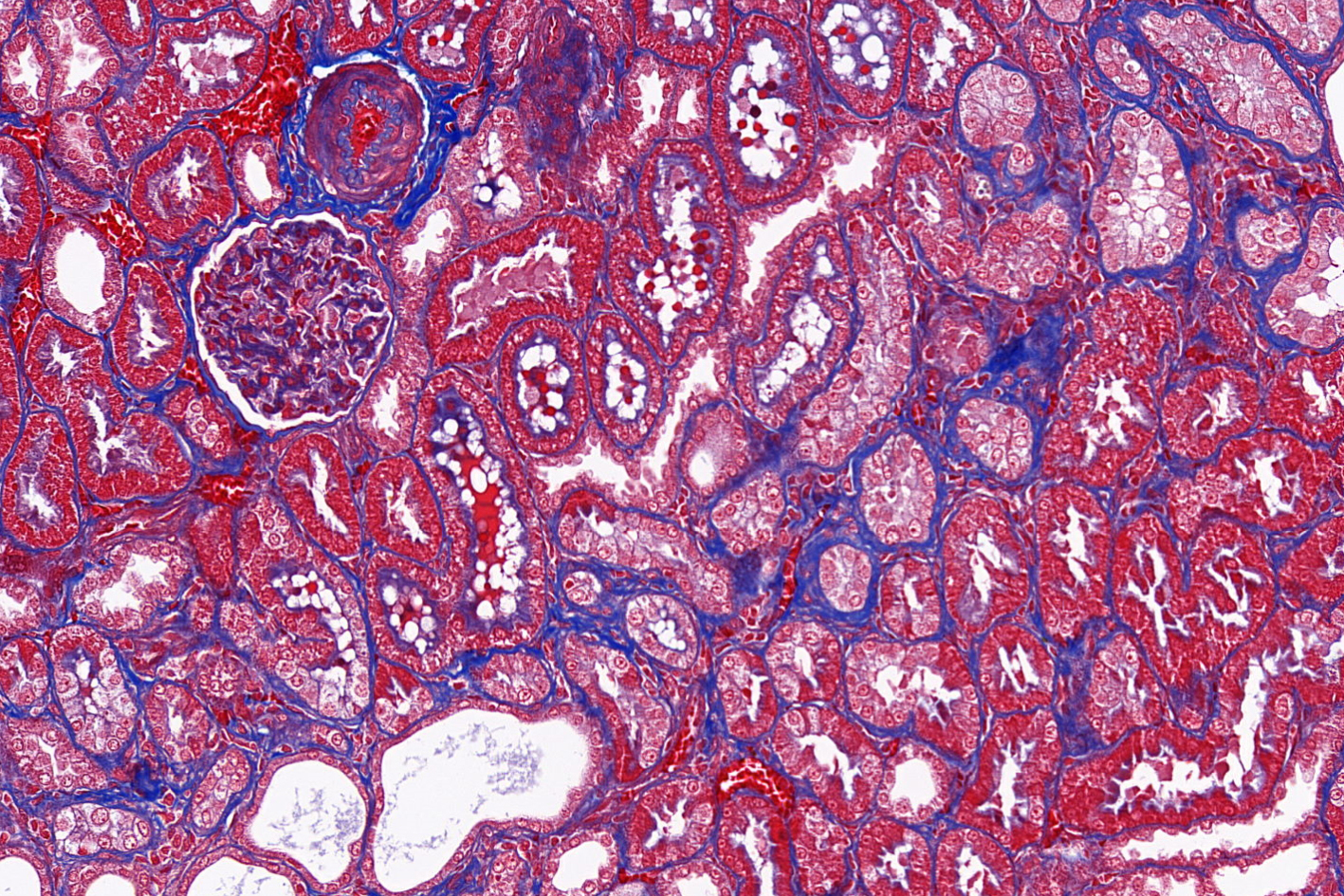

Supplement: Supplementary file 3 [file DataSheet1.ZIP › original files for Fig.1/Fig.1F Masson-DN8W.jpg]

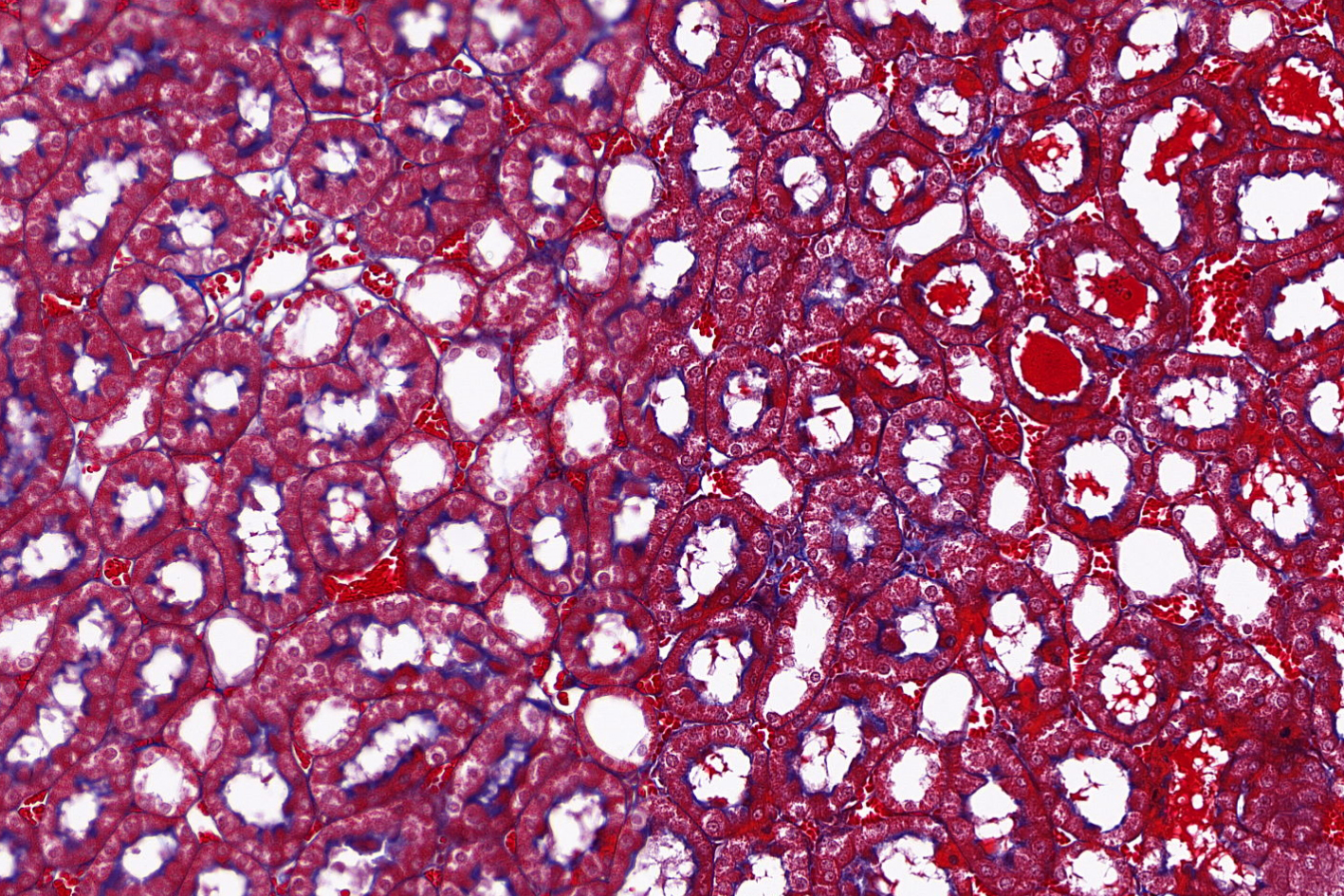

Supplement: Supplementary file 3 [file DataSheet1.ZIP › original files for Fig.1/Fig.1F Masson-control.jpg]

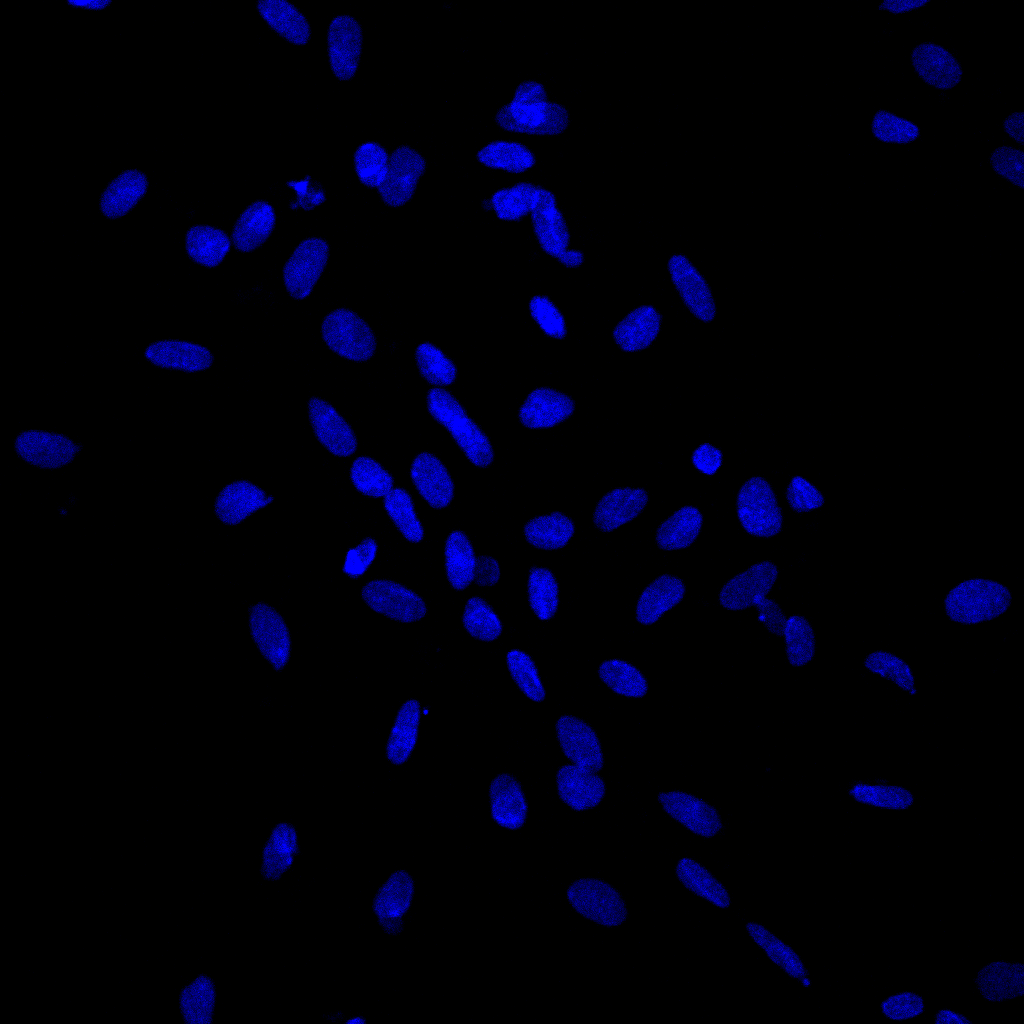

Supplement: Supplementary file 4 [file DataSheet2.ZIP › original files for Fig.2/Fig.2A DAPI.tif]

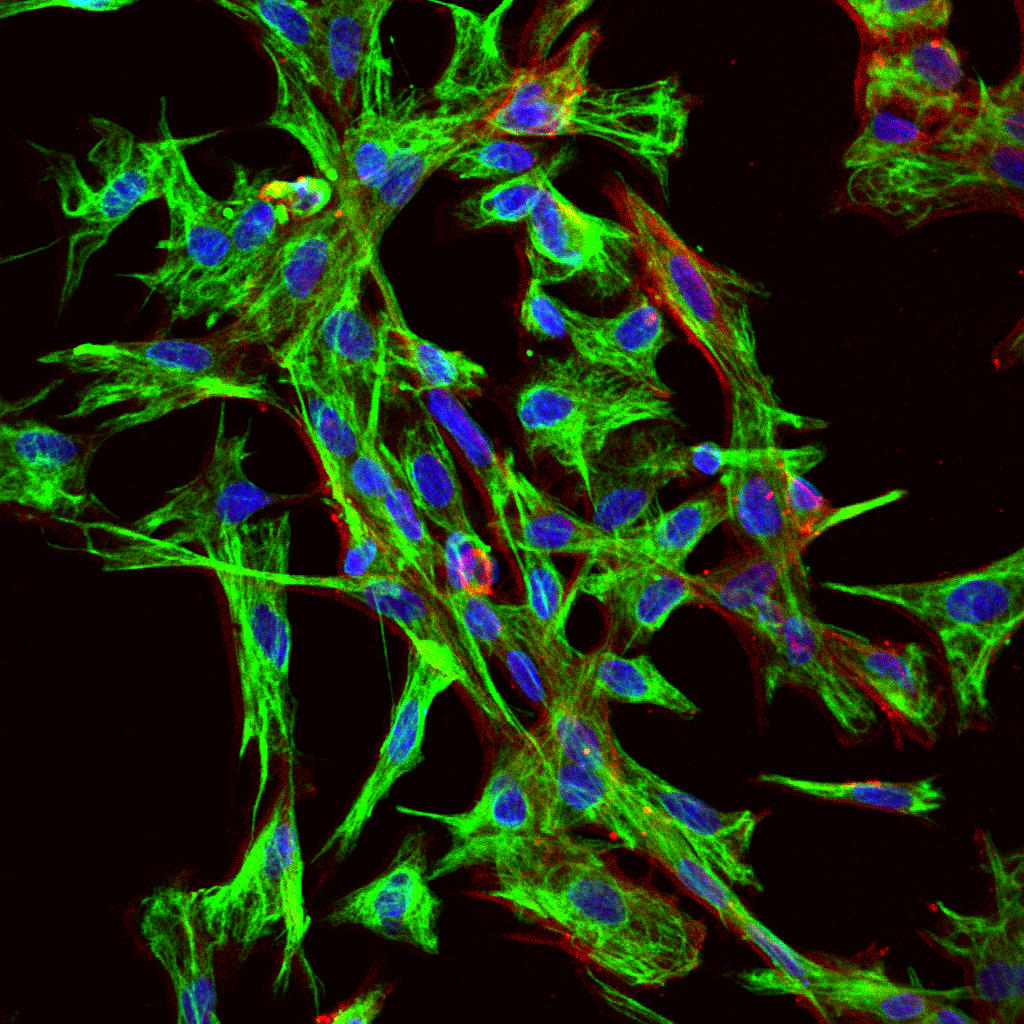

Supplement: Supplementary file 4 [file DataSheet2.ZIP › original files for Fig.2/Fig.2A Merged.tif]

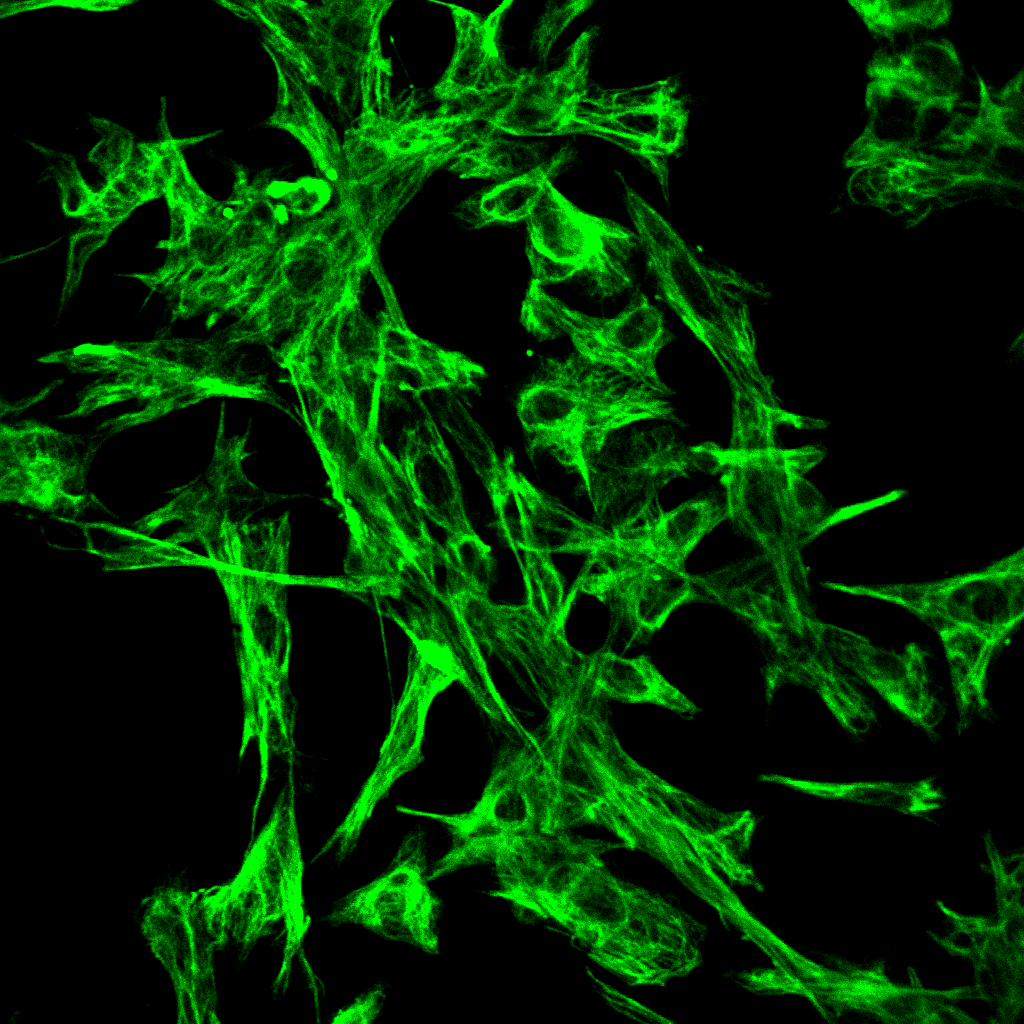

Supplement: Supplementary file 4 [file DataSheet2.ZIP › original files for Fig.2/Fig.2A Vimentin.tif]

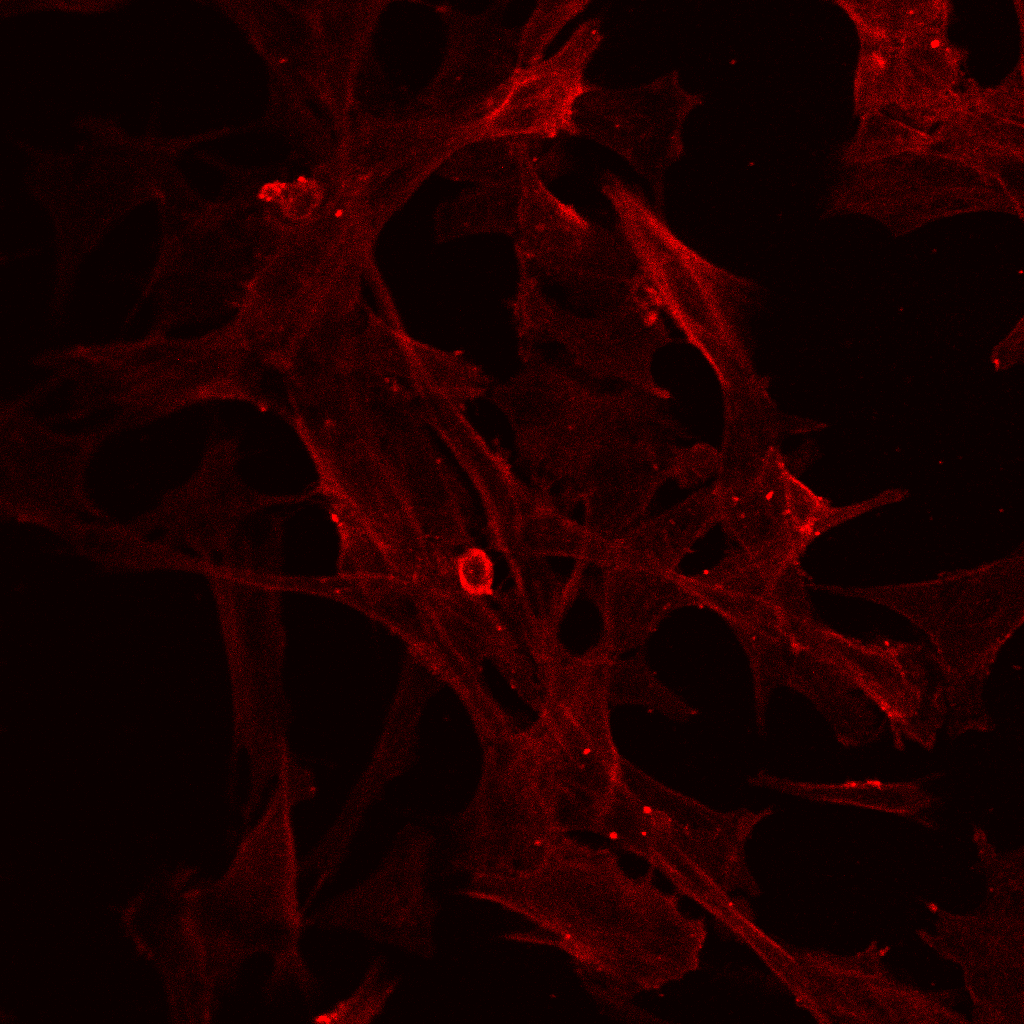

Supplement: Supplementary file 4 [file DataSheet2.ZIP › original files for Fig.2/Fig.2A a-SMA.tif]

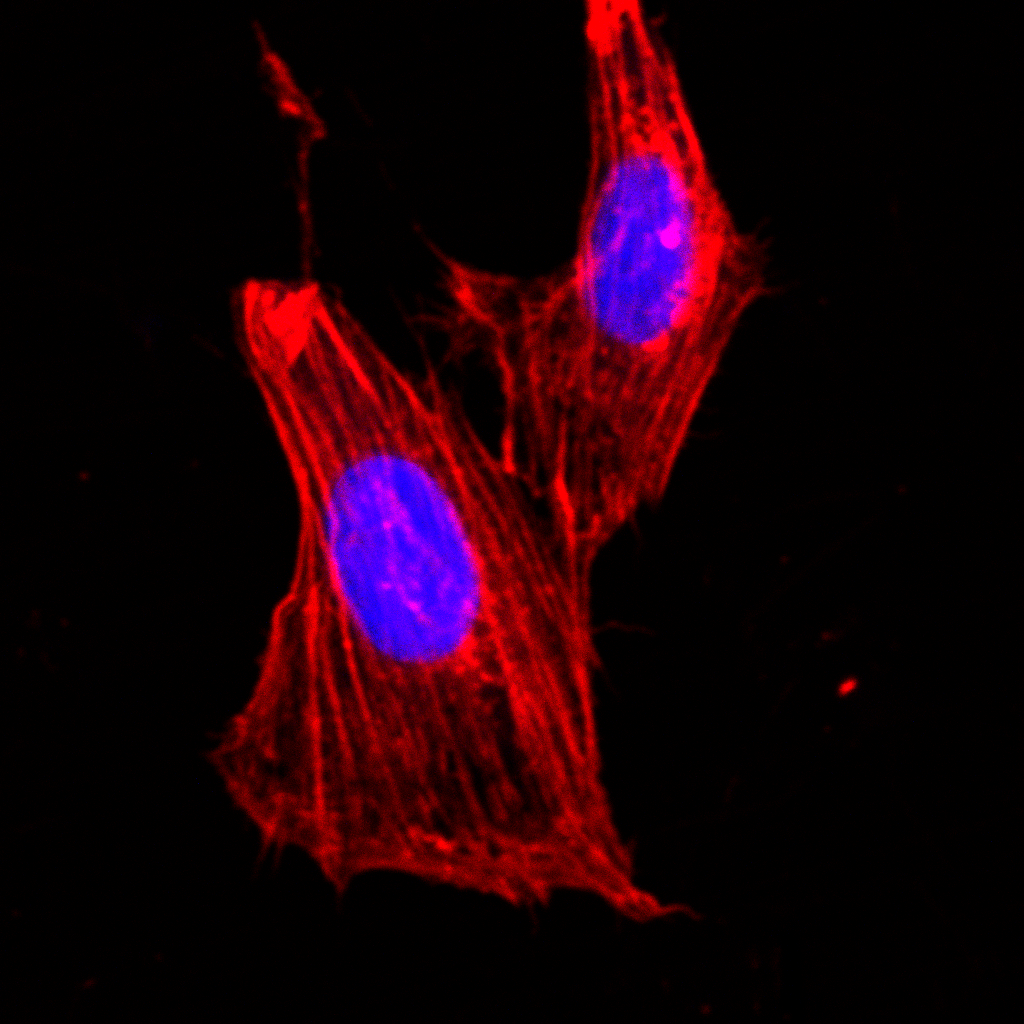

Supplement: Supplementary file 4 [file DataSheet2.ZIP › original files for Fig.2/Fig.2C 12 kPa.tif]

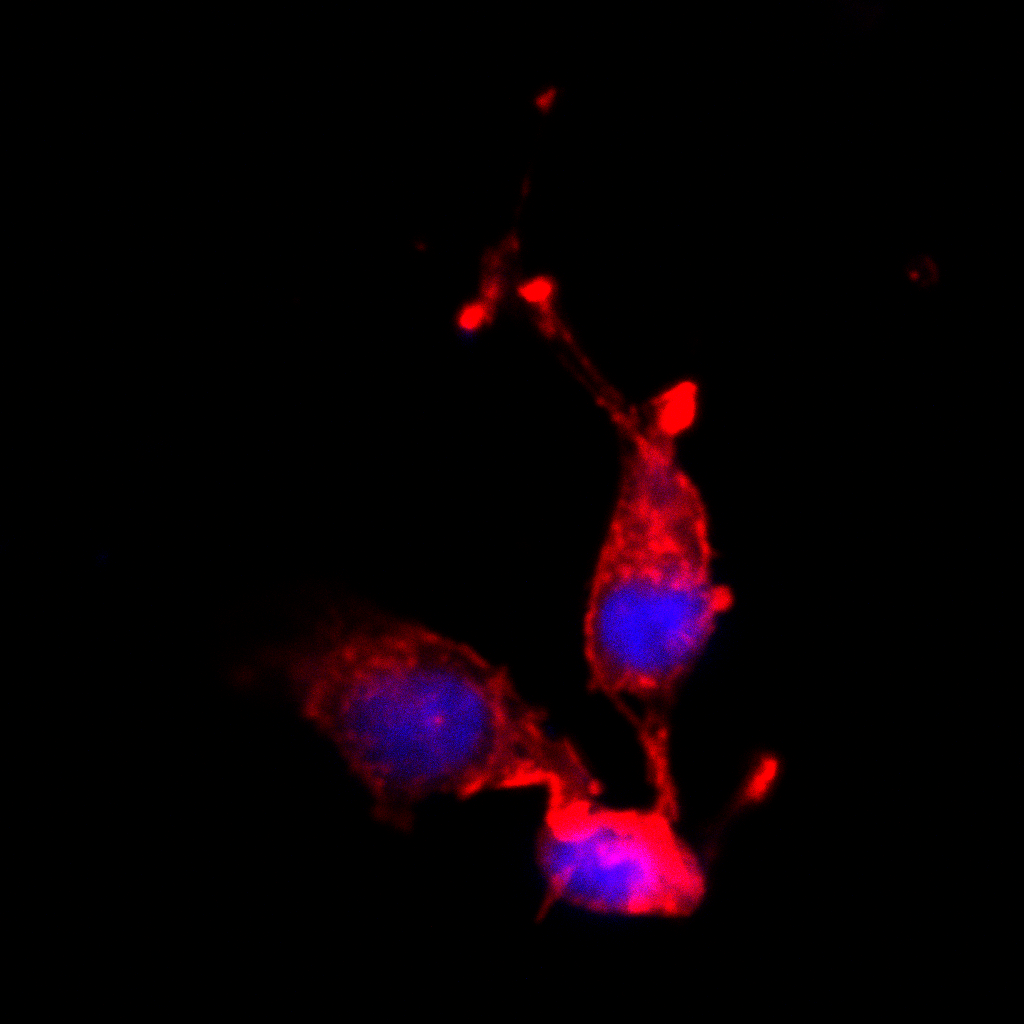

Supplement: Supplementary file 4 [file DataSheet2.ZIP › original files for Fig.2/Fig.2C 3 kPa.tif]

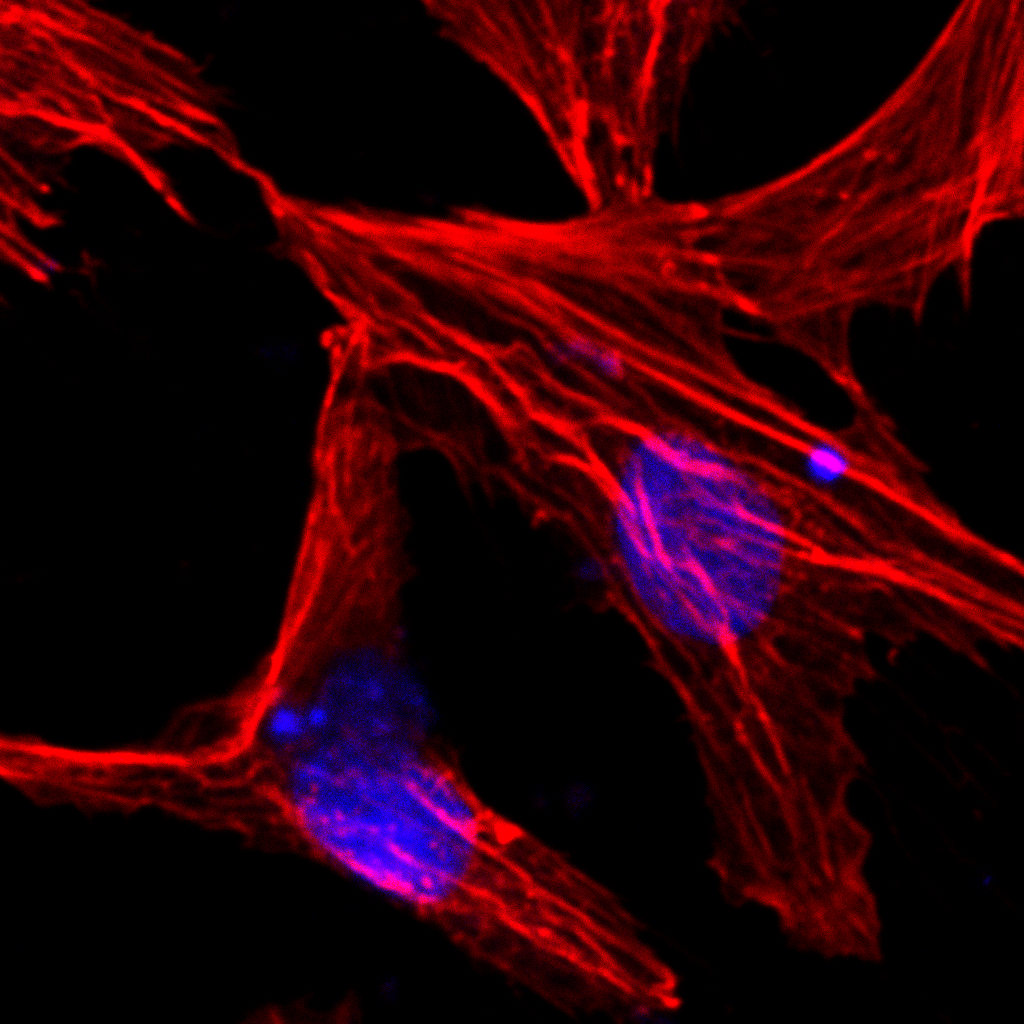

Supplement: Supplementary file 4 [file DataSheet2.ZIP › original files for Fig.2/Fig.2C 30 kPa.tif]
